# The Influence of Vasopressor-Induced Arterial Blood Pressure Elevation on Muscle-Recorded Motor Evoked Potentials

**DOI:** 10.1213/ANE.0000000000007701

**Published:** 2025-09-05

**Authors:** Sebastiaan E. Dulfer, Rob J. M. Groen, Fiete Lange, Katalin Tamasi, Frits H. Wapstra, Lilian M. Mennink, Bianca M. Dijkstra, Thouraya J. Dil, Christopher Faber, Anthony R. Absalom, Marko M. Sahinovic, Gea Drost

**Affiliations:** From the 1 Department of Neurosurgery, University Medical Center Groningen, University of Groningen, Groningen, the Netherlands; 2 Department of Neurosurgery, Faculty of Medicine Universitas Airlangga, Dr. Soetomo General Academic Hospital, Surabaya, Indonesia; 3 Department of Neurology, University Medical Center Groningen, University of Groningen, Groningen, the Netherlands; 4 Department of Epidemiology, University Medical Center Groningen, University of Groningen, Groningen, the Netherlands; 5 Department of Orthopedics, University Medical Center Groningen, University of Groningen, Groningen, the Netherlands; 6 Department of Anesthesiology, University Medical Center Groningen, University of Groningen, Groningen, the Netherlands.

## Abstract

**BACKGROUND:**

Transcranial electrical stimulation muscle-recorded motor evoked potentials (Tc-mMEPs) are used to monitor the spinal cord motor tracts during spinal surgery. There is considerable intra- and interindividual variability in the signals recorded, causing a high incidence of false positive warnings. Inadequate blood pressure is commonly blamed for false positive warnings and is usually managed with fluid and vasopressor therapy. The aim of the study was to systematically investigate the effects of norepinephrine and ephedrine-induced arterial blood pressure elevation on Tc-mMEPs.

**METHODS:**

Twenty-five patients undergoing spinal surgery were included in this prospective observational study. After anesthetic induction and positioning, but before incision, a norepinephrine infusion was used to increase the mean arterial pressure (MAP) from approximately 60 to 100 mm Hg. Tc-mMEP amplitudes and area under the curves (AUC) were recorded from the abductor hallucis (AH), tibialis anterior (TA), and hand muscles every 2 minutes. Voltage thresholds of the AH, TA, and hand muscles were determined at MAP values of 60, 80, and 100 mm Hg. The primary objective was to determine the effects of increasing the MAP with a vasopressor infusion on Tc-mMEP amplitude, AUC, and threshold. For the secondary objective, the outcomes were adjusted for depth of anesthesia and propofol concentrations. Post hoc analyses included adjusting for confounders, noradrenaline infusion rate, and use of ephedrine, investigating the effects of cardiac index on Tc-mMEP characteristics, investigating the influence of increasing MAP on the excitability of the peripheral nervous system, and investigating the effects of MAP on BIS.

**RESULTS:**

In the AH, TA, and hand muscles, Tc-mMEP amplitudes and AUC were significantly associated with MAP (*P* < .02). A 10 mm Hg increase of the MAP was associated with a 11.0% to 17.7% increase in the amplitude and a 10.5% to 16.8% increase in the AUC. When adjusting for BIS, propofol concentrations, and use of ephedrine, MAP remained only significantly associated with the Tc-mMEP amplitude (*P* = .002) and AUC (*P* = .003) of the AH muscles. The influence of cardiac index on Tc-mMEP amplitude and AUC provided similar results when compared to the influence of MAP on Tc-mMEP amplitude and AUC. No influence of increasing MAP on the excitability of the peripheral nervous system was found. Increasing MAP significantly increased BIS values, even when corrected for propofol concentrations.

**CONCLUSIONS:**

Elevation of MAP is associated with significantly higher Tc-mMEP amplitudes, AUCs, and lower voltage threshold. When corrected for BIS, propofol concentration, and use of ephedrine, the associations between increasing MAP and Tc-mMEP characteristics were largely attenuated. BIS values significantly increased by increasing MAP, when corrected for propofol concentration. Thereby, our results imply that increasing MAP increases the cortical excitability.

**KEY POINTS**

- **Question:** What are the effects of vasopressor-induced arterial blood pressure elevation on transcranial electrical stimulation muscle-recorded motor evoked potentials (Tc-mMEP)?
- **Findings:** Elevation of the mean arterial pressure is associated with significantly higher Tc-mMEP amplitudes, area under the curves, and lower voltage threshold.
- **Meaning:** Elevation of the mean arterial pressure increases Tc-mMEP amplitude and area under the curves (AUC), possibly due to an increase in cortical excitability.

Intraoperative neurophysiological monitoring (IONM) enables early detection and prevention of neurological injury during spinal surgery.^1^ Muscle-recorded transcranial electrical stimulation motor evoked potentials (Tc-mMEPs) are used to monitor the motor tracts during spinal surgery.^2^ Two main methods of evaluation of Tc-mMEP signals are used. The “threshold-level” method uses the motor threshold (ie, lowest stimulation voltage or current necessary to evoke a Tc-mMEP) as a predictor of neurological injury.^3,4^ With this method, an increase in the motor threshold of 100 volts or more, persisting for <1 hour, is used as a warning criterion for emergent postoperative motor weakness.^3,4^ For the “amplitude reduction” method, a 50% to 80% reduction from the Tc-mMEP baseline amplitude is usually interpreted as a warning of possible or impending neurological injury.^2,5^ The amplitude reduction method can be used with either submaximal or supramaximal transcranial stimulation.^6,7^

Whereas the methods mentioned above focus on threshold and amplitude, a more global Tc-mMEP characteristic is the area under the curve (AUC), a composite of amplitude (voltage) and signal duration/latency (time). The AUC provides a general estimate of cortico-spinal excitability, with a decrease in the Tc-mMEP AUC corresponding with a decrease in excitability.^8^ The AUC is usually not used for monitoring in clinical practice, and so far, no AUC warning criteria have been established.

Even though these monitoring methods are capable of detecting neurological injury and improving neurological outcomes, their clinical utility is sometimes limited by their propensity to give false positive warning signals, that is, warning signals in the absence of neurological injury.^2,9^ These false positives can arise due to technical issues or changes in physiological and pharmacological factors influencing the Tc-mMEPs.^10–16^

Spinal cord perfusion influences Tc-mMEPs, but unfortunately, there are no clinically available methods to monitor spinal cord perfusion directly.^17,18^ During surgery, anesthesiologists measure and modify the arterial blood pressure to ensure adequate tissue perfusion pressure. When warnings occur suggesting impending neurological injury, attempts are usually made to exclude inadequate spinal cord perfusion as a cause.^9^ Inadequate perfusion may be caused by hypotension, which can be corrected by optimization of hypnotic and analgesic drug doses and intravascular volume status. If hypotension persists, vasopressors such as norepinephrine can be administered. Norepinephrine should be administered judiciously as it increases blood pressure through vasoconstriction, which can subsequently decrease tissue perfusion.^11,19,20^ Although blood pressure elevation is often observed to restore Tc-mMEP amplitudes, the exact effects of vasopressor-induced elevation of blood pressure on Tc-mMEPs in humans have not yet been well described.^9,16,21^

The primary goal of this study was to determine the influence of norepinephrine and ephedrine-induced arterial blood pressure elevation on the Tc-mMEP amplitudes, AUCs, and thresholds.

## METHODS

This was a prospective observational study. The study protocol was approved by the Medical Ethical Committee of the University Medical Center Groningen (METc nr 2018.630, ABR nr NL68223.042.18), prospectively registered in the Dutch Trial Register (NL7772), and has been published elsewhere.^22,23^ Written informed consent was obtained from all subjects, a legal surrogate, the parents or legal guardians for minor subjects.

### Patients

Twenty-five patients were included. Inclusion and exclusion criteria have been previously published.^23^

### Study Objectives

The primary objective was to determine the effects of increasing the mean arterial pressure (MAP) with a norepinephrine infusion on Tc-mMEP amplitude, AUC, and threshold during spinal surgery.

Also, the influence of MAP elevation on Tc-mMEP amplitude and AUC was adjusted for the possible effects of depth of anesthesia (quantified by the Bispectral index value(BIS)) and estimated plasma propofol concentrations. For the secondary objective, the influence of MAP elevation on Tc-mMEP voltage threshold was adjusted for BIS and measured plasma propofol concentrations.

As post hoc objectives, first, we investigated if norepinephrine infusion rate and administering a bolus of ephedrine affected the association for the secondary objective for the Tc-mMEP amplitude and AUC.

Second, we investigated the influence of cardiac index (L.min^-1^.m^-2^) on Tc-mMEP amplitude and AUC for both the primary and secondary objectives. We also investigated if norepinephrine infusion rate and administering a bolus of ephedrine affected the association for the secondary objective.

Third, we evaluated the effects of MAP on BIS, and the effects of MAP and ephedrine on BIS, adjusted for estimated plasma propofol concentration. Moreover, measured plasma propofol concentrations were plotted against the different MAP values at which the blood samples were taken.

**Figure 1.**
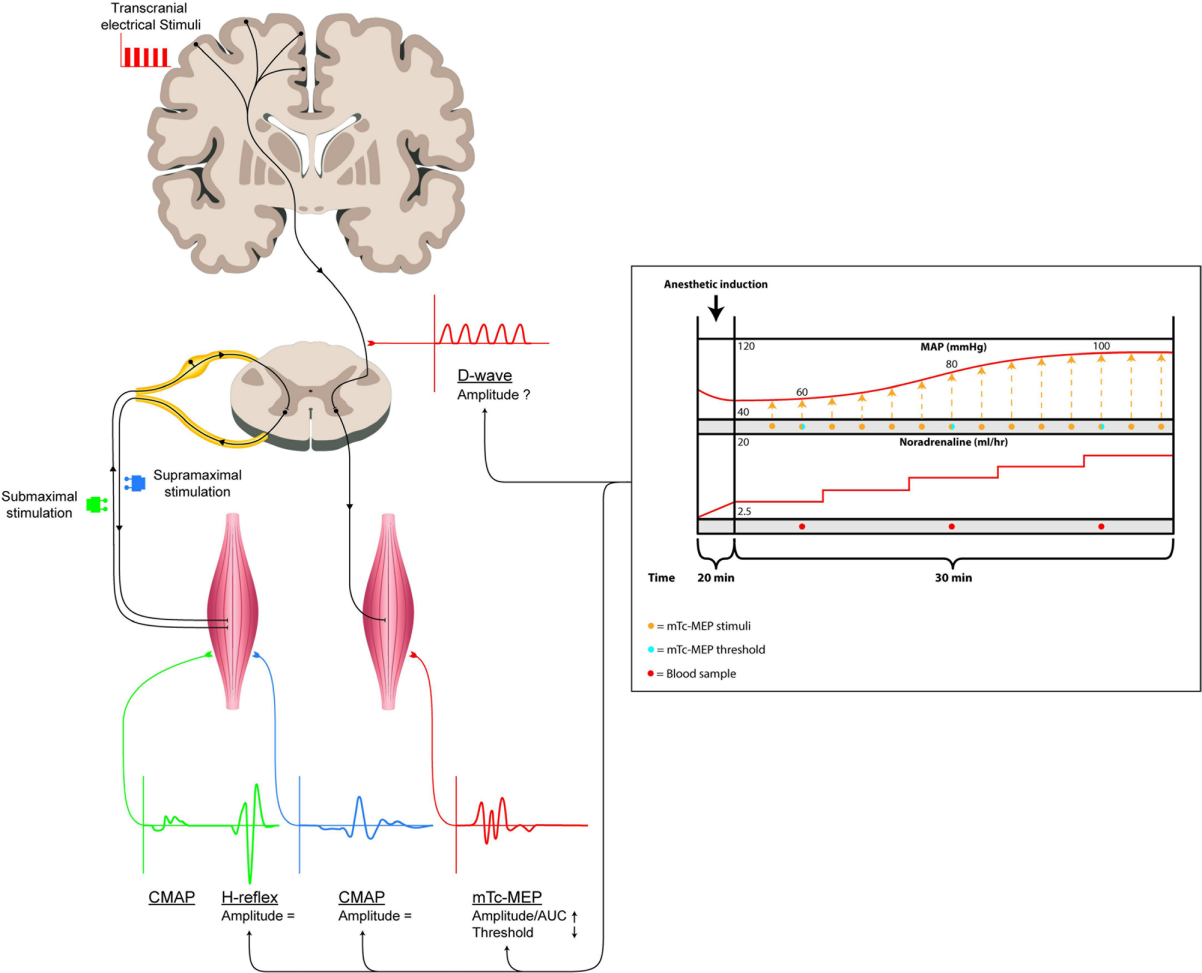
Schematic overview of effects of increasing MAP with a vasopressor infusion on Tc-mMEP amplitude, AUC and threshold, H-reflex amplitude and CMAP amplitude The effects of increasing MAP with norepinephrine (right schematic study protocol within black box) have no significant effect on H-reflex and CMAP amplitude. Increasing MAP with norepinephrine was significantly associated with an increase of the Tc-mMEP amplitude and AUC, and a decrease of the Tc-mMEP voltage threshold. The effects of increasing MAP with norepinephrine on D-waves are unknown.

Fourth, we evaluated the influence of increasing MAP with a vasopressor infusion on the excitability of the peripheral nervous system; the effects of increasing MAP on the Hoffman-reflex (H-reflex) amplitude and compound muscle action potential (CMAP) amplitude. The H-reflex is a monosynaptic reflex obtained by submaximal electrical stimulation of peripheral sensory fibers. CMAPs are evoked by supramaximal peripheral motor nerve stimulation (Figure 1).

Lastly, to assess possible late effects of the muscle relaxants after repetitive CMAP stimulation, CMAP 5-to-1 ratios were calculated, comparable to the train of four (TOF) ratio monitoring.

### Anesthetic Management

All patients underwent a preoperative screening assessment by an anesthesiologist. An IV cannula was sited on a hand or forearm, and a 20G cannula was inserted in a radial artery. Initial fluid administration consisted of a continuous IV infusion (500 cm^3^.h^-1^) of a crystalloid solution. Anesthetic induction and maintenance were by total intravenous anesthesia (TIVA) using propofol and remifentanil administered by target-controlled infusion (TCI) using Alaris PK plus pumps (Becton Dickinson) programmed with the Schnider and Minto models, respectively.^24^ Depth of anesthesia was monitored with a processed electroencephalogram monitor (BIS, Medtronic, Dublin, Ireland). Remifentanil target concentration was 4 ng.mL^–1^ and the propofol target concentration was adjusted to maintain a BIS between 40 and 60. As reported elsewhere, other parameters that might influence Tc-mMEP measurements were kept stable and within specified ranges.^23^

The cardiac output and cardiac index were measured continuously using the FloTrac/EV1000 system (Edwards Lifesciences).

A single dose of rocuronium 0.5 mg.kg^-1^ was administered before endotracheal intubation. No further doses were administered to avoid possible adverse effects of muscle relaxation on the Tc-mMEPs. The average time between rocuronium administration and the first Tc-mMEP measurement was 1 hour and 14 minutes (minimum 1 hour, maximum 1 hour and 41 minutes). Therefore, no late effects of rocuronium on the Tc-mMEPs were expected.

After anesthetic induction, and before start of the study, the mean arterial pressure was not actively managed unless it fell below 60 mm Hg, in which case the patient was placed in a shallow Trendelenburg position, and if necessary additional fluids and/or a low dose infusion of norepinephrine were administered (ranging from 0 to 0.33 µg/min^–1^).

### Transcranial Electrical Stimulation Muscle-Recorded Motor Evoked Potentials

The Tc-mMEP monitoring parameters have been published previously.^23^ Tc-mMeps for the lower extremities were recorded using surface electrodes placed on the Ta and AH muscles. For the upper extremities, surface electrodes were placed bilaterally on the abductor pollicis brevis and abductor digiti minimi muscles, producing a combined hand muscle response. The equipment and monitoring settings used in this study are summarized in Table 1. Supramaximal stimulation was attempted. The motor thresholds were determined by visual inspection. The H-reflex of the left and right gastrocnemius muscle was recorded using submaximal stimulation of the tibial nerve. CMAPs were recorded at the TA and AH left and right, after supramaximal stimulation of the tibial and peroneal nerves. Pulse durations were optimized to achieve supramaximal CMAP amplitudes and ranged from 0.2 to 0.5 ms.

**Table 1.** mTc-MEP Monitoring Settings

Parameter settings	
Equipment	NIM-Eclipse E4 IONM system (Medtronic BV)
Stimulator	Constant voltage
Monophasic/biphasic stimulation	Biphasic stimulation
Stimulation method	Supramaximal stimulation
Stimulation location	Cpl1–Cpl2 (1 cm posterior and 1 cm lateral to C1 and C2)
Pulse duration	0.075 ms
Number of pulses	5 pulses
Interstimulus interval	Optimized between 1 ms and 4 ms
Stimulation electrodes	Corkscrew electrode (Medtronic, Xomed)
Recording electrodes	Surface electrode: 20 mm × 27 mm, adhesive surface pad electrodes (Medtronic, Xomed)

Lastly, CMAP amplitude 5-to-1 ratios were calculated every 2 minutes after 5 biphasic supramaximal stimuli. The stimulation current was applied at 0.8 Hz (1.0 Hz in 1 patient).

### Study Procedure

Study measurements began after anesthetic induction and positioning of the patient, but before surgical incision. At this stage, MAP was at, or slightly >60 mm Hg in all patients. A norepinephrine infusion (20 µg.mL^–1^) was then used to elevate the MAP gradually, aiming to increase it from 60 to 100 mm Hg >30 minutes. Infusion rates administered were at the discretion of the responsible anesthesiologist, but in general, the starting infusion rate was 0.83 µg.min^–1^, and after that, the infusion rate was increased every 3 minutes until a maximum of 6.67 µg.min^–1^ was reached or the MAP reached 100 mm Hg. The maximum norepinephrine infusion rate of 6.67 µg.min^–1^ was chosen to avoid possible adverse effects.^25^ In eleven patients, 1 or multiple ephedrine boluses of 2.5 mg or 5 mg were administered at the end of the study measurements to achieve an MAP of 100 mm Hg.

Every 2 minutes, Tc-mMEPs, H-reflex, CMAPs, and CMAP 5-to-1 ratios were recorded. Voltage motor thresholds of the TA and AH muscles were measured at MAPs of approximately 60, 80, and 100 mm Hg. Three blood samples for subsequent plasma propofol concentration assays were taken when the MAP was approximately 60, 80, and 100 mm Hg.

The arterial blood samples were collected in EDTA (ethylenediaminetetraacetic acid) tubes and stored at room temperature (<60 minutes) before they were centrifuged for 8 minutes at 3000 rpm. Plasma was transferred into vials and stored at −80ºC. Ultrahigh-performance liquid chromatography mass spectrometry (TSQ-Quantiva, Thermo Scientific) in atmospheric pressure chemical ionization mode was performed to determine propofol plasma concentrations.

### Data Collection

Anesthetic data was automatically downloaded every 15 seconds by the electronic patient record system. Anesthetic variables of interest were subsequently extracted from the hospital information system. MAP, BIS, and Cardiac Index values were smoothed using a moving median filter of 265 seconds to filter out artifacts (ie, flushing after taking blood samples). Individual plots of the time course of anesthetic variables, amplitudes, and AUCs were prepared per variable (Supplemental Digital Content 1, Supplementary Material 1, https://links.lww.com/AA/F424). Visual inspection was performed to identify artifacts and missing data. The times at which ephedrine boluses were administered were collected. All Tc-mMEP measurements after administration of an ephedrine bolus were scored as “yes” and before as “no.”

Tc-mMEP, H-reflex, and CMAP curves were exported from the NIM-Eclipse E4 IONM system. After that, amplitudes and AUCs were calculated using software written in Python (version 3.7.1). The consecutive Tc-mMEP, H-reflex, and CMAP amplitudes and Tc-mMEP AUCs were changed to proportions of the first measurement per patient and muscle.

### Statistical Analysis

Analyses were performed with R Software version 4.0.5 (The R Foundation for Statistical Computing).

For the primary and secondary objectives, a mixed-effects model analysis was used to account for the repeated Tc-mMEP measurements. As a sensitivity analysis, orthogonal polynomial coding was performed in which linear and higher-level terms (ie, quadratic, cubic, etc) for the MAP were fitted.

As a post hoc analysis, we assessed whether norepinephrine and ephedrine were confounders for the secondary objective. Moreover, we investigated the influence of cardiac index on Tc-mMEP characteristics. In a further post hoc analysis, linear mixed effects model analyses with the H-reflex amplitude and CMAP amplitude as outcomes separately, instead of the Tc-mMEP amplitude, were performed for both the primary and secondary objectives.

**Table 2.** Patient Characteristics

Patient characteristics	Patients (n = 25)	
Age at surgery in y, mean (SD)	23.4 (10.5)	
Female N patients (%)	21 (84.0)	
Length in cm mean (SD)	172.5 (8.7)	
Weight in kg mean (SD)	70.6 (17.2)	
Diagnosis N patients (%) Idiopathic scoliosis Cervical intradural meningioma C1 ATSCH Th-2 Intradural Schwannoma conus Intramedullary cavernoma Th9 Tethered cord redetethering Intramedullary cyst C6-Th3 Myxopapillary ependymoma Th12-L1	17 (68.0) 2 (8.0) 1 (4.0) 1 (4.0) 1 (4.0) 1 (4.0) 1 (4.0) 1 (4.0)	
Surgery time in minutes mean (SD)	307 (84)	
**mTc-MEP characteristics**		
Interstimulus interval N patients (%)		
1.5 ms	19 (76.0)	
1 ms	6 (24.0)	
Voltage intensity median (IQR)	340 (280–380)	
Elicitability N patients (%)		
TAL	25 (100.0)	
TAR	24 (96.0)	
AHL	25 (100.0)	
AHR	24 (96.0)	
HANDL	24 (100.0)	
HANDR	25 (96%)	
Number of mTc-MEP amplitudes/AUCs Median (IQR)	13 (13–14)	
Voltage threshold in V TAL mean (SD)		
MAP60 mm Hg	193.6 (49.4)	
MAP80 mm Hg	192.4 (46.1)	
MAP100 mm Hg	184.8 (43.5)	
Voltage threshold in V TAR mean (SD)		
MAP60 mm Hg	179.6 (46.2)	
MAP80 mm Hg	176.8 (46.4)	
MAP100 mm Hg	176.0 (47.4)	
Voltage threshold in V AHL mean (SD)		
MAP60 mm Hg	184.4 (51.7)	
MAP80 mm Hg	183.6 (50.2)	
MAP100 mm Hg	180.4 (49.5)	
Voltage threshold in V AHR mean (SD)		
MAP60 mm Hg	179.2 (47.8)	
MAP80 mm Hg	175.6 (46.4)	
MAP100 mm Hg	172.8 (44.7)	
Voltage threshold in V HANDL mean (SD)		
MAP60 mm Hg	152.4 (45.5)	
MAP80 mm Hg	146.3 (39.5)	
MAP100 mm Hg	146.3 (43.3)	
Voltage threshold in V HANDR mean (SD)		
MAP60 mm Hg	136.4 (41.2)	
MAP80 mm Hg	130.8 (32.9)	
MAP100 mm Hg	131.6 (41.6)	
**Pharmacological and physiological parameters**		**Missing data (%**)
Estimated plasma propofol concentration µg.mL^–1^ Median (IQR)	2.6 (2.2–2.8)	2.3
Remifentanil Ce ng.mL^–1^ Median (IQR)	4 (4.0–4.0)	2.3
MAP mm Hg median (IQR)	83 (71–92)	0.0
BIS median (IQR)	47 (41–53)	4.3
Spo_2_ % median (IQR)	98.5 (98.0–99.4)	0.0
Temperature °C median (IQR)	36.1 (35.9–36.3)	3.3
Heartrate bpm median (IQR)	54 (48–61)	0.0
Cardiac output L.min^–1^ Median (IQR)	4.2 (3.4–5.3)	3.6
Cardiac index L.min.m^2^	2.4 (1.8–3.0)	3.6
Stroke volume variation % Median (IQR)	11.3 (9.0–12.8)	3.6
End-tidal CO2 kPa median (IQR)	4.6 (4.3–4.8)	0.0
Ephedrine bolus N patients (%)^a^	11 (44.0)	0.0

Abbreviations: AH, abductor halluces; ATSCH, anterior thoracic spinal cord herniation; Ce, estimated plasma concentrations; mTc-MEP, muscle-recorded transcranial electrical stimulation motor evoked potential; TA, tibialis anterior.

a Ephedrine boluses were either 2.5 mg or 5 mg, and were sometimes repeated.

A pragmatic sample size of 25 patients was chosen as reported in the published study protocol.^23^

## RESULTS

### Patients

Twenty-five patients were included from April 2019 until September 2021. Their characteristics are shown in Table 2. In 1 patient, Tc-mMEPs of the AH right, in 1 patient the Tc-mMEPs of the TA right, and in another patient the Tc-mMEPs and thresholds of the left hand muscles could not be obtained. These measurements were therefore not included in the analysis.

### Primary Objective

There were significant associations between MAP and Tc-mMEP amplitudes and MAP and Tc-mMEP AUC (*P* < .02; Table 3). A 10 mm Hg increase of the MAP was associated with a 11.0% to 17.7% increase in the amplitude and a 10.5% to 16.8% increase in the AUC.

**Table 3.** Results of the Mixed-Effects Model Analyses for the Effects of Increasing the MAP With a Vasopressor Infusion on mTc-MEP Amplitude, AUC and Threshold

Outcome		Coefficient	95% CI	*P* value
Amplitude^a,b^ (proportion of baseline)	MAP (mm Hg)			
	AH	1.77	0.99–2.55	**<.001**
	TA	1.11	0.33–1.89	**.019**
	HAND	1.10	0.32–1.88	**.006**
AUC^a,b^ (proportion of baseline)	MAP (mm Hg)			
	AH	1.68	0.94–2.43	**<.001**
	TA	1.05	0.30–1.79	**.006**
	HAND	1.05	0.30–1.79	**.006**
Threshold^a^ (V)	MAP60 mm Hg	Reference category		
	MAP80 mm Hg	–3.22	–7.76 to 1.31	.166
	MAP100 mm Hg	–5.51	–10.05 to −0.97	**.018**

The estimates for amplitude and AUC are percentages (1 mm Hg gives 1.77% in mTc-MEP AH amplitude). Significant coefficients are shown in bold text.

Abbreviations: AUC, area under the curve; MAP, mean arterial pressure; V, voltage.

a Corrected for side and muscle. Patient was added as random intercept and MAP was added as random slope.

b Interaction between muscle and MAP was added.

The mean and standard deviations of the voltage thresholds can be found in Table 2.

**Table 4.** Results of the Mixed Effects Model Analyses for the Effects of Increasing the MAP With a Vasopressor Infusion on mTc-MEP Amplitude, AUC and Threshold Adjusted for BIS and Plasma Propofol Concentration

Outcome		Coefficient	95% CI	*P* value
Amplitude^a,b^ (proportion of baseline)	MAP (mm Hg)			
	AH	1.14	0.43–1.86	**.002**
	TA	0.37	–0.34 to 1.08	0.305
	HAND	0.30	–0.41 to 1.01	0.409
AUC^a,b^ (proportion of baseline)	MAP (mm Hg)			
	AH	0.99	0.34–1.64	**.003**
	TA	0.27	–0.37 to 0.92	.401
	HAND	0.23	–0.42 to 0.87	.497
Threshold^c^ (V)	MAP60 mm Hg	Reference category		
	MAP80 mm Hg	–1.95	–6.70 to 2.80	.425
	MAP100 mm Hg	–2.53	–8.65 to 3.60	.422

The estimates for amplitude and AUC are percentages (1 mm Hg gives 0.84% in mTc-MEP amplitude). Significant coefficients are shown in bold text.

Abbreviations: AUC, area under the curve; MAP, mean arterial pressure; V, voltage.

a Corrected for side, muscle, BIS and estimated plasma propofol concentration. Patient was added as random intercept and MAP was added as random slope.

b Interaction between muscle and MAP was added.

c Corrected for side, muscle, BIS and measured plasma propofol concentration. Patient was added as random intercept and MAP was added as random slope.

Increasing the MAP from 60 mm Hg decreased the mean voltage thresholds by 3.22 V (confidence interval [CI], 7.76–1.31 V; *P* = .166) at an MAP of 80 mm Hg, and by 5.51V (95% CI, −10.05 to −0.97 V; *P* = .018) at an MAP of 100 mm Hg (Table 3). Orthogonal polynomial coding showed that our results were consistent with a linear relationship between MAP and amplitude as well as MAP and AUC.

### Secondary Objective

When the BIS and plasma propofol concentrations were added to the mixed effects model analysis, MAP only remained significantly associated with the Tc-mMEP amplitude and AUC of the AH muscles, but to a lesser extent (Table 4). With these variables added to the model, a 10 mm Hg increase in the MAP was associated with an increase in Tc-mMEP amplitude of the AH muscles of 11.4% (CI, 4.3%–18.6%; *P* = .002), and an increase in Tc-mMEP AUC of the AH muscles of 9.9% (CI, 3.4%–16.4%; *P* = .003). The associations between MAP and Tc-mMEP amplitudes and AUCs of the TA and hand muscles were no longer significant. Moreover, the association between MAP and Tc—mMEP voltage threshold was no longer statistically significant either (Table 4).

### Post Hoc Analysis

In a post hoc analysis, we assessed whether norepinephrine infusion rate (µg.min^-1^) and ephedrine boluses affected the association between MAP and Tc-mMEP amplitude and AUC, adjusted for BIS and estimated plasma propofol concentration. When both norepinephrine infusion rate and MAP were included in the mixed-effects model, the initially positive association between norepinephrine and mTc-MEP reversed to a negative relationship, suggesting potential collinearity. We therefore excluded norepinephrine infusion rate from the analyses.

When ephedrine boluses (yes/no) were added to the mixed effects model analysis, the association between MAP and the Tc-mMEP amplitude and AUC were comparable to the results of the secondary objective in which there was only a significant association between MAP and the mTc-MEP amplitude and AUC of the AH muscles. A 10 mm Hg increase in the MAP was associated with an increase in Tc-mMEP amplitude of the AH muscles of 9.4% (CI, 2.5%–16.4%; *P* = .008), and an increase in Tc-mMEP AUC of the AH muscles of 7.7% (CI, 1.5%–13.9%; *P* = .016; Table 5).

**Table 5.** Results of the Mixed-Effects Model Analyses for the Effects of Increasing the MAP With a Vasopressor Infusion on mTc-MEP Amplitude and AUC Adjusted for BIS, Estimated Plasma Propofol Concentration and Ephedrine

Outcome		Coefficient	95% CI	*P* value
Amplitude^a^ (proportion of baseline)	MAP (mm Hg)			
	AH	0.94	0.25–1.64	**.008**
	TA	0.18	-0.52–0.87	.619
	HAND	0.10	-0.59–0.80	.767
AUC^a^ (proportion of baseline)	MAP (mm Hg)			
	AH	0.77	0.15–1.39	**.016**
	TA	0.06	-0.56–0.68	.849
	HAND	0.013	-0.61–0.63	.968

The estimates for amplitude and AUC are percentages (1 mm Hg gives 0.94% in mTc-MEP amplitude of the AH muscle). Significant coefficients are shown in bold text.

Abbreviations: AUC, area under the curve; MAP, mean arterial pressure.

a Corrected for side, muscle, BIS, estimated plasma propofol concentration and ephedrine. Patient was added as random intercept and MAP was added as random slope. Interaction between muscle and MAP was added.

In the second post hoc analysis, we assessed the influence of cardiac index (L.min.m^2^) on Tc-mMEP amplitude and AUC.

An increase of 1 L.min.m^2^ in CI was associated with a 28.7% increase (CI, 11.0%–56.0%) of the Tc-mMEP amplitude (*P* = .004) and a 27.7% increase (CI, 7.9%–47.5%) of the Tc-mMEP AUCs (*P* = .006) of the TA, AH and hand muscles.

When adjusted for BIS and estimated plasma propofol concentration, the association between CI and Tc-mMEP amplitude and AUC remained but to a lesser extent: an increase of 1L.min.m^2^ was associated with a 20.1% increase (CI, 1.1%–39.2%) of the Tc-mMEP amplitude (*P* = .039) and with a 19.4% increase (CI, 0.5%–38.3%) of the Tc-mMEP AUC.

When also adjusted for ephedrine, the association between CI and Tc-mMEP amplitude and AUC was no longer significant (Supplemental Digital Content 2, Supplementary Table 1, https://links.lww.com/AA/F425).

**Figure 2.**
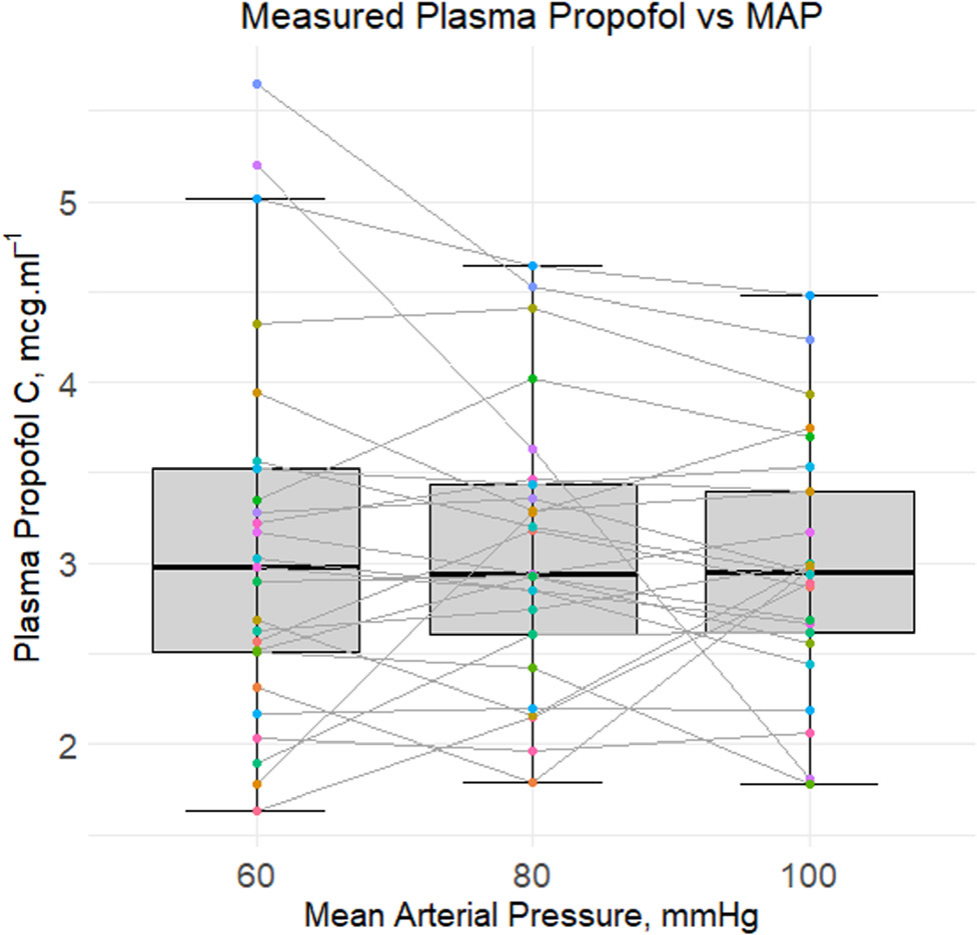
Boxplots of measured plasma propofol concentrations (µg.mL^–1^) per MAP category (60, 80, and 100 mm Hg). The middle line of each box is the median, the edges the 25th and 75th centiles and the whiskers the 10th and 90th centiles.

In Figure 2, the measured plasma propofol concentrations (µg.mL^–1^) per MAP category (60, 80, and 100 mm Hg) are shown in a boxplot, in which it can be observed that the measured plasma propofol concentrations remained stable.

The effects of increasing MAP on the H-reflex amplitude and CMAP amplitude for both the primary and secondary objectives were also analyzed. In 1 patient, H-reflexes were not elicitable.

For both objectives, MAP was not significantly associated with the H-reflex amplitude and CMAP amplitude (Supplemental Digital Content 2, Supplementary Table 2, https://links.lww.com/AA/F425).

An additional analysis was performed in which we explored the effects of MAP on BIS and the effects of MAP and ephedrine on BIS. MAP was significantly associated with BIS, in which per 10 mm Hg increase in MAP, the BIS increased 2.1 points (CI, 1.2–3.1; *P* < .001). When both MAP and ephedrine were added to the mixed effects model analysis, both were significantly associated with BIS. For every 10 mm Hg increase in MAP, the BIS increased by 1.7 points (CI, 0.8–2.5; *P* < .001), and the BIS was 4.17 (CI, 2.52–5.82) points higher for the measurements taken after ephedrine administration (*P* < .001).

### CMAP 5–1 Ratios

CMAP 5–1 ratios remained stable over time during the study measurements. Therefore, in all patients, per muscle (TA left and right, AH left and right), no late effects of muscle relaxants were observed throughout the study measurements. All CMAP5-1 ratios per patient, per muscle, and per measurement can be found in Supplemental Digital Content 3, Supplementary Material 2, https://links.lww.com/AA/F426.

## DISCUSSION

This study investigated the effects of vasopressor-induced arterial blood pressure elevation on Tc-mMEP measurements. Significant associations between MAP and Tc-mMEP amplitude (*P* < .02), AUC (*P* < .01), and voltage threshold (*P* = .018) were found (Figure 1). When BIS and estimated plasma propofol concentration were added to the mixed effects model analysis, MAP remained significantly associated only with the Tc-mMEP amplitude and AUC of the AH muscles. The associations between MAP and Tc-mMEP voltage threshold were no longer statistically significant.

The significant association between MAP and the Tc-mMEP amplitude and AUC of the AH muscles remained after adjusting for ephedrine. The influence of cardiac index on Tc-mMEP amplitude and AUC provided similar results when compared to the influence of MAP on Tc-mMEP amplitude and AUC. However, there was no significant association anymore between CI and Tc-mMEP when corrected for ephedrine.

The warning criteria, considering the amplitude reduction method, used for the type of patients included in this study usually vary between 50% and 80% amplitude decrease. It is likely that intraoperative declines in MAP may contribute to the high rate of false positive warnings observed in clinical practice, as we found that a 10 mm Hg increase in MAP was associated with a 11.0% to 17.7% increase in the Tc-mMEP amplitude.^2,7^ The voltage threshold at MAP 60 mm Hg was significantly higher compared to that at MAP 100 mm Hg. The difference was only −5.51 V (CI, −10.05 to −0.97), which is probably not clinically relevant because with the threshold-level method, the threshold for a warning criterion is an increase in threshold by 100 volts or more. Moreover, when corrected for BIS and measured plasma propofol concentration, the association between MAP and voltage threshold was not significant anymore. Therefore, monitoring with the threshold level method could reduce the frequency of false positive warnings induced by MAP changes.

MAPs between 60 and 160 mm Hg are considered within the range in which cerebral and spinal cord autoregulation occurs.^26^ Therefore, one could argue that increasing the MAP from 60 to 100 mm Hg should not influence Tc-mMEP measurements, since elevations of MAP in this range should not have caused changes in cerebral or spinal cord perfusion.^27^ However, Meng et al summarized theoretical arguments and also existing literature indicating that cardiac output and hence also cardiac index, influence the cerebral blood flow.^26^ Therefore, the increase in cardiac index observed in this study is likely to have increased cerebral blood flow. This may have increased cortical excitability by increasing cortical oxygen delivery and might therefore be an explanation for our significantly higher Tc-mMEP amplitudes and AUCs within the cerebral and spinal cord autoregulation range. Moreover, our patients did not have other risk factors that might have increased the lower limit of autoregulation, such as high age and preexistent hypertension^28^ and possible effects of hemorrhage and thereby increasing propofol plasma concentrations were also not applicable to this data.^13^

The physiological mechanism by which pharmacological elevation of the MAP with norepinephrine affects Tc-mMEPs is unclear. Our findings might be explained by direct neuroexcitatory effects of norepinephrine.^29^ Multiple pharmacological studies have investigated the effects of norepinephrine agonists and reuptake inhibitors on MEPs elicited by transcranial magnetic stimulation.^30–33^ They all suggested that these effects had a cortical origin since the α-motor neuron and neuromuscular excitability remained stable, as there were no significant differences in H-reflex and CMAP amplitudes. However, whereas these investigators administered agents that cross the blood-brain barrier, in our study, we administered norepinephrine by peripheral venous infusion. This is important since it is claimed that catecholamines do not penetrate the blood-brain barrier.^34^ If the latter is true, then our results cannot be explained by increased cortical excitability caused by direct effects of norepinephrine.

An alternative explanation might be that norepinephrine has excitatory effects on the α-motor neuron.^29^ However, if the increase in the Tc-mMEP amplitude is explained by increased excitability of the peripheral nervous system, then we would have expected an increased amplitude of the H-reflex and/or CMAP, which we did not see in our results (Figure 1).

Ephedrine is known to penetrate the blood-brain barrier, and as seen in our data, to increase the BIS significantly.^34,35^ Ishiyama et al speculated that possible mechanisms for ephedrine’s effect on BIS could include indirect activation of α1 adrenergic and dopamine receptors in the CNS, and accelerated distribution of propofol resulting in reduced effect-site propofol concentrations. We consider the latter unlikely as propofol concentrations remained stable during our study.

In our previously published retrospective study, in which the association of physiological and pharmacological anesthetic parameters with motor evoked potentials was investigated using a multivariable longitudinal mixed model analysis, both MAP and BIS were significantly associated with Tc-mMEP amplitude.^36^ However, in this prospective study, the association between Tc-mMEP characteristics and MAP is largely attenuated when adjusting for BIS, plasma propofol concentration, and ephedrine. In the post hoc analyses, we showed that, when corrected for estimated plasma propofol concentration, there was a significant association between MAP and BIS. A possible explanation might be that increasing MAP with norepinephrine enhances cortical excitability, as reflected by the increase in BIS. These additional results support the idea that cortical excitability was influenced by the pressure regimen and might mediate the changes in Tc-mMEP amplitude and AUC. However, it remains unclear if this is an effect of norepinephrine or blood pressure.

A study published by Baust et al in 1963 suggests an explanation for our findings.^37^ They investigated the effect of blood pressure elevation, either by adrenaline or mechanically induced, on EEG in cats using an “encéphale isolé” preparation. They concluded that not adrenaline, but blood pressure elevation itself increased cortical arousal, measured by EEG, suggesting a pressure-sensitive structure within the posterior hypothalamus and mesencephalic reticular formation causing the increased arousal after blood pressure elevation. Taken together, these findings suggest that MAP elevation alone might influence cortical excitability and thereby increase Tc-mMEP amplitude and AUC.

### Future Perspective

To provide further insights, support and verify the results of Baust et al in humans, a future investigation of the effects of MAP elevation with phenylephrine, rather than, or compared with noradrenaline or ephedrine on Tc-mMEPs could help to demonstrate the direct effect of MAP on the cortical excitability, since phenylephrine administration has been shown not to increase BIS values.^35^

The effects of vasopressor-induced MAP elevation on direct-waves (D-waves) might also help understand the underlying physiology of our findings. D-waves are elicited by transcranial electrical stimulation and can be recorded in the subdural or epidural space of the spinal cord.^2^ If the cortical excitability increases because of vasopressor-induced MAP elevation, then the D-wave amplitude should also increase (Figure 1). Since D-waves are measured at the spinal cord, they are not susceptible to possible neuroexcitatory effects of norepinephrine on α-motor neurons.

### Limitations

First, the cardiac output data were measured directly or calibrated. Instead, the system used for these measurements estimated cardiac output based on pulse contour analysis. Secondly, ephedrine was administered at the end of the study measurements to achieve an MAP of 100 mm Hg. This could have created bias in BIS measures and Tc-mMEP characteristics since ephedrine was not given to achieve the lower MAP target value. Moreover, as described before, ephedrine can penetrate the blood-brain barrier and has been shown to increase the BIS significantly.^34,35^ In hindsight, phenylephrine would have been a more appropriate choice, as it does not increase BIS.^35^ Moreover, ephedrine administration was included in our models as a binary variable (scored as “yes” or “no”). Thereby, we did not consider the dose administered, or the time between administration and Tc-mMEP measurement. Lastly, for ethical and logistical reasons, we did not use methods to assess spinal cord or cerebral perfusion, which could have helped to understand the physiology behind our findings.

## CONCLUSIONS

When the MAP was pharmacologically elevated with a norepinephrine infusion, significant and clinically relevant associations between MAP and Tc-mMEP amplitude and AUC were found. When corrected for BIS, plasma propofol concentration, and use of ephedrine, the associations between increasing MAP and Tc-mMEP characteristics were largely attenuated. BIS values were significantly increased by increasing MAP, when corrected for estimated plasma propofol concentration. These results support the idea that cortical excitability was influenced by the pressure regimen and might mediate the changes in Tc-mMEP amplitude and AUC. Thereby, our results imply that increasing MAP increases the cortical excitability. Further research is necessary to better understand the underlying physiology of our findings.

## ACKNOWLEDGMENTS

We acknowledge anesthesiologists: J. P. Valk, MD, M. A. Schoorl, MD, A. J. M. Konijn, MD, and A. M. Venema, MD, PhD; neurosurgeons: J. R. Jeltema, MD, G. Rijtema, MD, and J. D. M. Metzemaekers, MD, PhD; and neurophysiology technician: Mrs C. H. M. Scholtens-Henzen.

## DISCLOSURES

**Conflicts of Interest:** A. R. Absalom reports receipt of unrestricted research funding and/or reimbursement for consultancy from Philips (all payments to institution), and is a trustee of the *British Journal of Anaesthesia* company. No other authors declared Conflicts of Interest. **Funding:** None. **This manuscript was handled by:** Markus W. Hollmann, MD, PhD.

## Supplementary Material


