# Supplementary material for: The Influence of Vasopressor-Induced Arterial Blood Pressure Elevation on Muscle-Recorded Motor Evoked Potentials
Source: Anesth Analg. 2025 Sep 5;142(4):730–40. doi: 10.1213/ANE.0000000000007701 (PMC12959597; doi:10.1213/ANE.0000000000007701)
Supplement: Supplementary file 1 [file ane-142-730-s001.pdf]

Supplementary material 1

In supplementary material 1, the individual plots of the time course of anaesthetic variables, and amplitudes and AUCs are shown per variable per patient.

If a graph is blank or if there is an interruption in a drawn line, there was missing data.

Patient 1

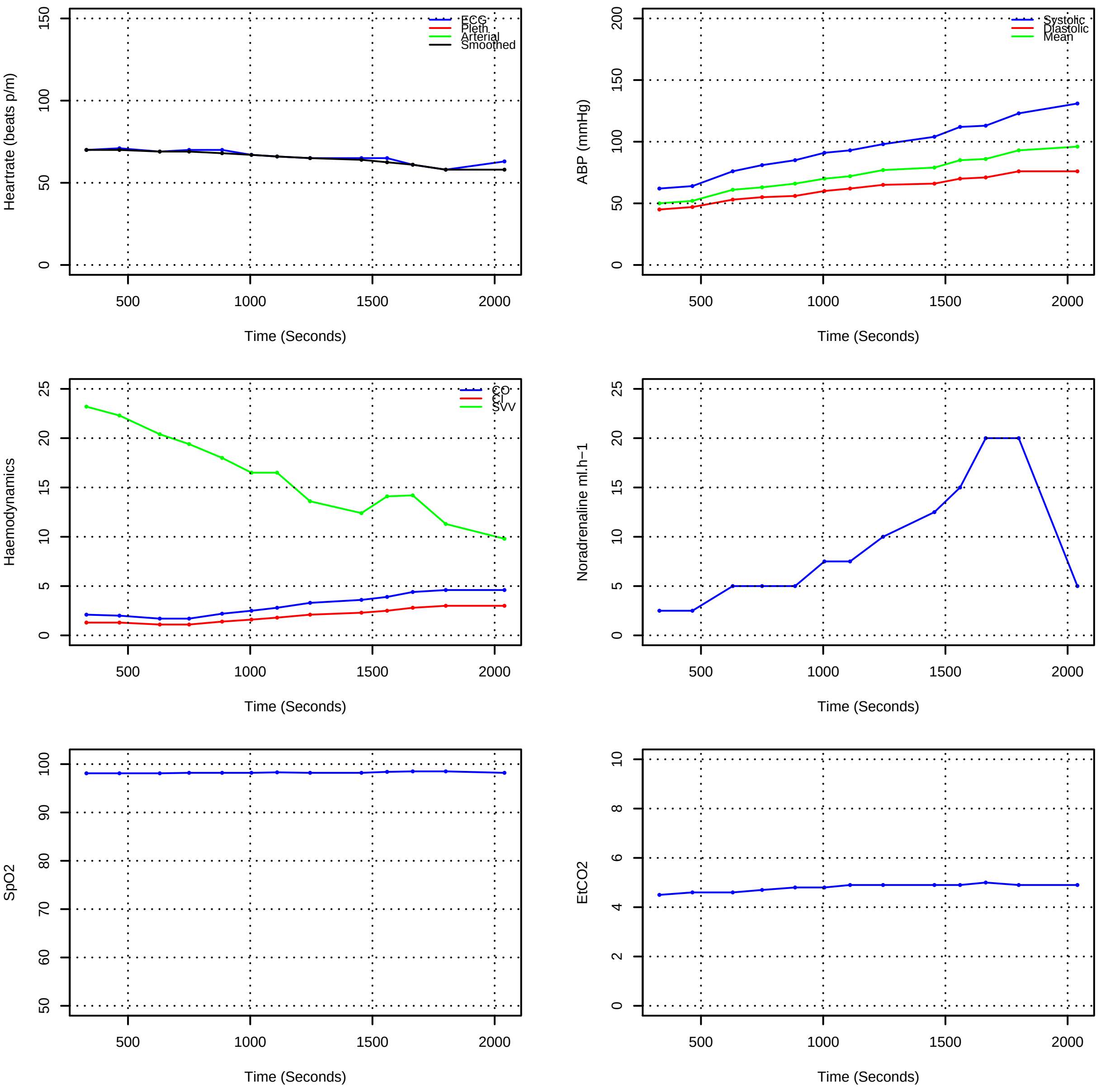

Heartrate (beats p/m)
ECG
Pleth
Arterial
Smoothed
ABP (mmHg)
Systolic
Diastolic
Mean
Haemodynamics
CO
CI
SVV
Noradrenaline ml.h−1
SpO2
EtCO2
Time (Seconds)

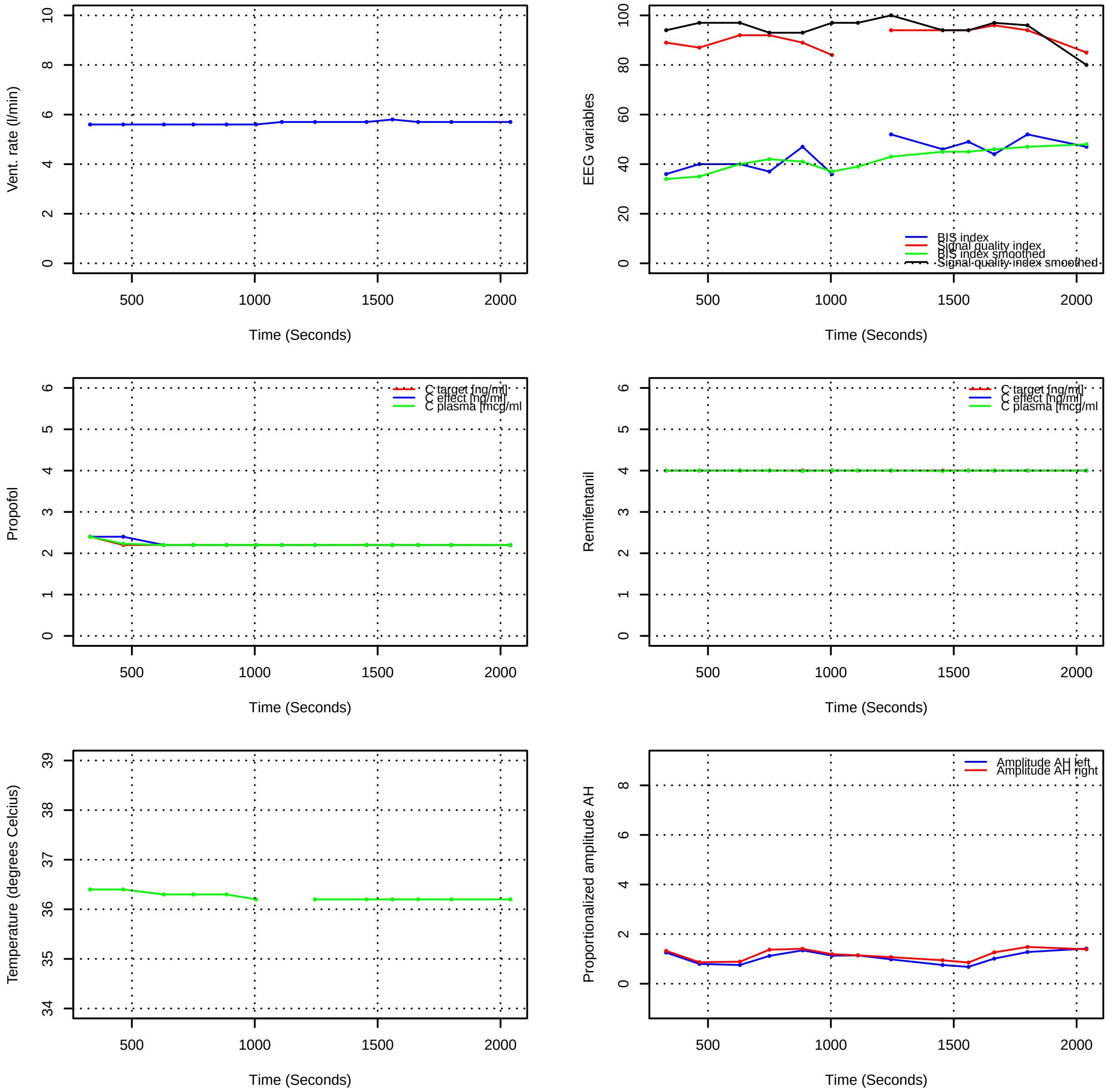
Vent. rate (l/min)
Time (Seconds)
EEG variables
BIS index
Signal quality index
BIS index smoothed
Signal quality index smoothed
Time (Seconds)
Propofol
C target [ng/ml]
C effect [ng/ml]
C plasma [mcg/ml
Time (Seconds)
Remifentanil
C target [ng/ml]
C effect [ng/ml]
C plasma [mcg/ml
Time (Seconds)
Temperature (degrees Celcius)
Time (Seconds)
Proportionalized amplitude AH
Amplitude AH left
Amplitude AH right
Time (Seconds)

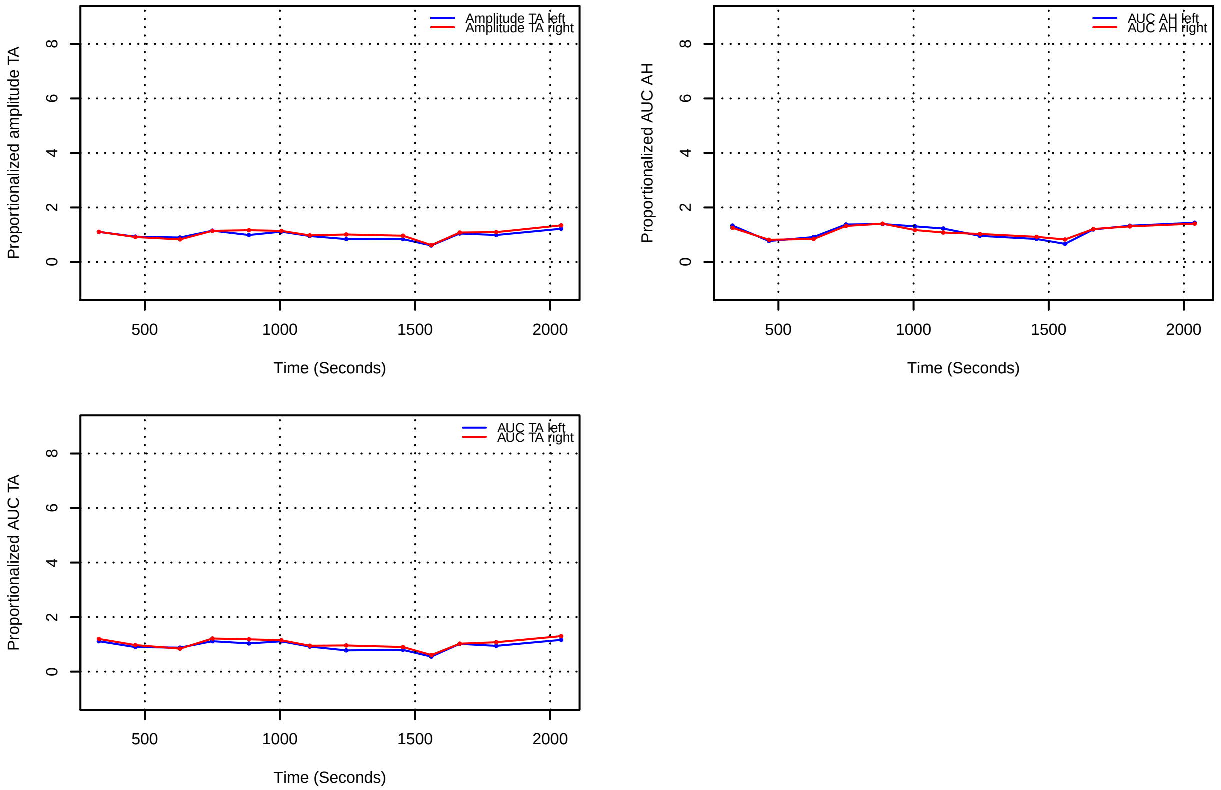

Amplitude TA left
Amplitude TA right
Proportionalized amplitude TA
Time (Seconds)
AUC AH left
AUC AH right
Proportionalized AUC AH
Time (Seconds)
AUC TA left
AUC TA right
Proportionalized AUC TA
Time (Seconds)

Patient 2

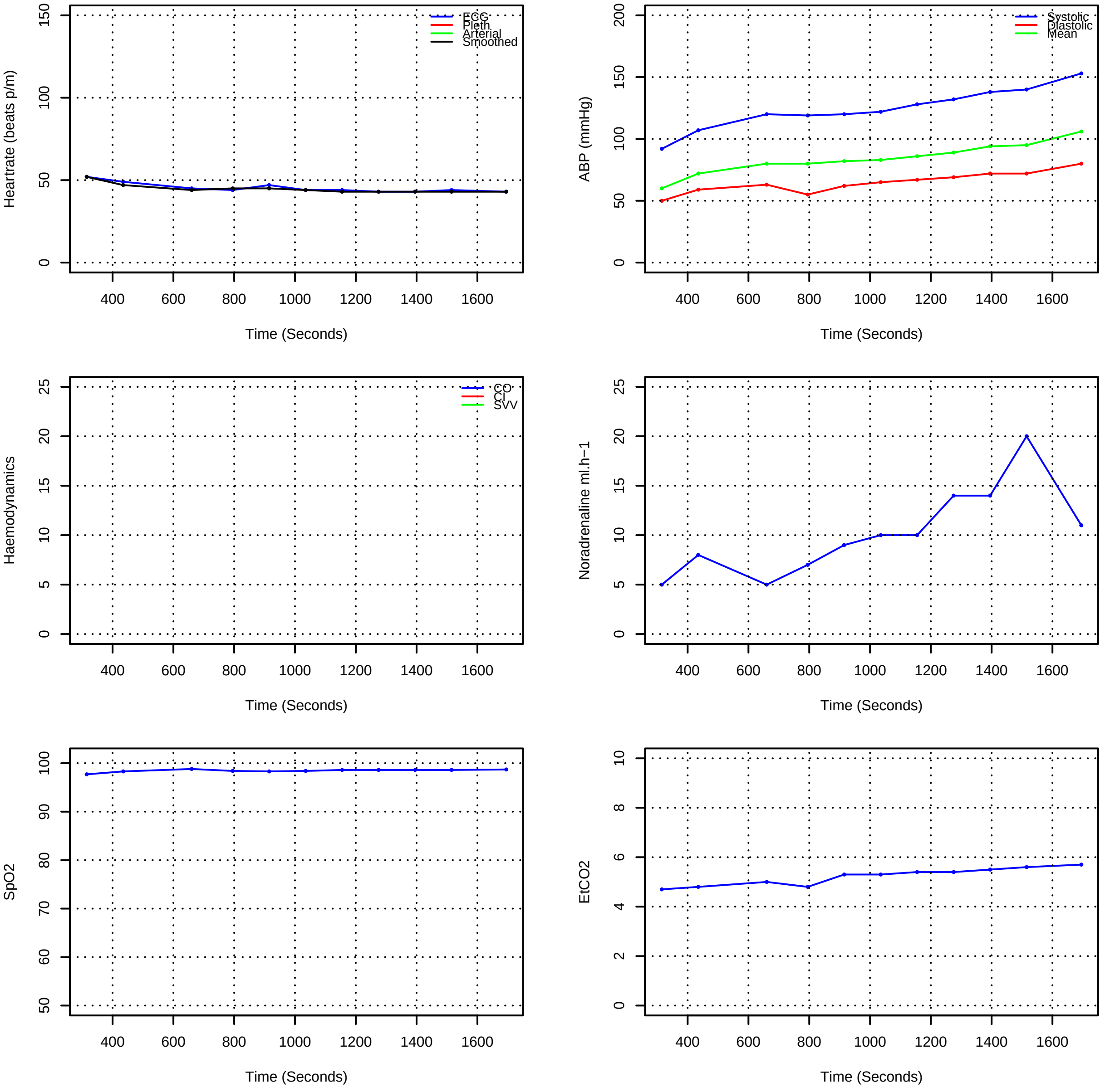

Heartrate (beats p/m)
ECG
Pleth
Arterial
Smoothed
ABP (mmHg)
Systolic
Diastolic
Mean
Haemodynamics
CO
CI
SVV
Noradrenaline ml.h−1
SpO2
EtCO2
Time (Seconds)

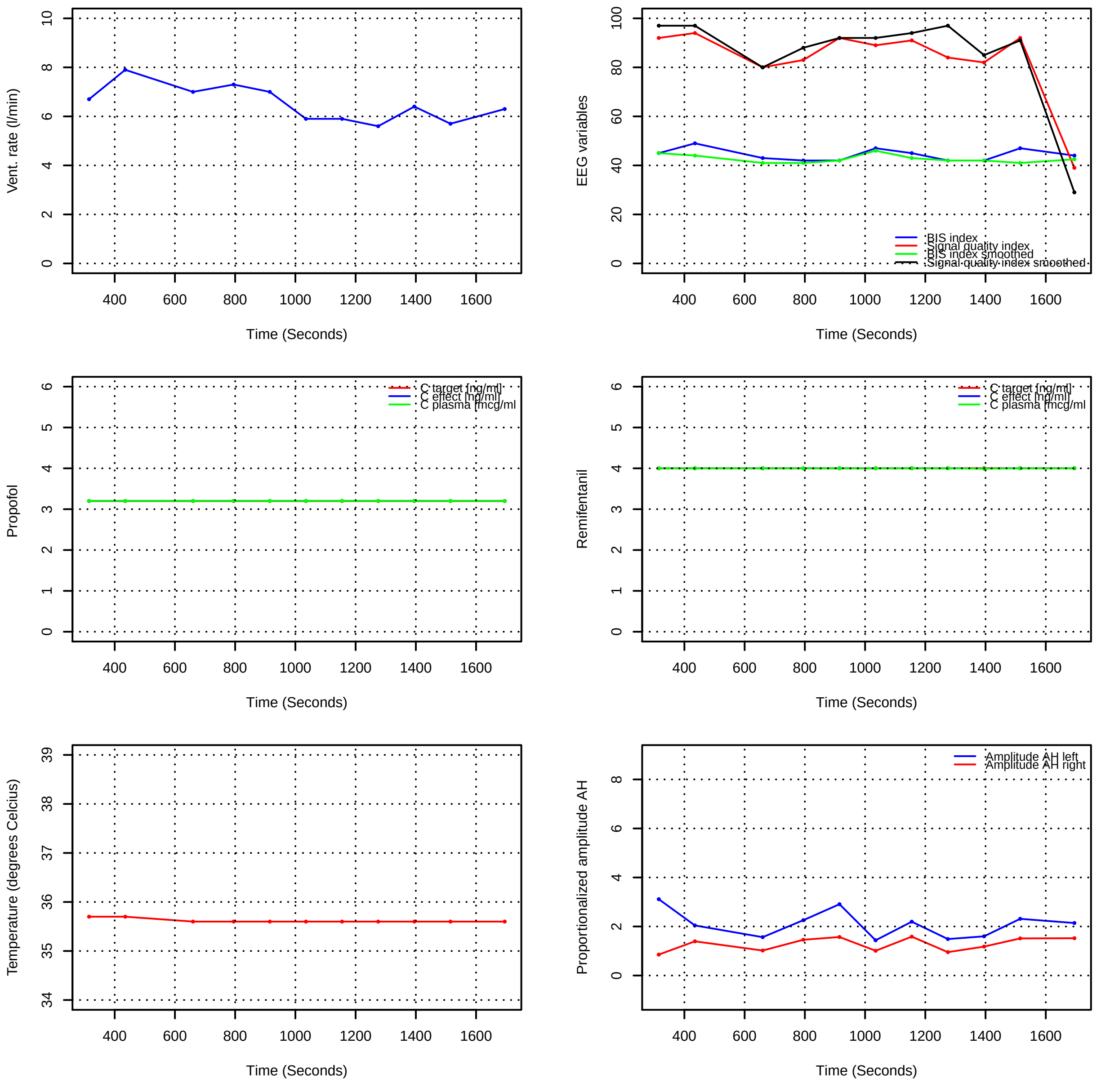
Vent. rate (l/min)
Time (Seconds)
EEG variables
BIS index
Signal quality index
BIS index smoothed
Signal quality index smoothed
Time (Seconds)
Propofol
C target [ng/ml]
C effect [ng/ml]
C plasma [mcg/ml
Time (Seconds)
Remifentanil
C target [ng/ml]
C effect [ng/ml]
C plasma [mcg/ml
Time (Seconds)
Temperature (degrees Celcius)
Time (Seconds)
Proportionalized amplitude AH
Amplitude AH left
Amplitude AH right
Time (Seconds)

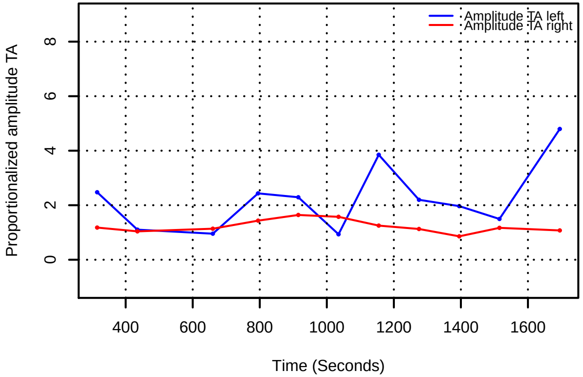
Amplitude TA left
Amplitude TA right
Proportionalized amplitude TA
0
2
4
6
8
400
600
800
1000
1200
1400
1600
Time (Seconds)

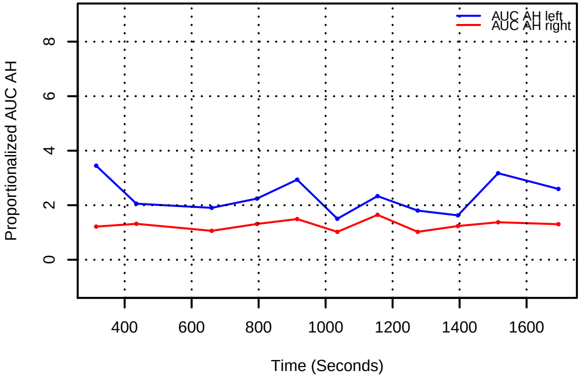
AUC AH left
AUC AH right
Proportionalized AUC AH
0
2
4
6
8
400
600
800
1000
1200
1400
1600
Time (Seconds)

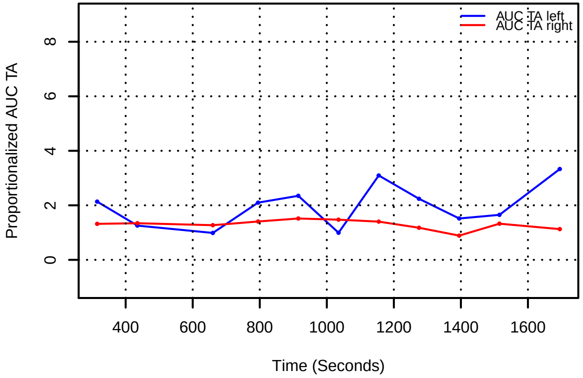
AUC TA left
AUC TA right
Proportionalized AUC TA
0
2
4
6
8
400
600
800
1000
1200
1400
1600
Time (Seconds)

Patient 3

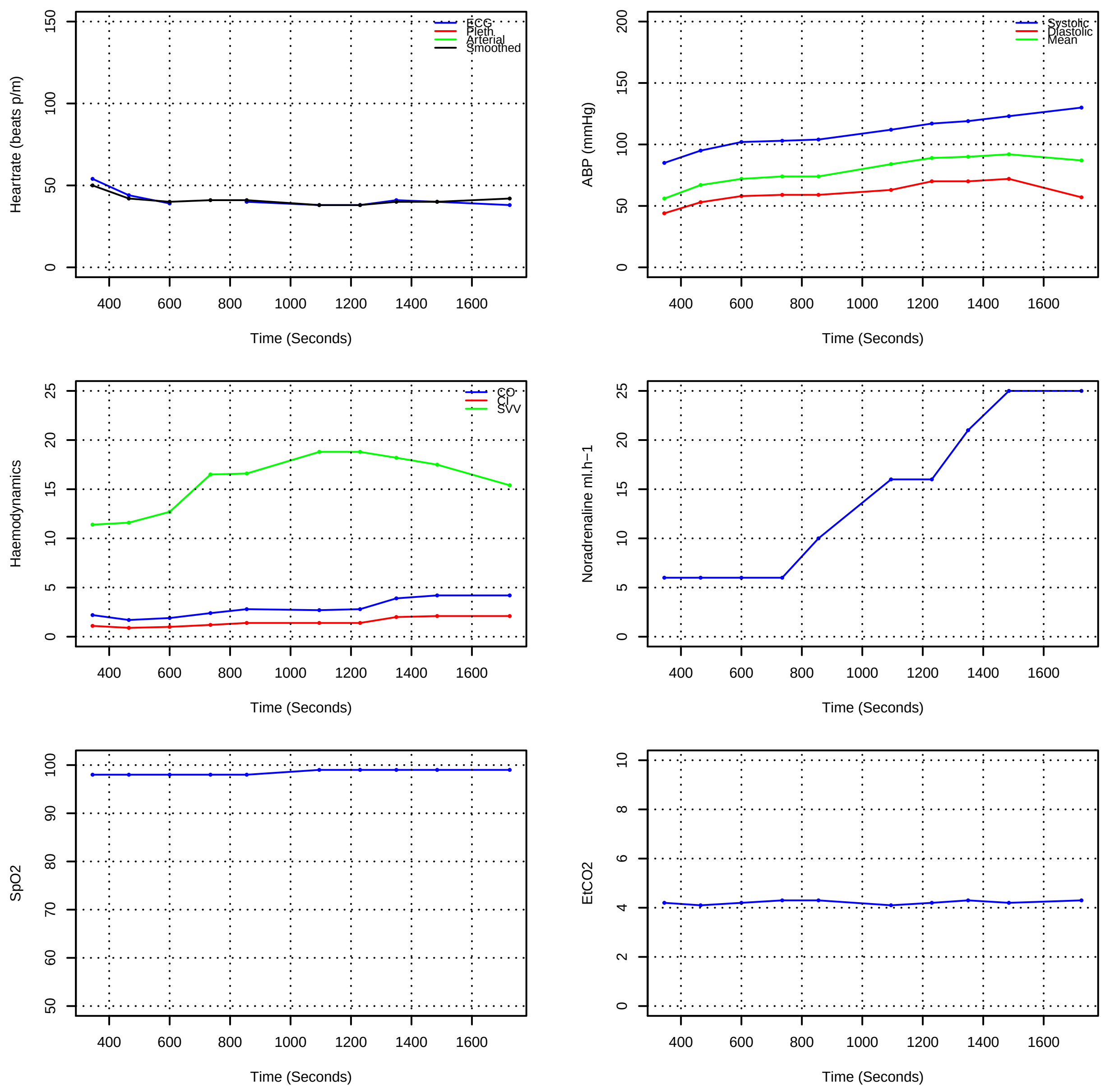

Heartrate (beats p/m)
ECG
Pleth
Arterial
Smoothed
ABP (mmHg)
Systolic
Diastolic
Mean
Haemodynamics
CO
CI
SVV
Noradrenaline ml.h−1
SpO2
EtCO2
Time (Seconds)

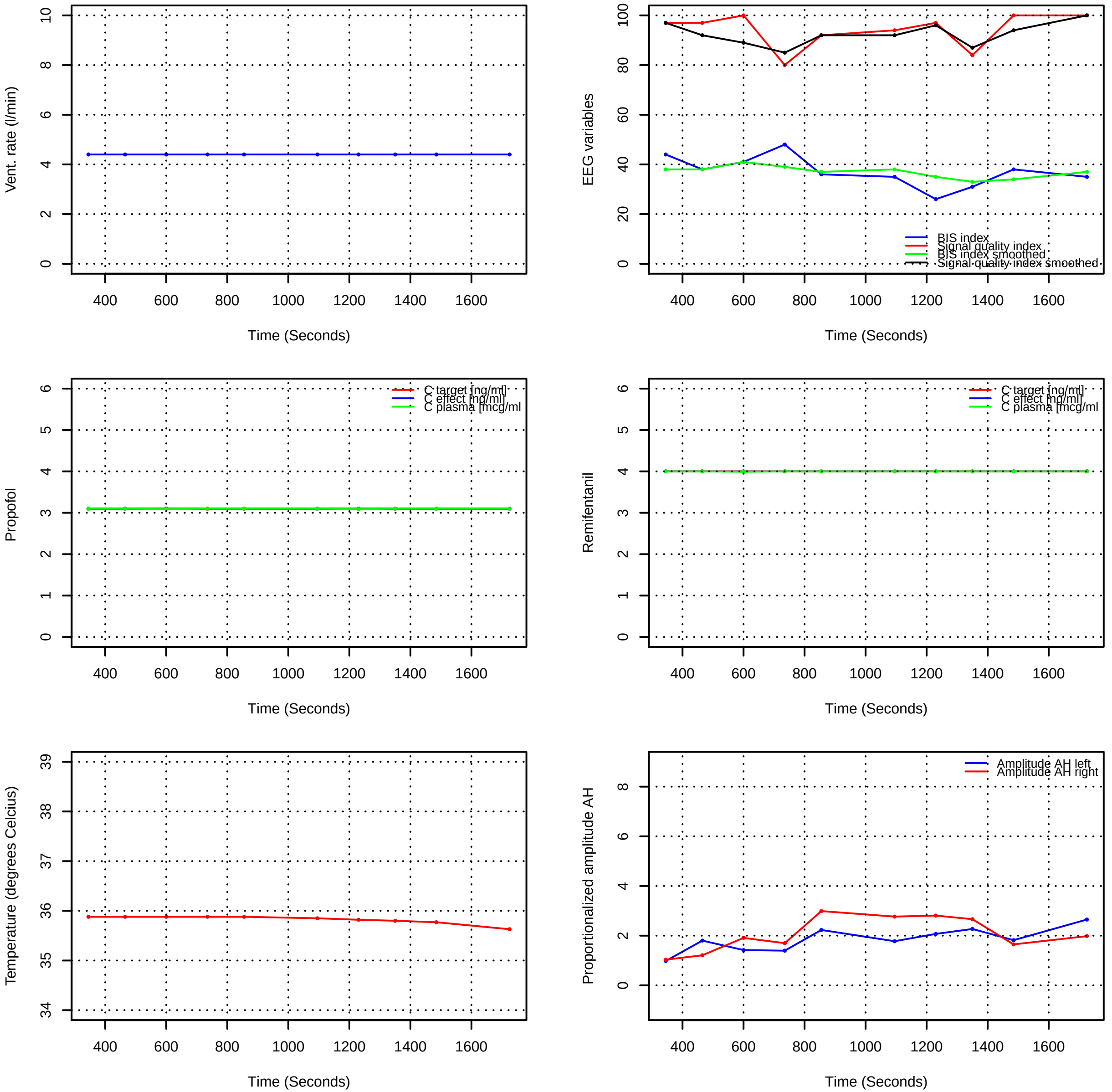

Vent. rate (l/min)
Time (Seconds)
EEG variables
BIS index
Signal quality index
BIS index smoothed
Signal quality index smoothed
Time (Seconds)
Propofol
C target [ng/ml]
C effect [ng/ml]
C plasma [mcg/ml
Time (Seconds)
Remifentanil
C target [ng/ml]
C effect [ng/ml]
C plasma [mcg/ml
Time (Seconds)
Temperature (degrees Celcius)
Time (Seconds)
Proportionalized amplitude AH
Amplitude AH left
Amplitude AH right
Time (Seconds)

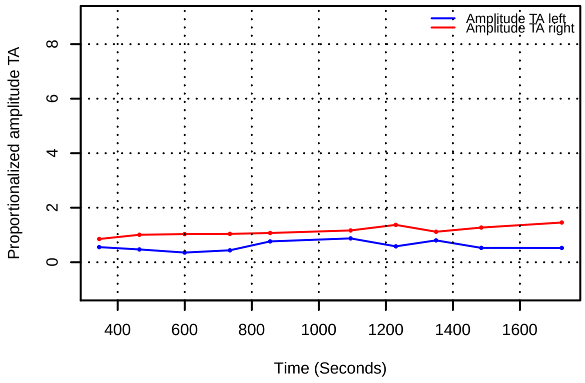
Amplitude TA left
Amplitude TA right
Proportionalized amplitude TA
Time (Seconds)
0
2
4
6
8
400
600
800
1000
1200
1400
1600

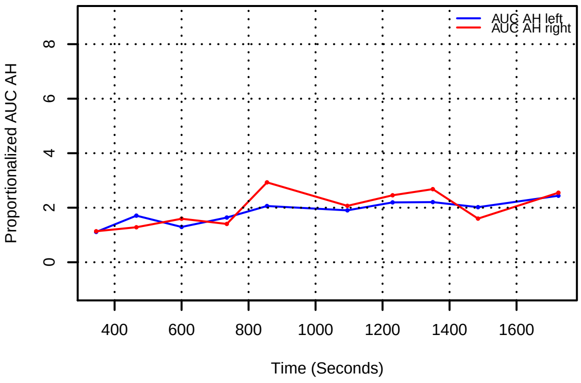
AUC AH left
AUC AH right
Proportionalized AUC AH
Time (Seconds)
0
2
4
6
8
400
600
800
1000
1200
1400
1600

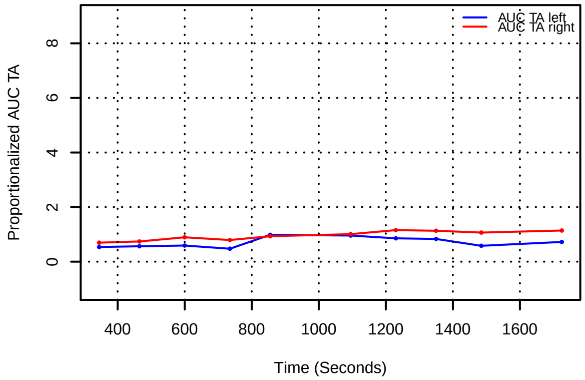
AUC TA left
AUC TA right
Proportionalized AUC TA
Time (Seconds)
0
2
4
6
8
400
600
800
1000
1200
1400
1600

Patient 4

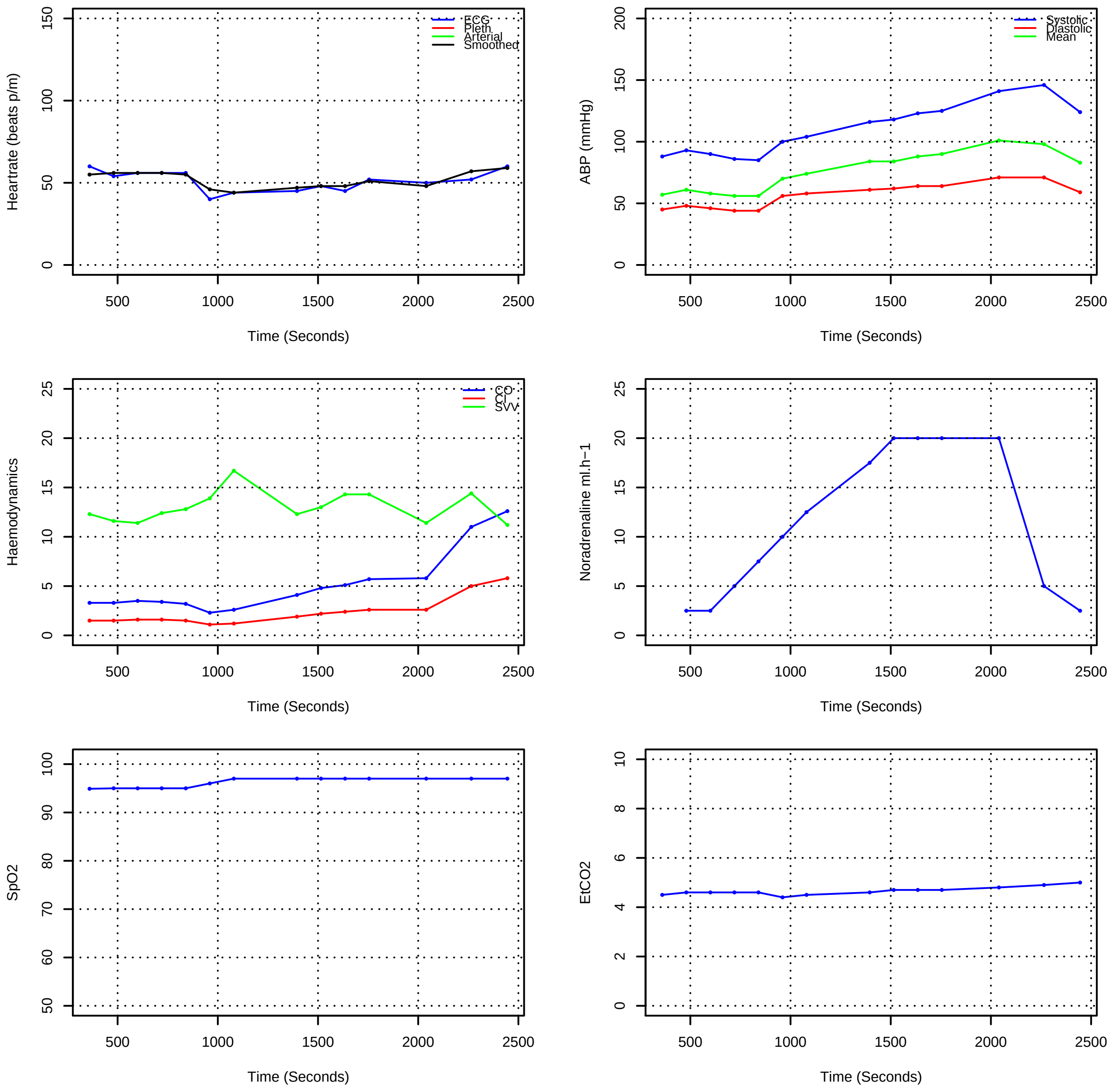
Heartrate (beats p/m)
ECG
Pleth
Arterial
Smoothed
ABP (mmHg)
Systolic
Diastolic
Mean
Haemodynamics
CO
CI
SVV
Noradrenaline ml.h−1
SpO2
EtCO2
Time (Seconds)

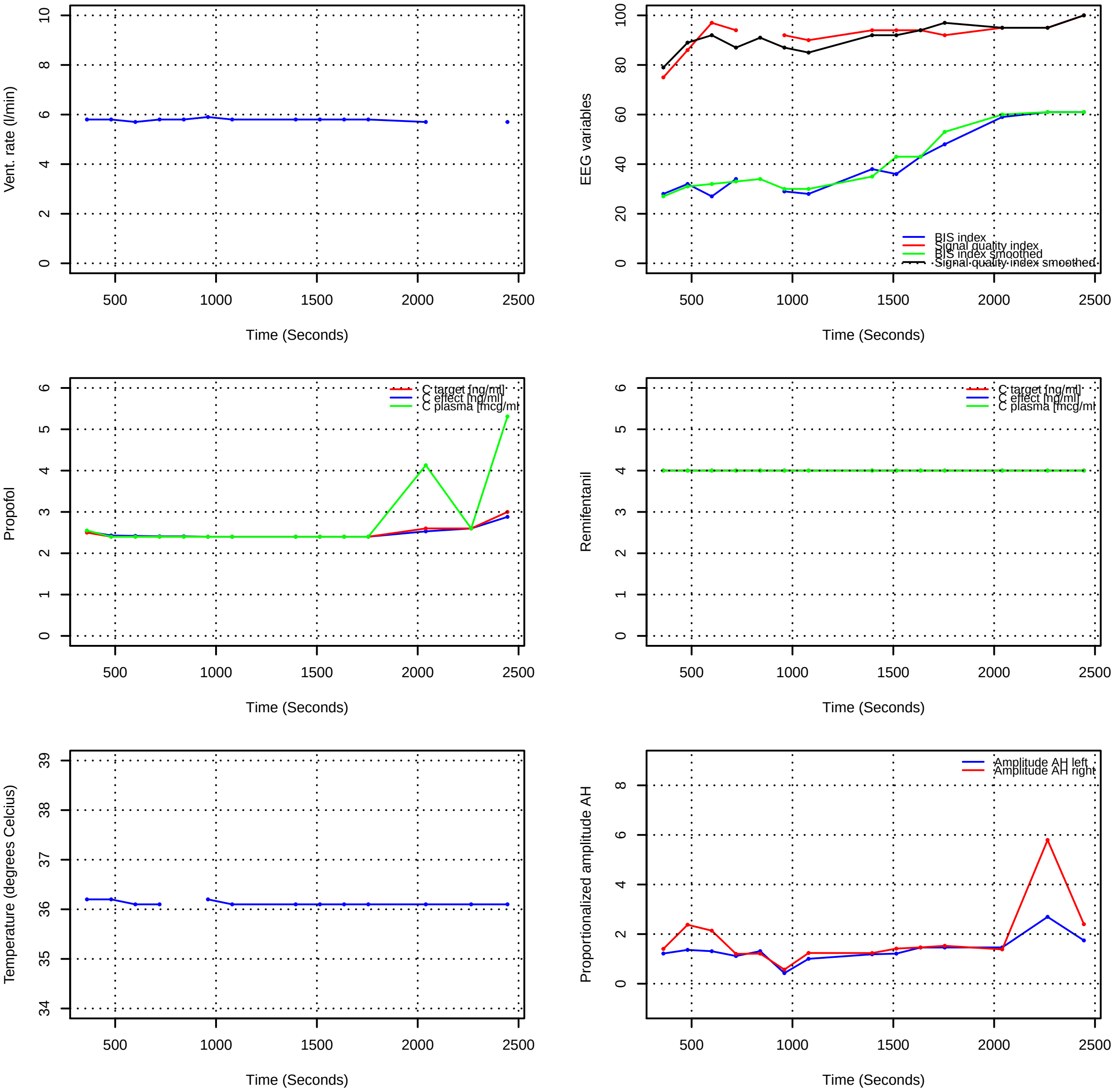
Vent. rate (l/min)
Time (Seconds)
EEG variables
BIS index
Signal quality index
BIS index smoothed
Signal quality index smoothed
Time (Seconds)
Propofol
C target [ng/ml]
C effect [ng/ml]
C plasma [mcg/ml
Time (Seconds)
Remifentanil
C target [ng/ml]
C effect [ng/ml]
C plasma [mcg/ml
Time (Seconds)
Temperature (degrees Celcius)
Time (Seconds)
Proportionalized amplitude AH
Amplitude AH left
Amplitude AH right
Time (Seconds)

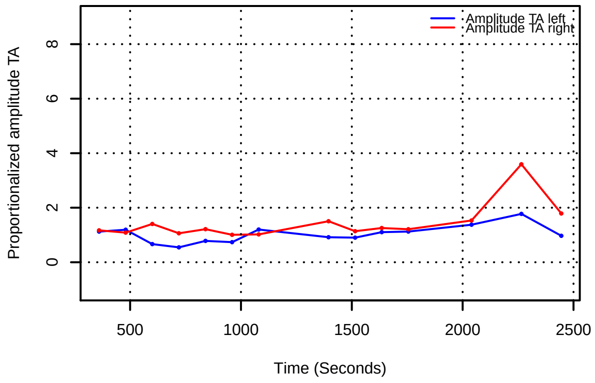

Amplitude TA left
Amplitude TA right
Proportionalized amplitude TA
0
2
4
6
8
500
1000
1500
2000
2500
Time (Seconds)

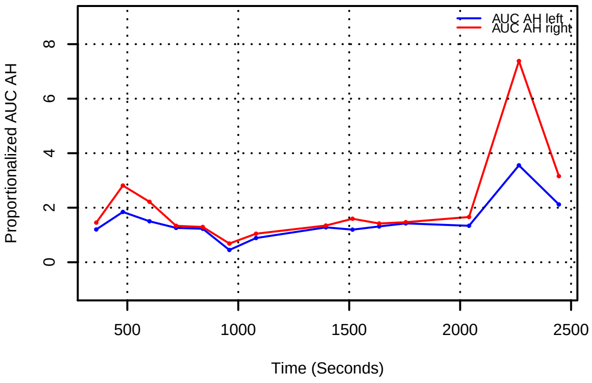

AUC AH left
AUC AH right
Proportionalized AUC AH
0
2
4
6
8
500
1000
1500
2000
2500
Time (Seconds)

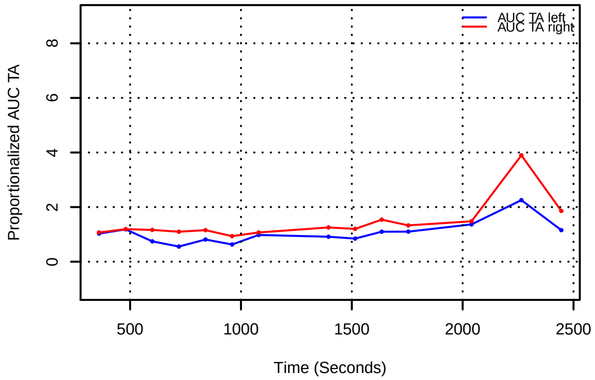

AUC TA left
AUC TA right
Proportionalized AUC TA
0
2
4
6
8
500
1000
1500
2000
2500
Time (Seconds)

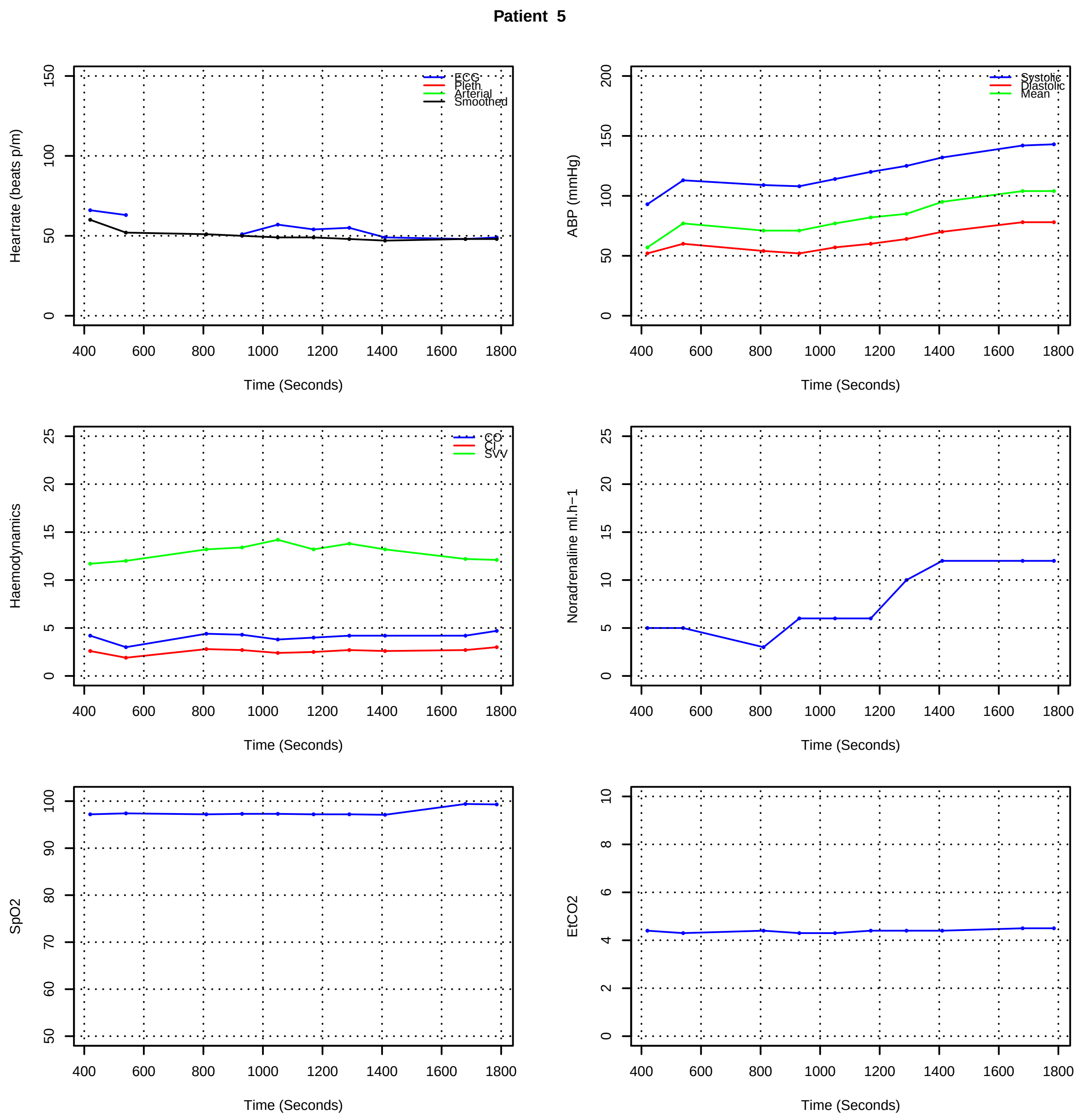
Patient 5
Heartrate (beats p/m)
ECG
Pleth
Arterial
Smoothed
ABP (mmHg)
Systolic
Diastolic
Mean
Haemodynamics
CO
CI
SVV
Noradrenaline ml.h−1
SpO2
EtCO2
Time (Seconds)

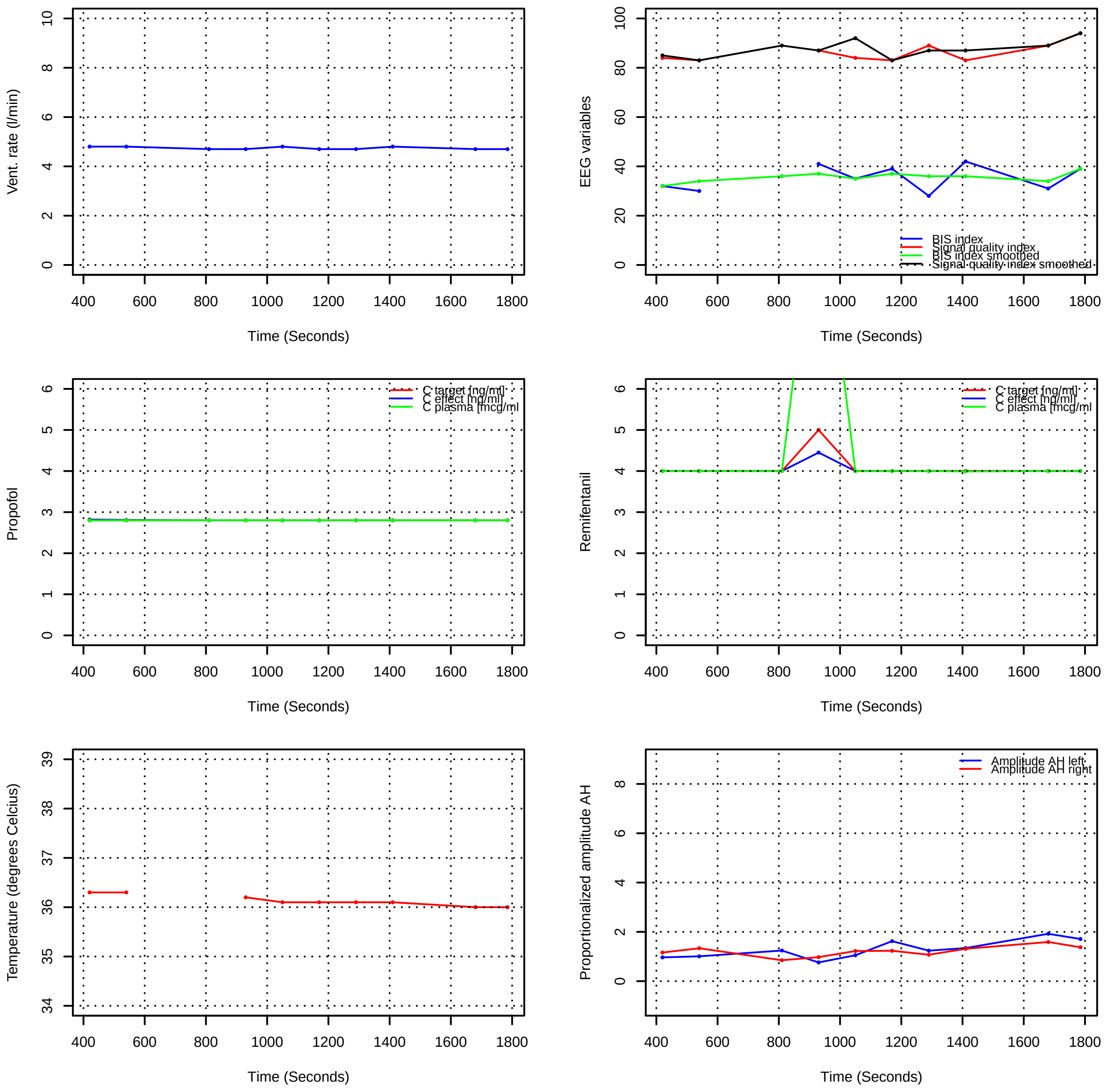

Vent. rate (l/min)
Time (Seconds)
EEG variables
BIS index
Signal quality index
BIS index smoothed
Signal quality index smoothed
Time (Seconds)
Propofol
C target [ng/ml]
C effect [ng/ml]
C plasma [mcg/ml
Time (Seconds)
Remifentanil
C target [ng/ml]
C effect [ng/ml]
C plasma [mcg/ml
Time (Seconds)
Temperature (degrees Celcius)
Time (Seconds)
Proportionalized amplitude AH
Amplitude AH left
Amplitude AH right
Time (Seconds)

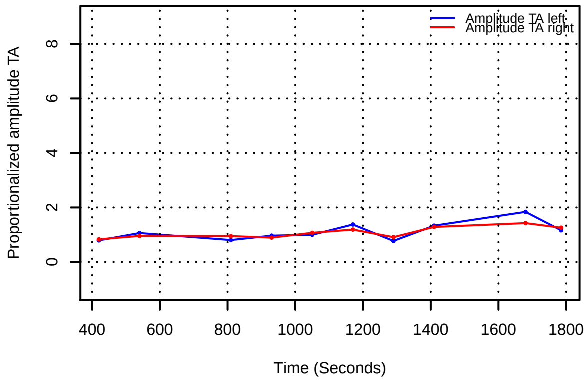
Amplitude TA left
Amplitude TA right
Proportionalized amplitude TA
0
2
4
6
8
400
600
800
1000
1200
1400
1600
1800
Time (Seconds)

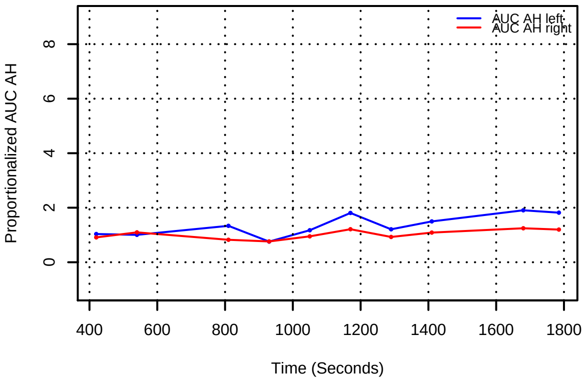
AUC AH left
AUC AH right
Proportionalized AUC AH
0
2
4
6
8
400
600
800
1000
1200
1400
1600
1800
Time (Seconds)

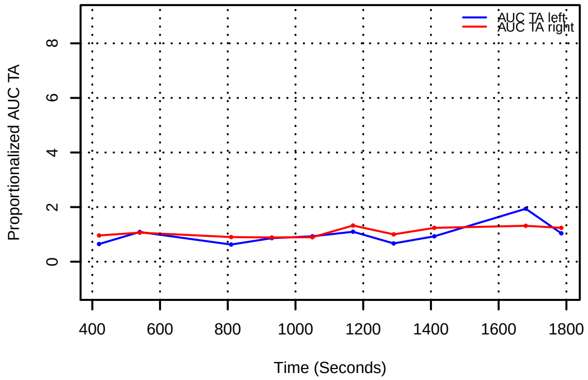
AUC TA left
AUC TA right
Proportionalized AUC TA
0
2
4
6
8
400
600
800
1000
1200
1400
1600
1800
Time (Seconds)

Patient 6

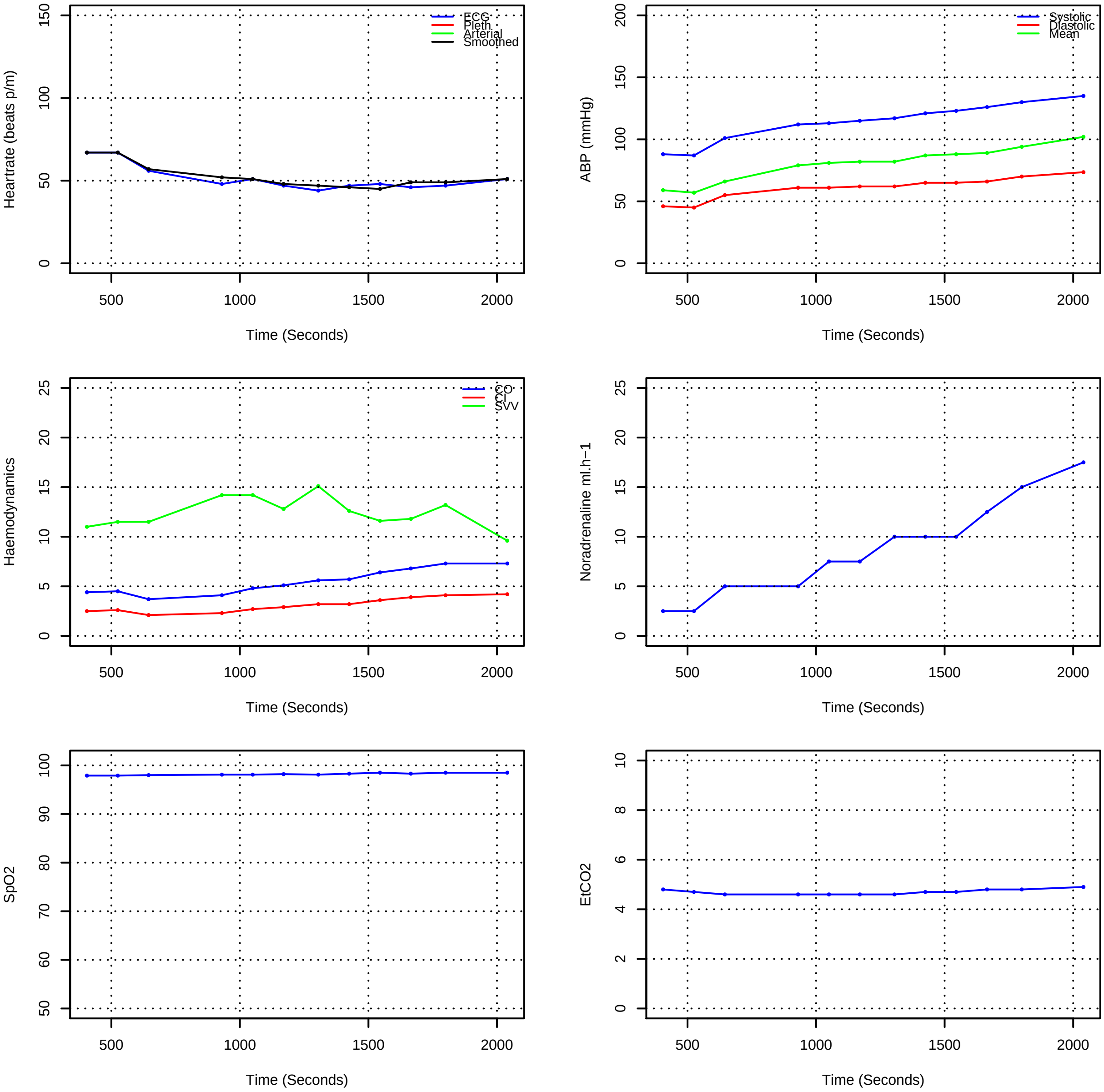

Heartrate (beats p/m)
ECG
Pleth
Arterial
Smoothed
Time (Seconds)
ABP (mmHg)
Systolic
Diastolic
Mean
Time (Seconds)
Haemodynamics
CO
CI
SVV
Time (Seconds)
Noradrenaline ml.h−1
Time (Seconds)
SpO2
Time (Seconds)
EtCO2
Time (Seconds)

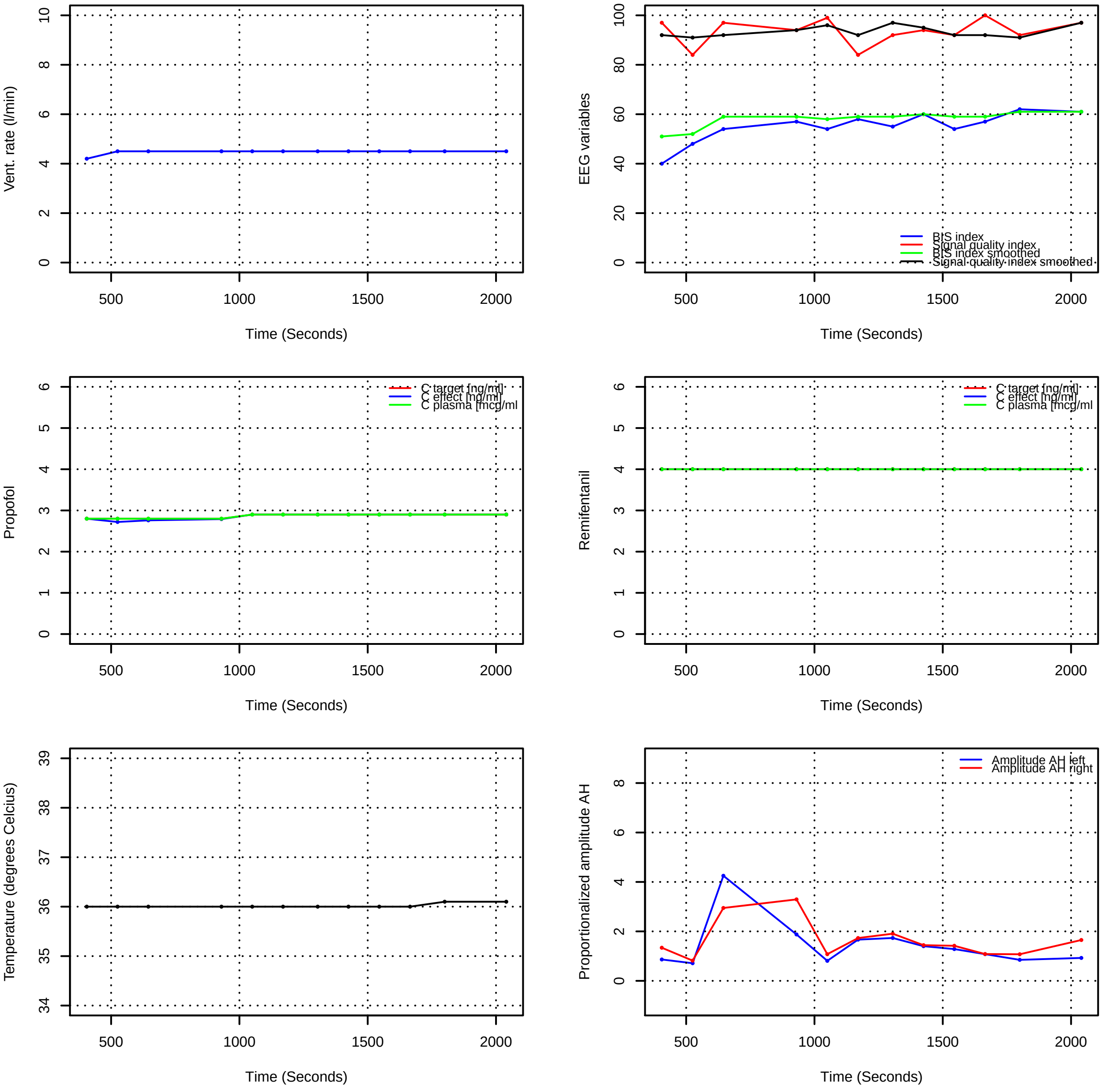

Vent. rate (l/min)
Time (Seconds)
EEG variables
BIS index
Signal quality index
BIS index smoothed
Signal quality index smoothed
Time (Seconds)
Propofol
C target [ng/ml]
C effect [ng/ml]
C plasma [mcg/ml
Time (Seconds)
Remifentanil
C target [ng/ml]
C effect [ng/ml]
C plasma [mcg/ml
Time (Seconds)
Temperature (degrees Celcius)
Time (Seconds)
Proportionalized amplitude AH
Amplitude AH left
Amplitude AH right
Time (Seconds)

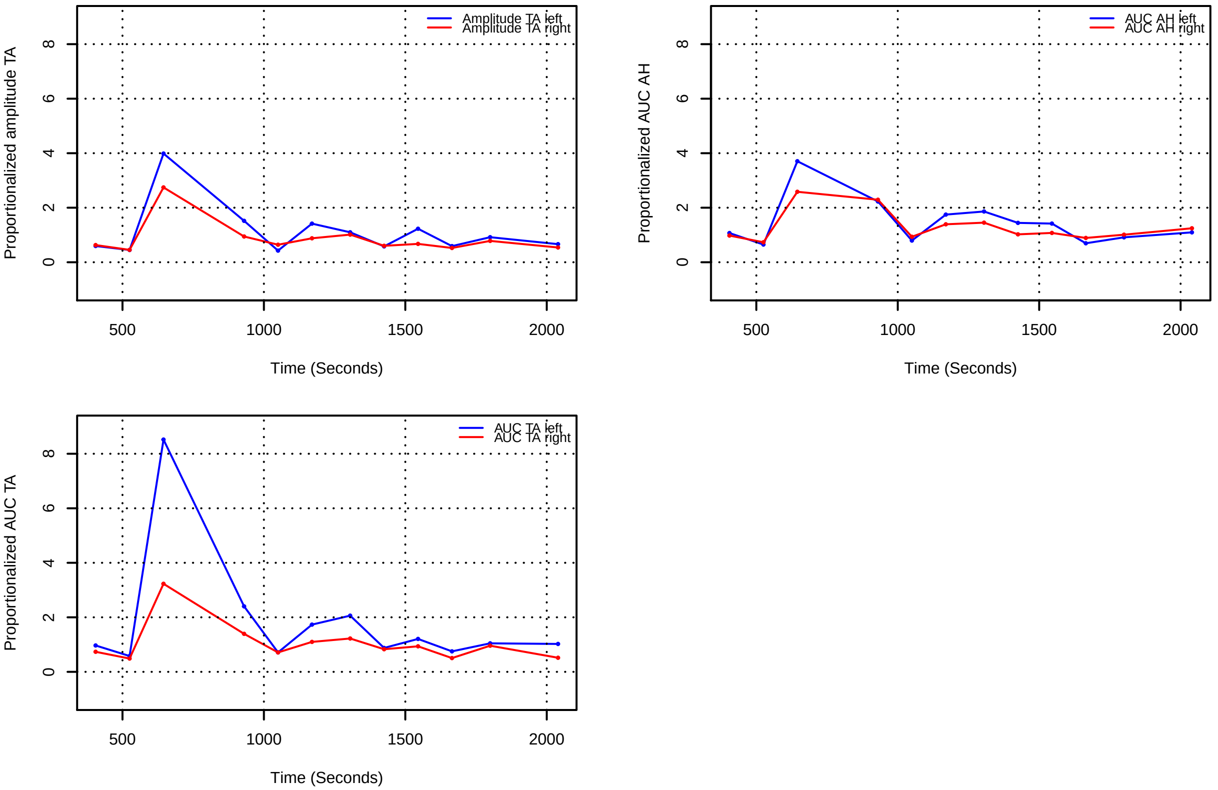
Amplitude TA left
Amplitude TA right
Proportionalized amplitude TA
Time (Seconds)
AUC AH left
AUC AH right
Proportionalized AUC AH
Time (Seconds)
AUC TA left
AUC TA right
Proportionalized AUC TA
Time (Seconds)

Patient 7

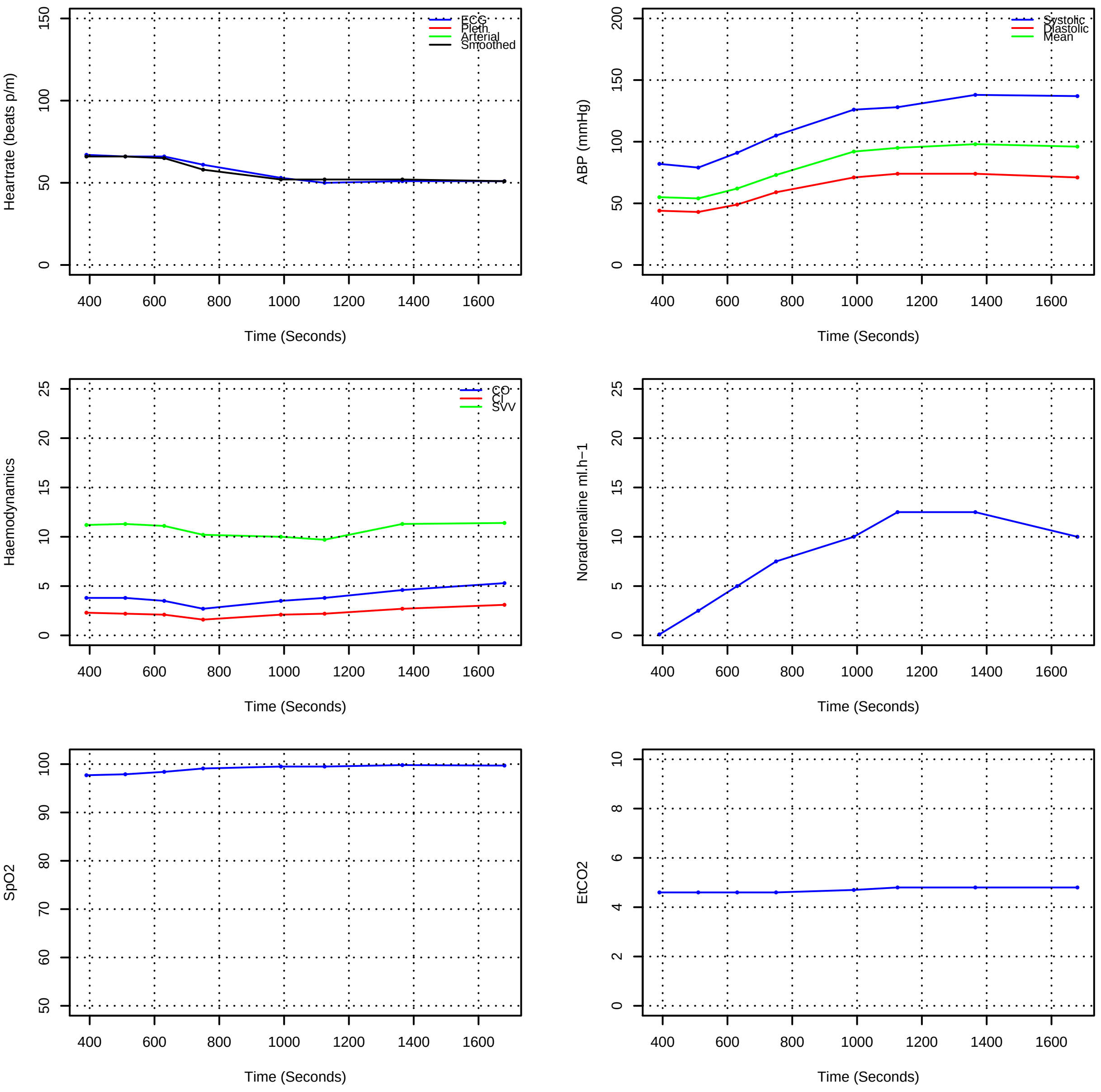

Heartrate (beats p/m)
ECG
Pleth
Arterial
Smoothed
Time (Seconds)
ABP (mmHg)
Systolic
Diastolic
Mean
Time (Seconds)
Haemodynamics
CO
CI
SVV
Time (Seconds)
Noradrenaline ml.h−1
Time (Seconds)
SpO2
Time (Seconds)
EtCO2
Time (Seconds)

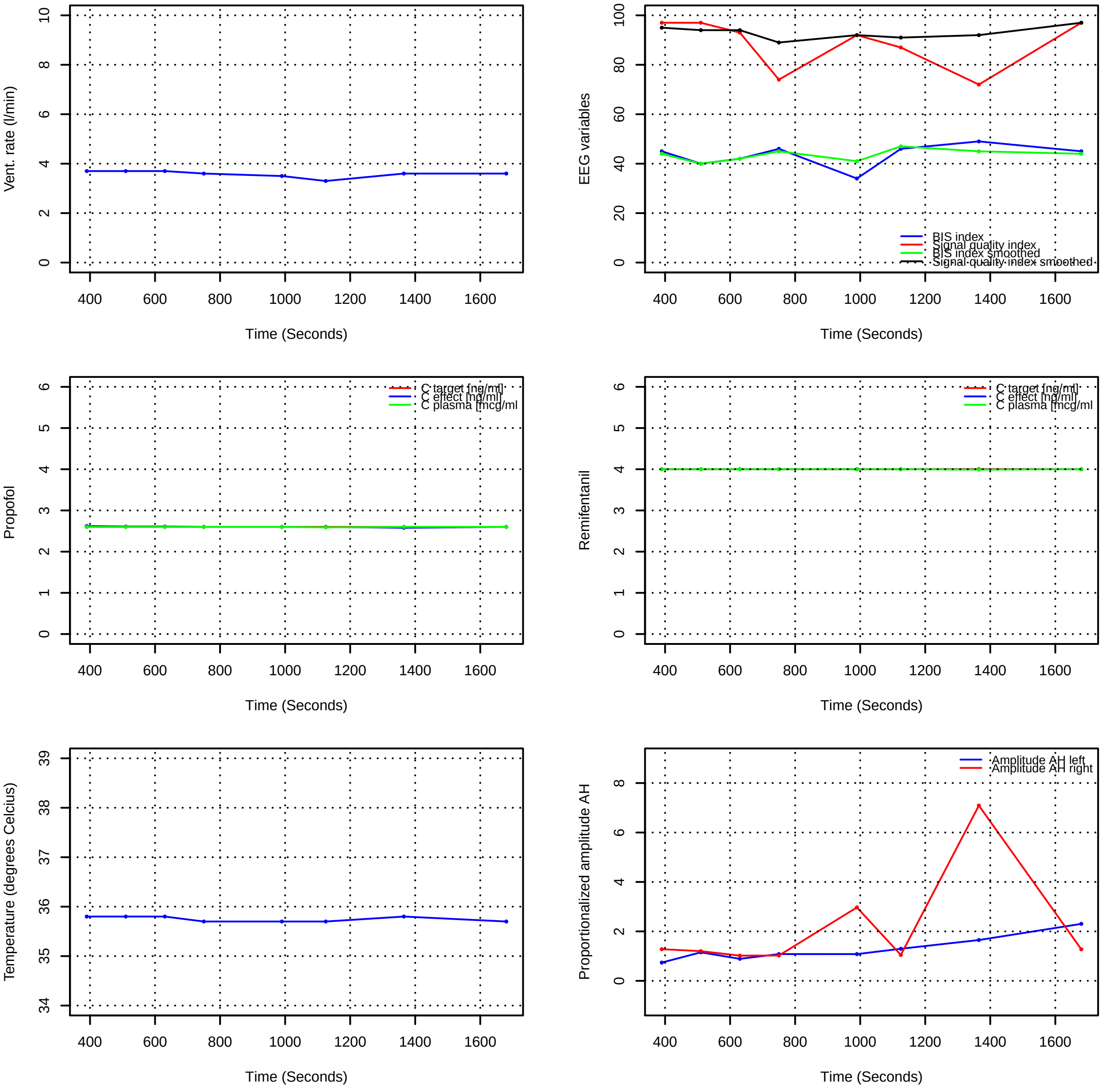
Vent. rate (l/min)
EEG variables
BIS index
Signal quality index
BIS index smoothed
Signal quality index smoothed
Propofol
Remifentanil
C target [ng/ml]
C effect [ng/ml]
C plasma [mcg/ml
Temperature (degrees Celcius)
Proportionalized amplitude AH
Amplitude AH left
Amplitude AH right
Time (Seconds)
400
600
800
1000
1200
1400
1600

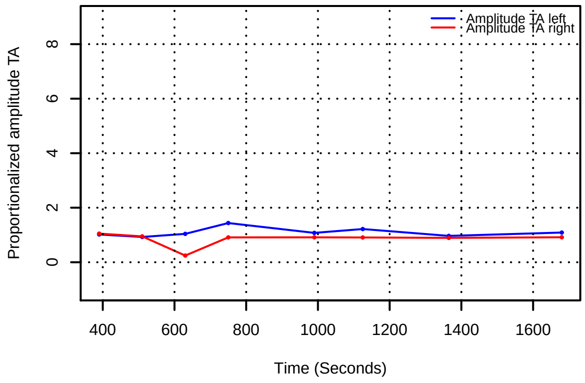
Amplitude TA left
Amplitude TA right
Proportionalized amplitude TA
0
2
4
6
8
400
600
800
1000
1200
1400
1600
Time (Seconds)

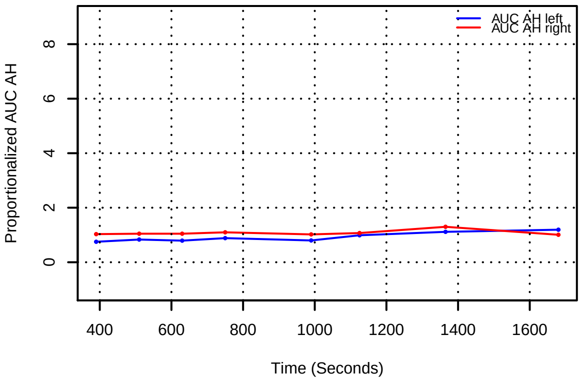
AUC AH left
AUC AH right
Proportionalized AUC AH
0
2
4
6
8
400
600
800
1000
1200
1400
1600
Time (Seconds)

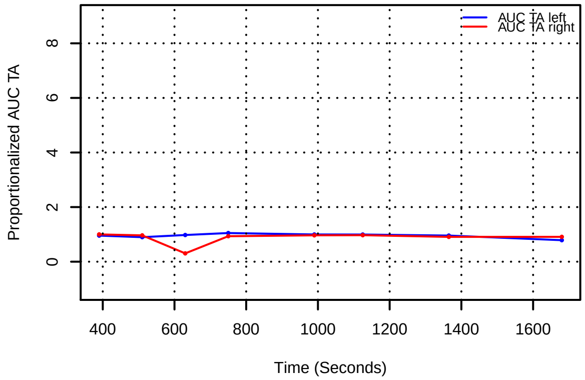
AUC TA left
AUC TA right
Proportionalized AUC TA
0
2
4
6
8
400
600
800
1000
1200
1400
1600
Time (Seconds)

Patient 8

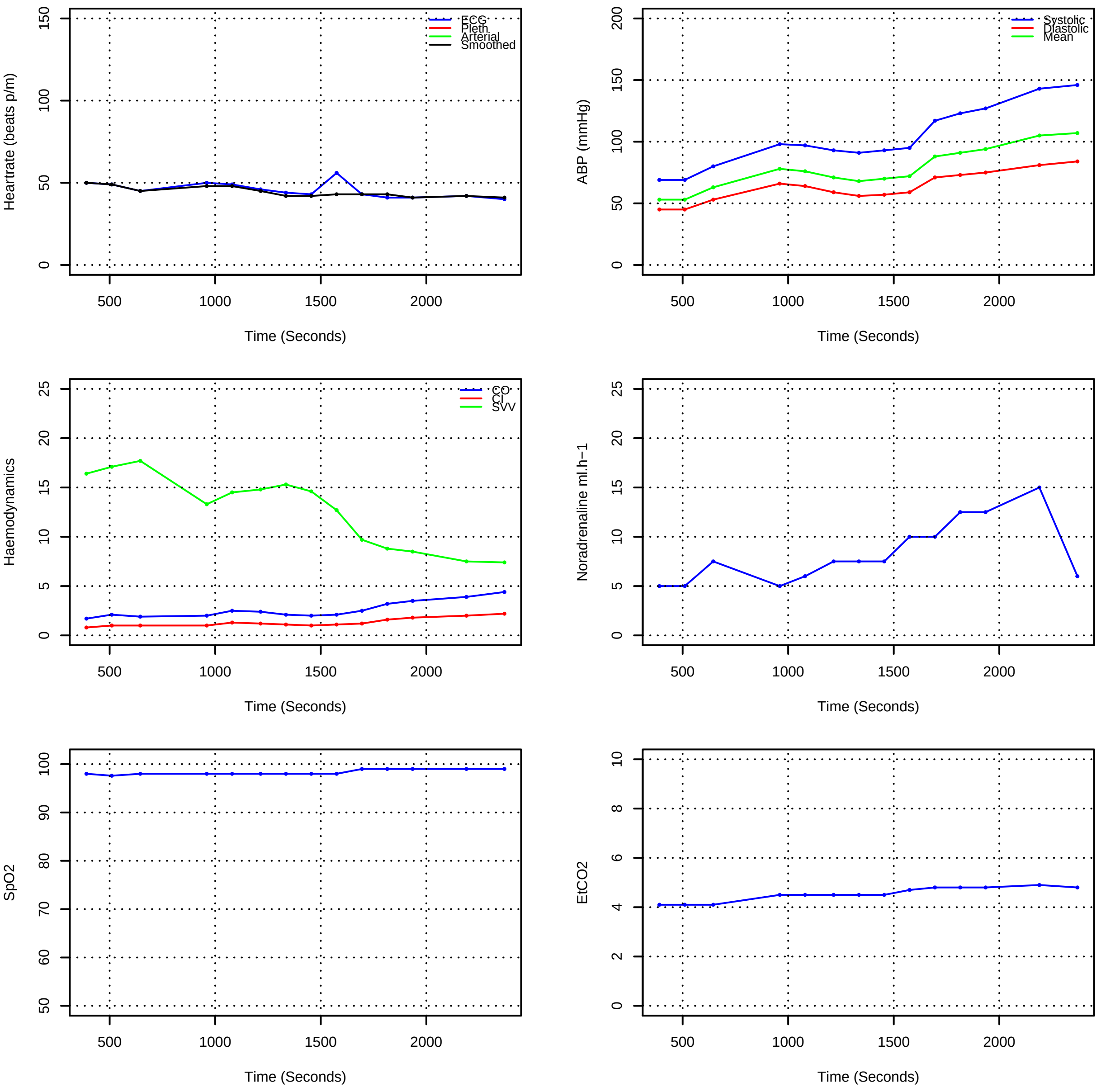

Heartrate (beats p/m)
ECG
Pleth
Arterial
Smoothed
ABP (mmHg)
Systolic
Diastolic
Mean
Haemodynamics
CO
CI
SVV
Noradrenaline ml.h−1
SpO2
EtCO2
Time (Seconds)

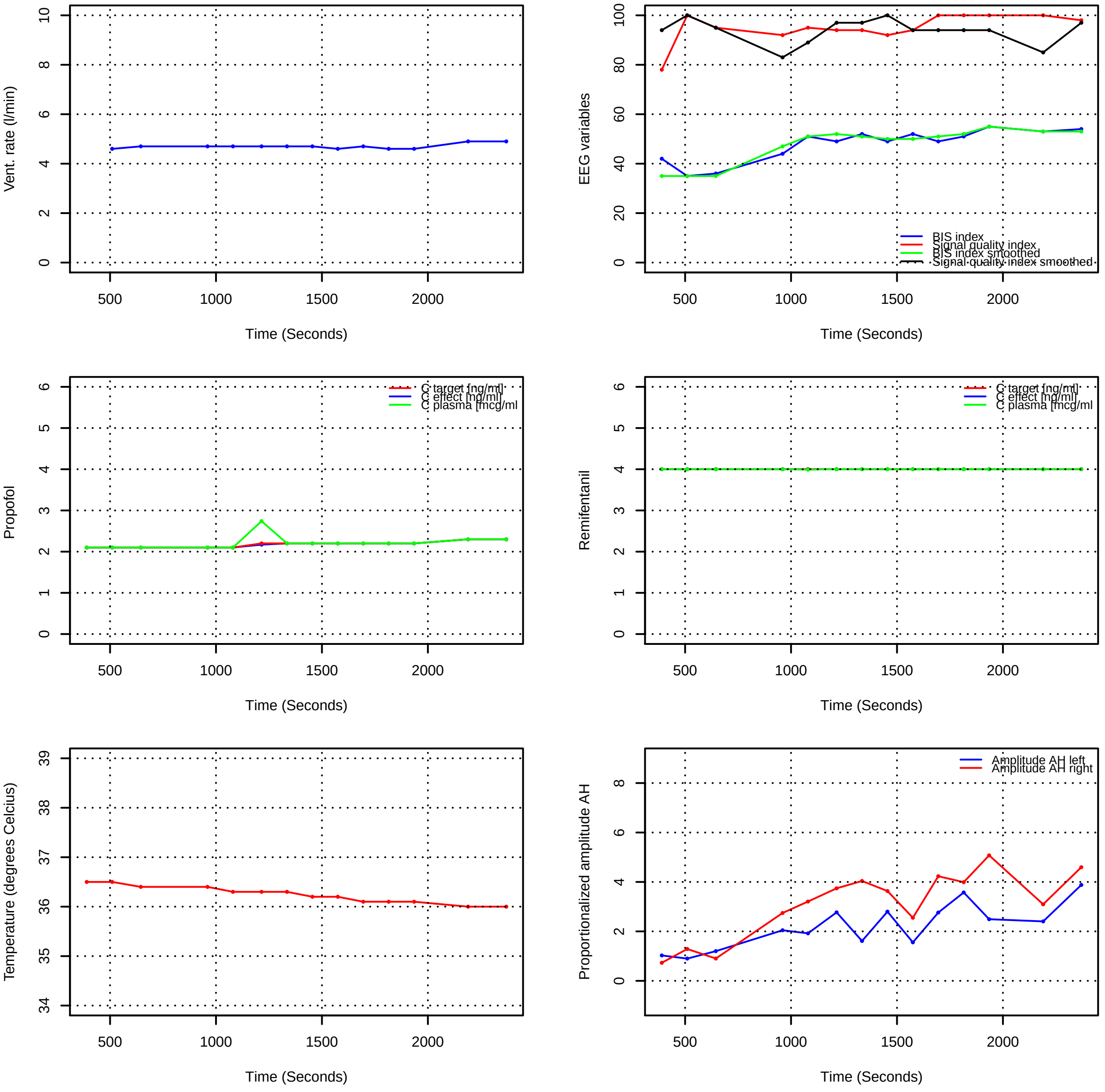

Vent. rate (l/min)
Time (Seconds)
EEG variables
BIS index
Signal quality index
BIS index smoothed
Signal quality index smoothed
Propofol
C target [ng/ml]
C effect [ng/ml]
C plasma [mcg/ml
Remifentanil
Temperature (degrees Celcius)
Proportionalized amplitude AH
Amplitude AH left
Amplitude AH right

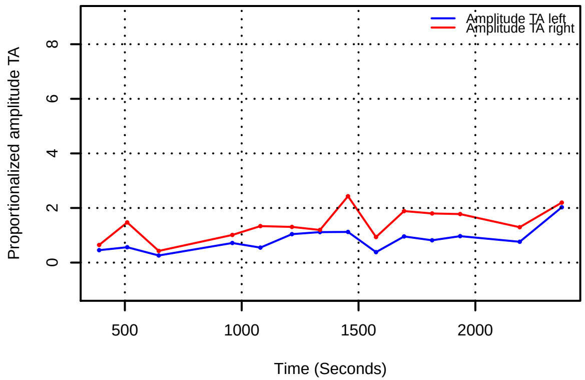

Amplitude TA left
Amplitude TA right
Proportionalized amplitude TA
0
2
4
6
8
500
1000
1500
2000
Time (Seconds)

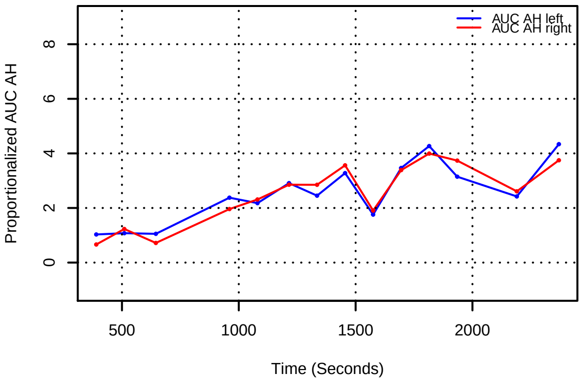

AUC AH left
AUC AH right
Proportionalized AUC AH
0
2
4
6
8
500
1000
1500
2000
Time (Seconds)

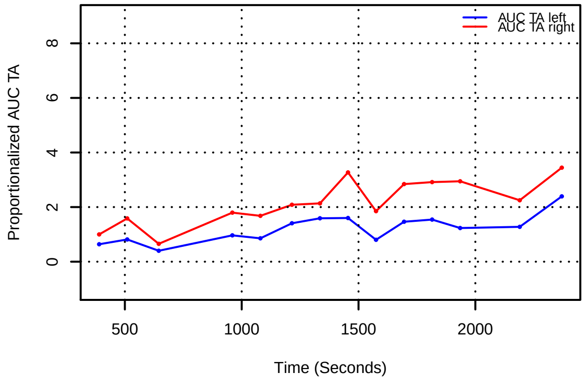

AUC TA left
AUC TA right
Proportionalized AUC TA
0
2
4
6
8
500
1000
1500
2000
Time (Seconds)

Patient 9

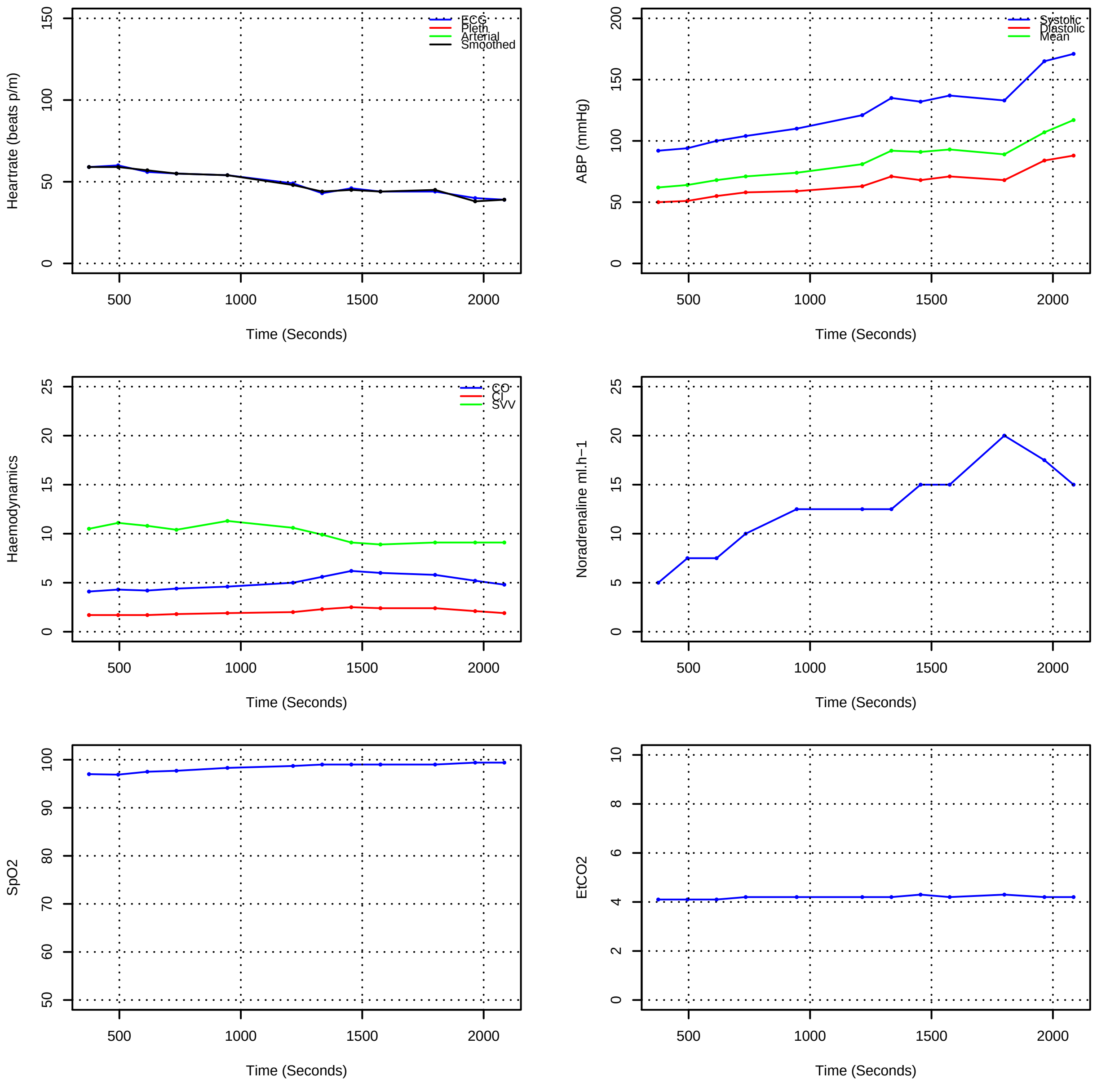
Heartrate (beats p/m)
ECG
Pleth
Arterial
Smoothed
ABP (mmHg)
Systolic
Diastolic
Mean
Haemodynamics
CO
CI
SVV
Noradrenaline ml.h−1
SpO2
EtCO2
Time (Seconds)

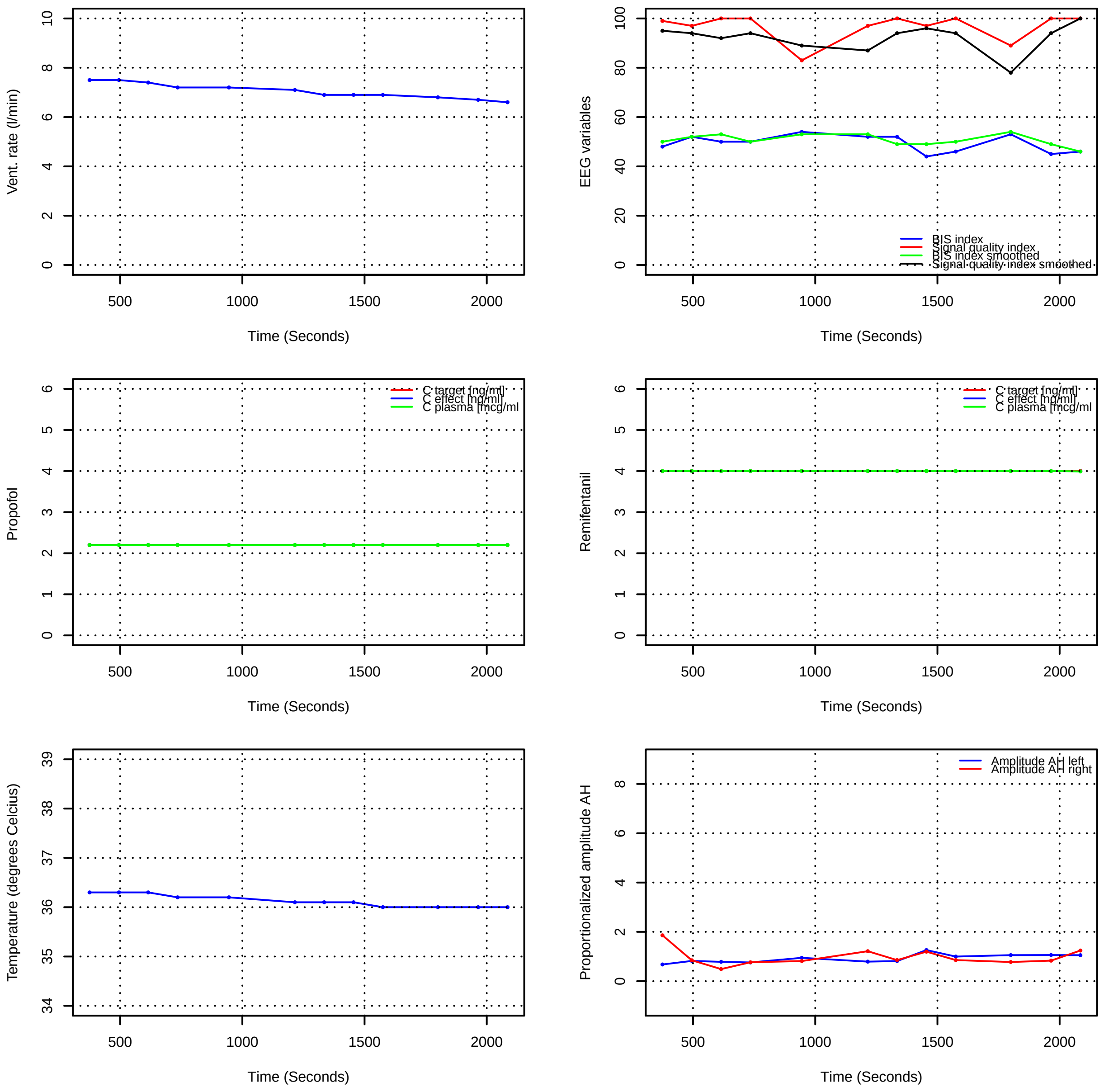
Vent. rate (l/min)
Time (Seconds)
EEG variables
BIS index
Signal quality index
BIS index smoothed
Signal quality index smoothed
Time (Seconds)
Propofol
C target [ng/ml]
C effect [ng/ml]
C plasma [mcg/ml
Time (Seconds)
Remifentanil
C target [ng/ml]
C effect [ng/ml]
C plasma [mcg/ml
Time (Seconds)
Temperature (degrees Celcius)
Time (Seconds)
Proportionalized amplitude AH
Amplitude AH left
Amplitude AH right
Time (Seconds)

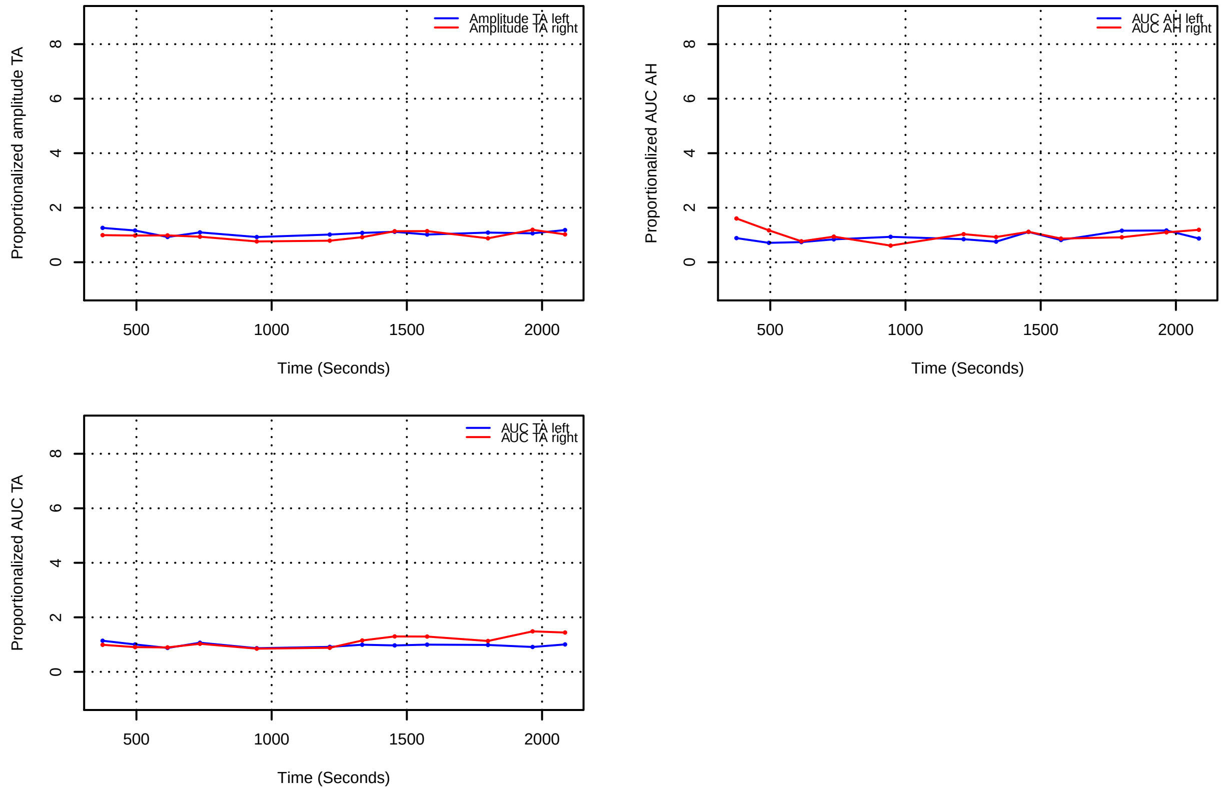
Amplitude TA left
Amplitude TA right
Proportionalized amplitude TA
0
2
4
6
8
500
1000
1500
2000
Time (Seconds)
AUC AH left
AUC AH right
Proportionalized AUC AH
0
2
4
6
8
500
1000
1500
2000
Time (Seconds)
AUC TA left
AUC TA right
Proportionalized AUC TA
0
2
4
6
8
500
1000
1500
2000
Time (Seconds)

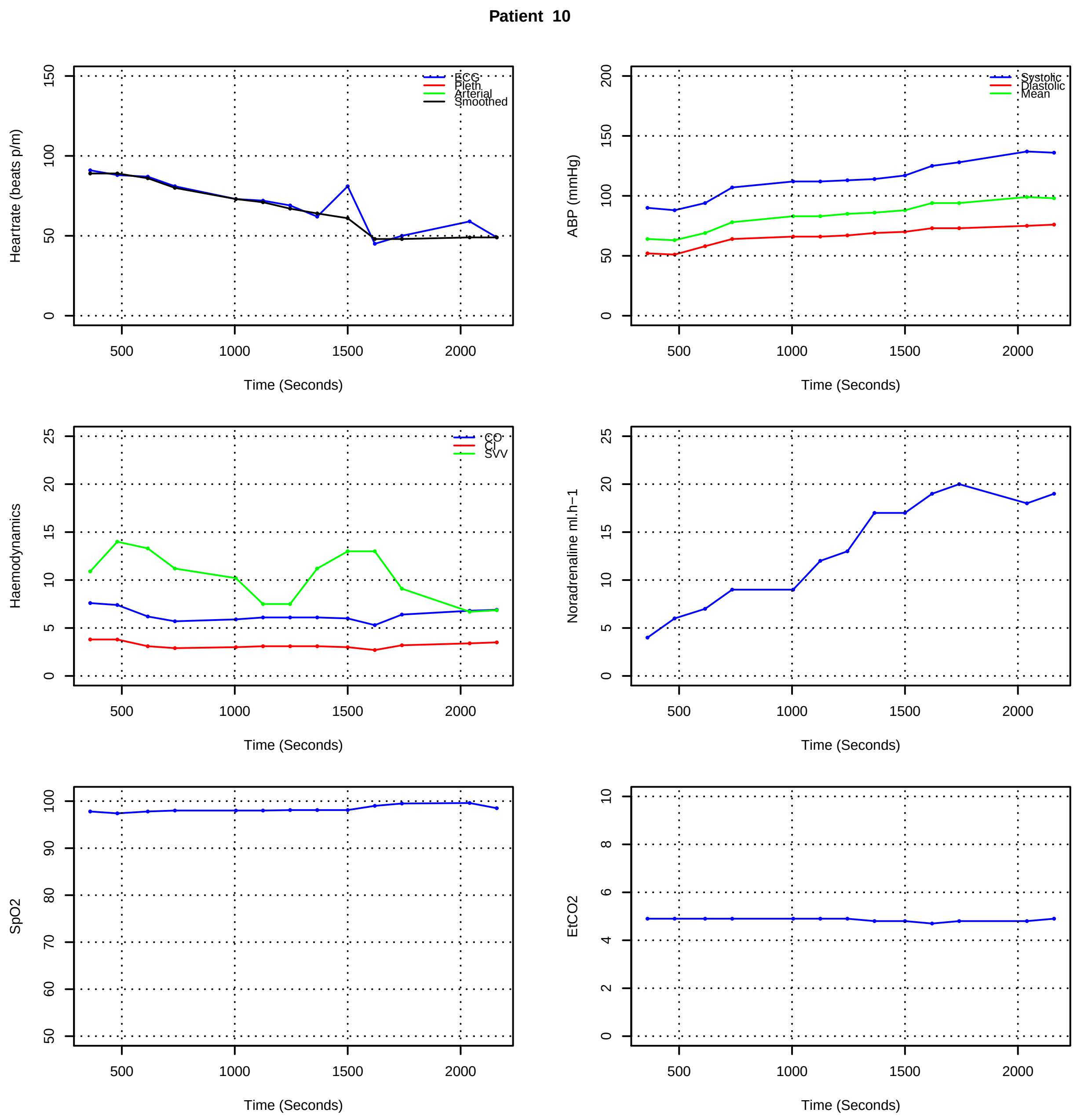
Patient 10
Heartrate (beats p/m)
ECG
Pleth
Arterial
Smoothed
Time (Seconds)
ABP (mmHg)
Systolic
Diastolic
Mean
Time (Seconds)
Haemodynamics
CO
CI
SVV
Time (Seconds)
Noradrenaline ml.h−1
Time (Seconds)
SpO2
Time (Seconds)
EtCO2
Time (Seconds)

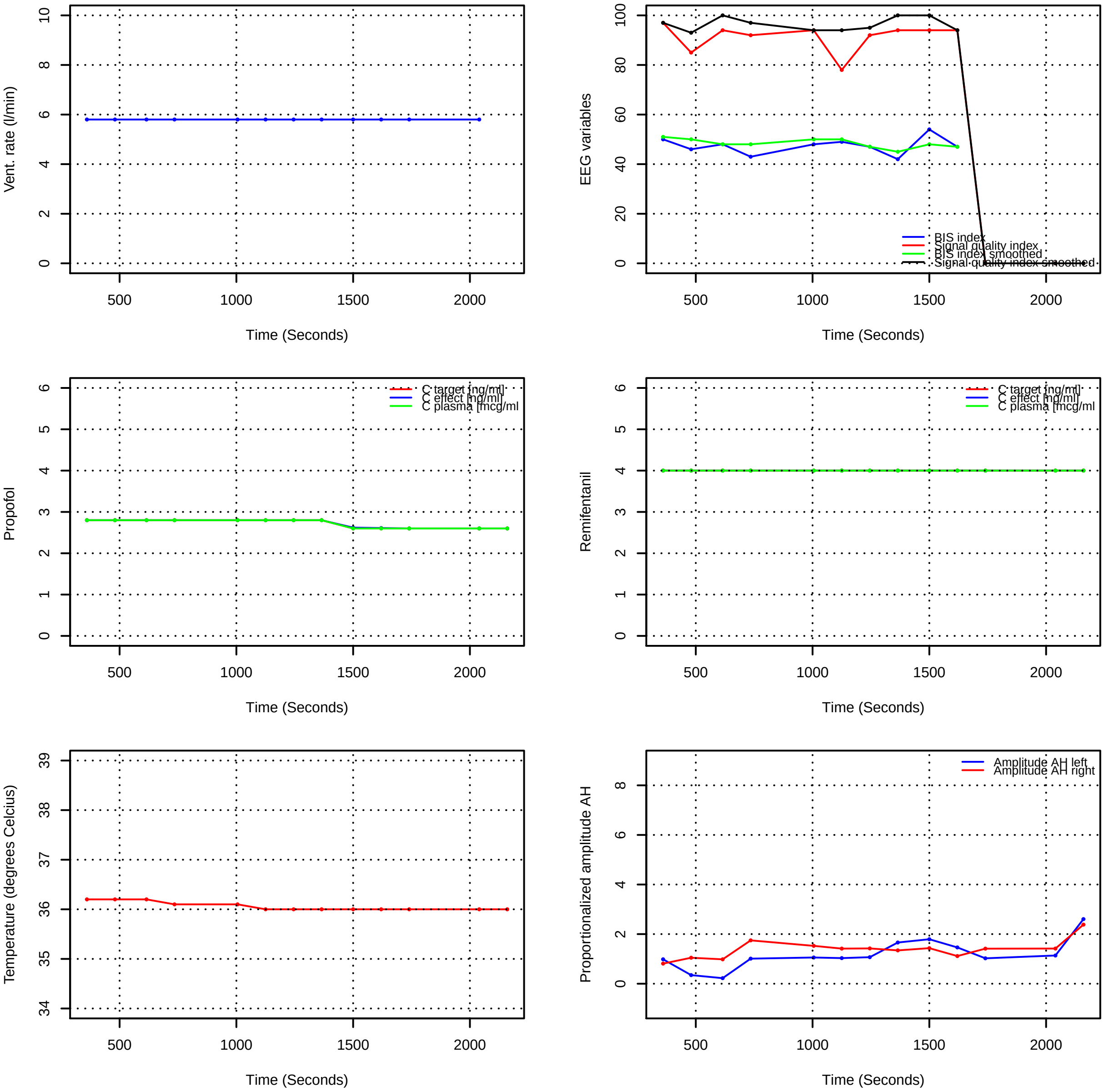
Vent. rate (l/min)
Time (Seconds)
EEG variables
BIS index
Signal quality index
BIS index smoothed
Signal quality index smoothed
Time (Seconds)
Propofol
C target [ng/ml]
C effect [ng/ml]
C plasma [mcg/ml
Time (Seconds)
Remifentanil
C target [ng/ml]
C effect [ng/ml]
C plasma [mcg/ml
Time (Seconds)
Temperature (degrees Celcius)
Time (Seconds)
Proportionalized amplitude AH
Amplitude AH left
Amplitude AH right
Time (Seconds)

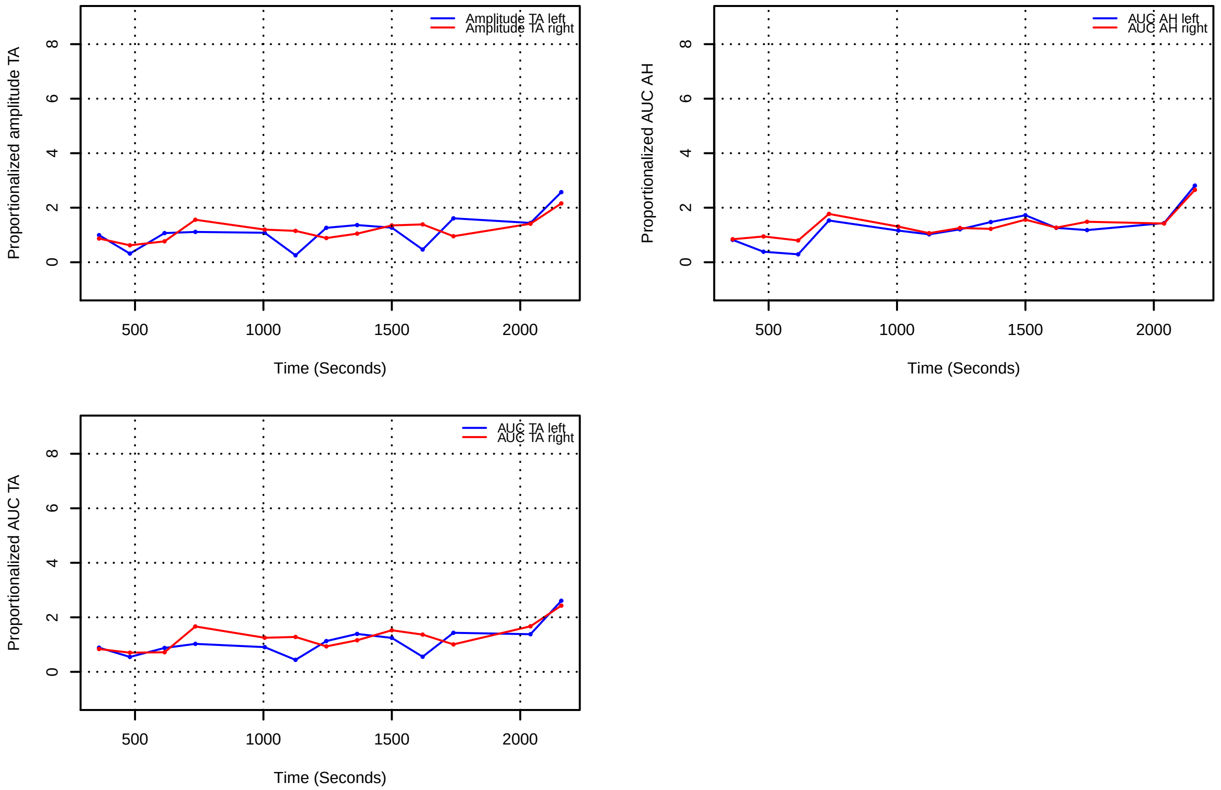

Amplitude TA left
Amplitude TA right
Proportionalized amplitude TA
Time (Seconds)
AUC AH left
AUC AH right
Proportionalized AUC AH
Time (Seconds)
AUC TA left
AUC TA right
Proportionalized AUC TA
Time (Seconds)

Patient 11

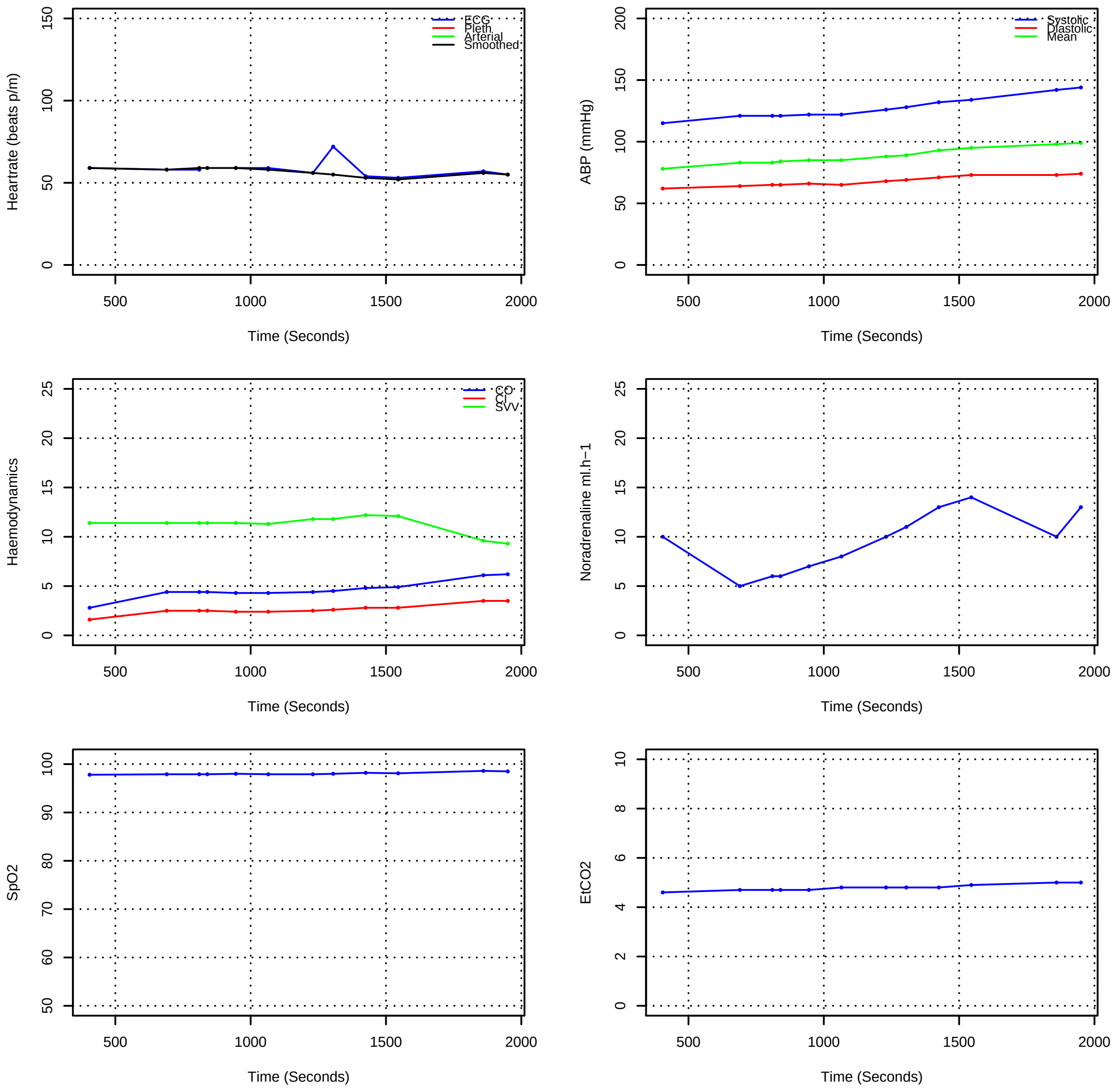
Heartrate (beats p/m)
0
50
100
150
ECG
Pleth
Arterial
Smoothed
500
1000
1500
2000
Time (Seconds)
ABP (mmHg)
0
50
100
150
200
Systolic
Diastolic
Mean
Time (Seconds)
Haemodynamics
0
5
10
15
20
25
CO
CI
SVV
Time (Seconds)
Noradrenaline ml.h−1
Time (Seconds)
SpO2
50
60
70
80
90
100
Time (Seconds)
EtCO2
0
2
4
6
8
10
Time (Seconds)

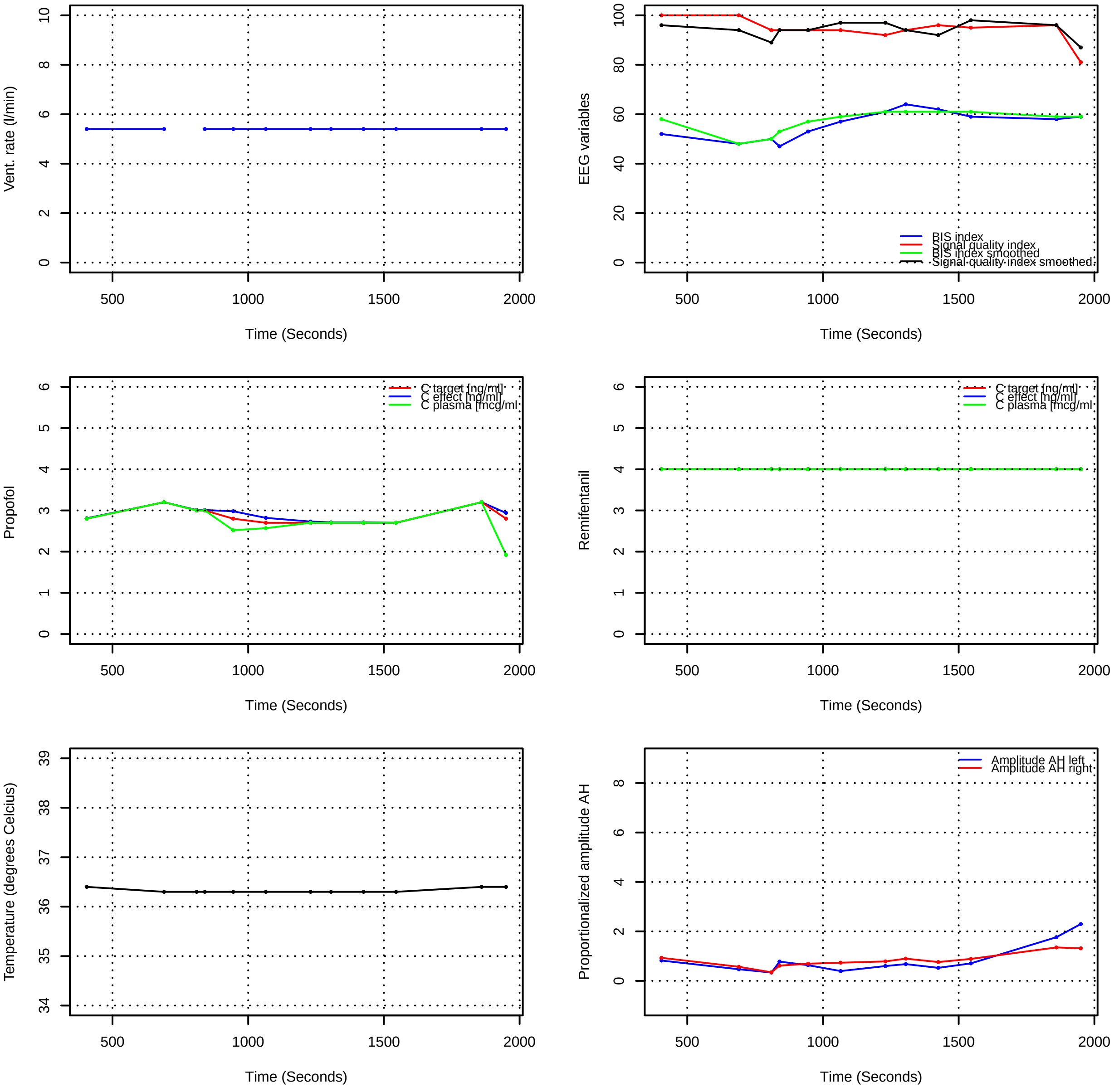
Vent. rate (l/min)
Time (Seconds)
EEG variables
BIS index
Signal quality index
BIS index smoothed
Signal quality index smoothed
Propofol
C target [ng/ml]
C effect [ng/ml]
C plasma [mcg/ml
Remifentanil
Temperature (degrees Celcius)
Proportionalized amplitude AH
Amplitude AH left
Amplitude AH right

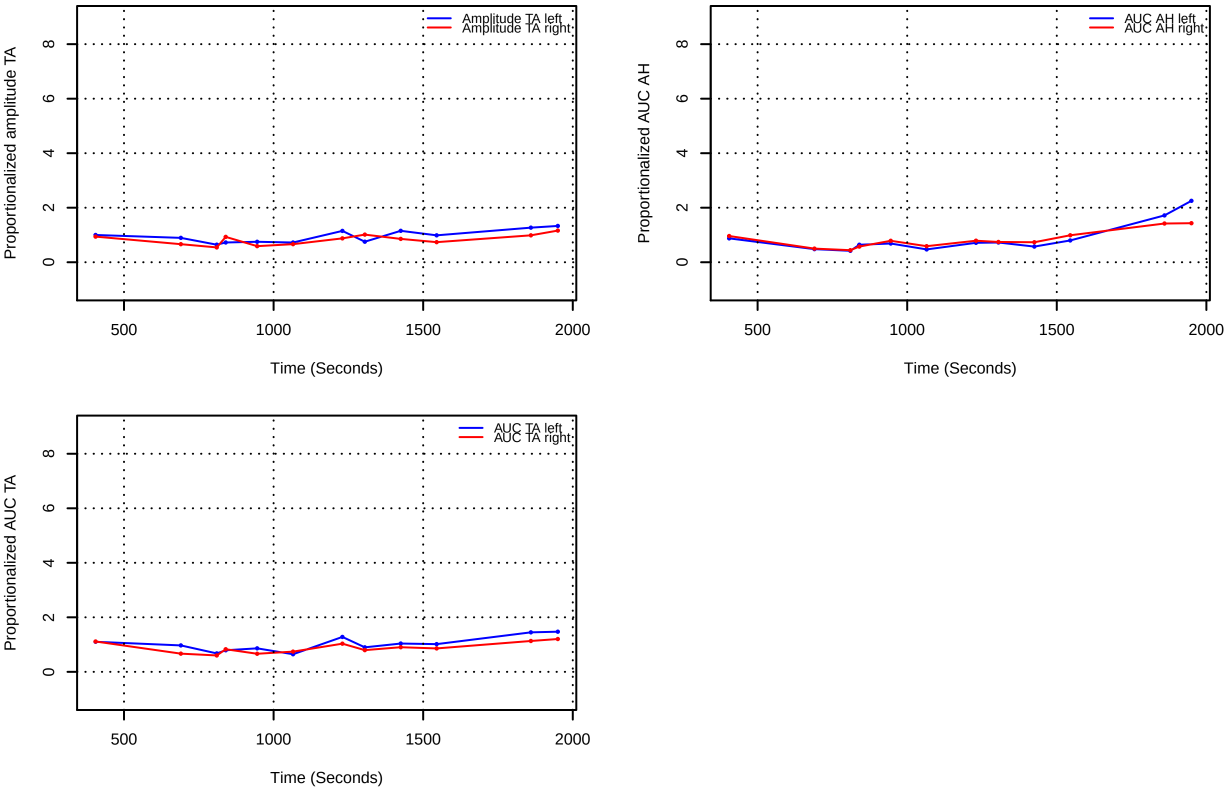
Amplitude TA left
Amplitude TA right
Proportionalized amplitude TA
Time (Seconds)
AUC AH left
AUC AH right
Proportionalized AUC AH
Time (Seconds)
AUC TA left
AUC TA right
Proportionalized AUC TA
Time (Seconds)

Patient 12

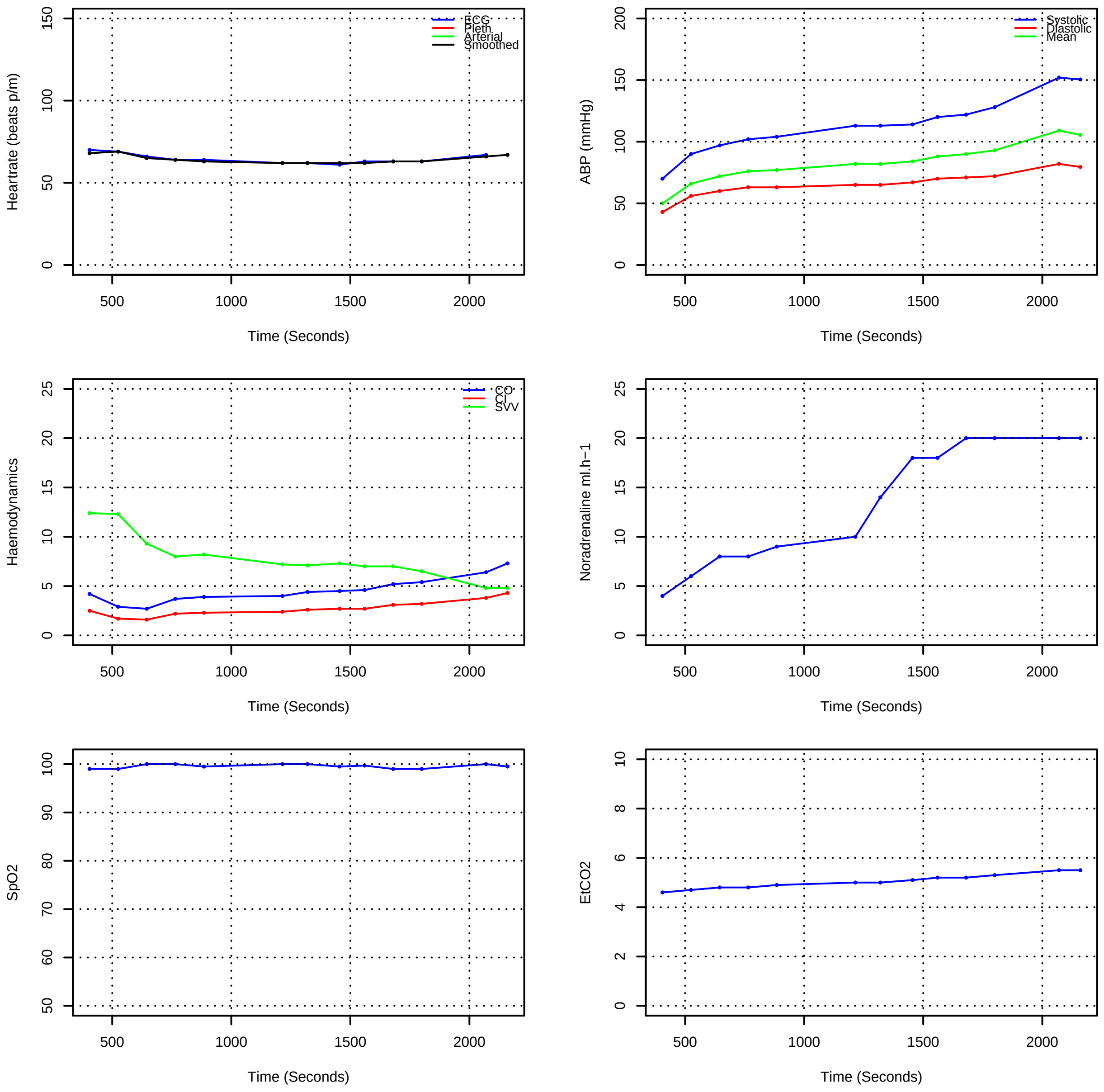
ECG
Pleth
Arterial
Smoothed
Heartrate (beats p/m)
0
50
100
150
500
1000
1500
2000
Time (Seconds)
Systolic
Diastolic
Mean
ABP (mmHg)
0
50
100
150
200
500
1000
1500
2000
Time (Seconds)
CO
CI
SVV
Haemodynamics
0
5
10
15
20
25
500
1000
1500
2000
Time (Seconds)
Noradrenaline ml.h−1
0
5
10
15
20
25
500
1000
1500
2000
Time (Seconds)
SpO2
50
60
70
80
90
100
500
1000
1500
2000
Time (Seconds)
EtCO2
0
2
4
6
8
10
500
1000
1500
2000
Time (Seconds)

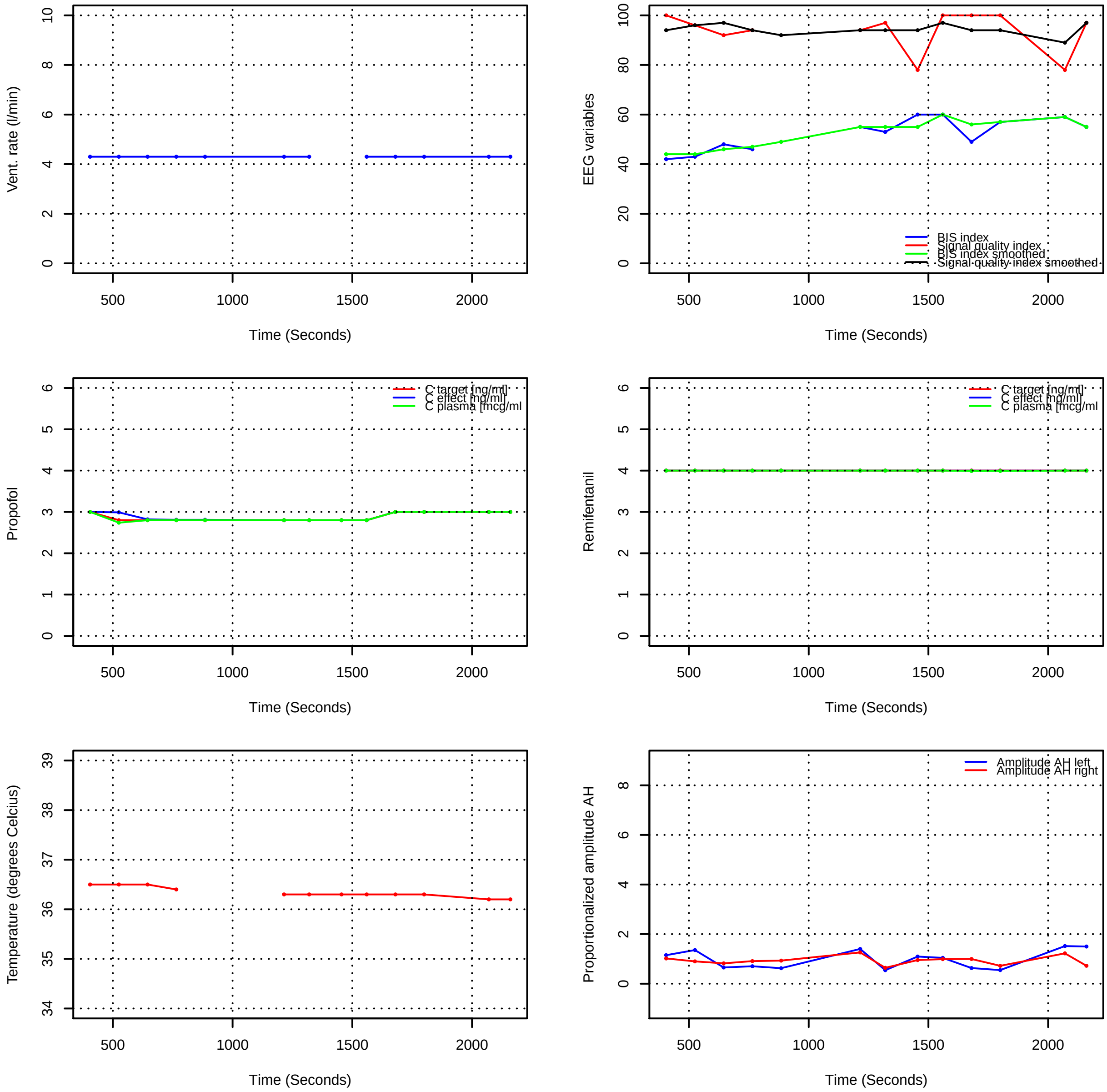

Vent. rate (l/min)
Time (Seconds)
EEG variables
BIS index
Signal quality index
BIS index smoothed
Signal quality index smoothed
Time (Seconds)
Propofol
C target [ng/ml]
C effect [ng/ml]
C plasma [mcg/ml
Time (Seconds)
Remifentanil
C target [ng/ml]
C effect [ng/ml]
C plasma [mcg/ml
Time (Seconds)
Temperature (degrees Celcius)
Time (Seconds)
Proportionalized amplitude AH
Amplitude AH left
Amplitude AH right
Time (Seconds)

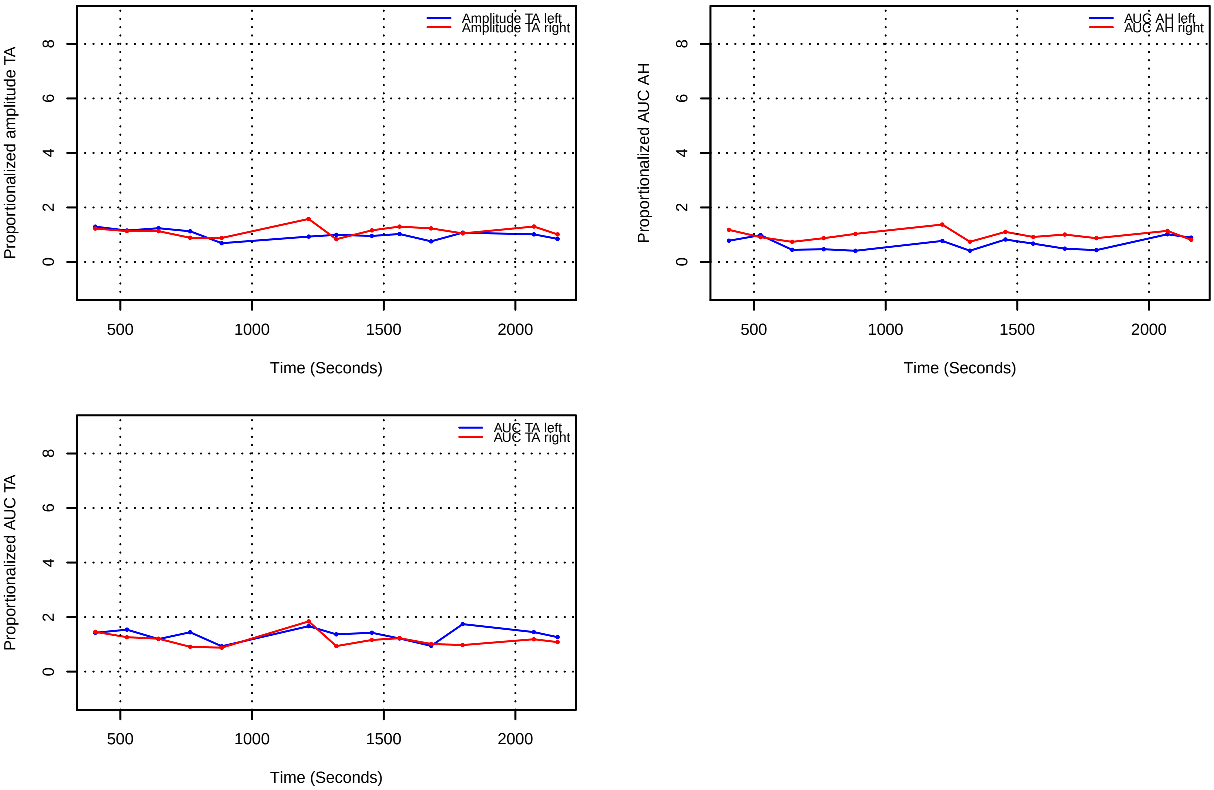
Amplitude TA left
Amplitude TA right
Proportionalized amplitude TA
0
2
4
6
8
500
1000
1500
2000
Time (Seconds)
AUC AH left
AUC AH right
Proportionalized AUC AH
0
2
4
6
8
500
1000
1500
2000
Time (Seconds)
AUC TA left
AUC TA right
Proportionalized AUC TA
0
2
4
6
8
500
1000
1500
2000
Time (Seconds)

Patient 13

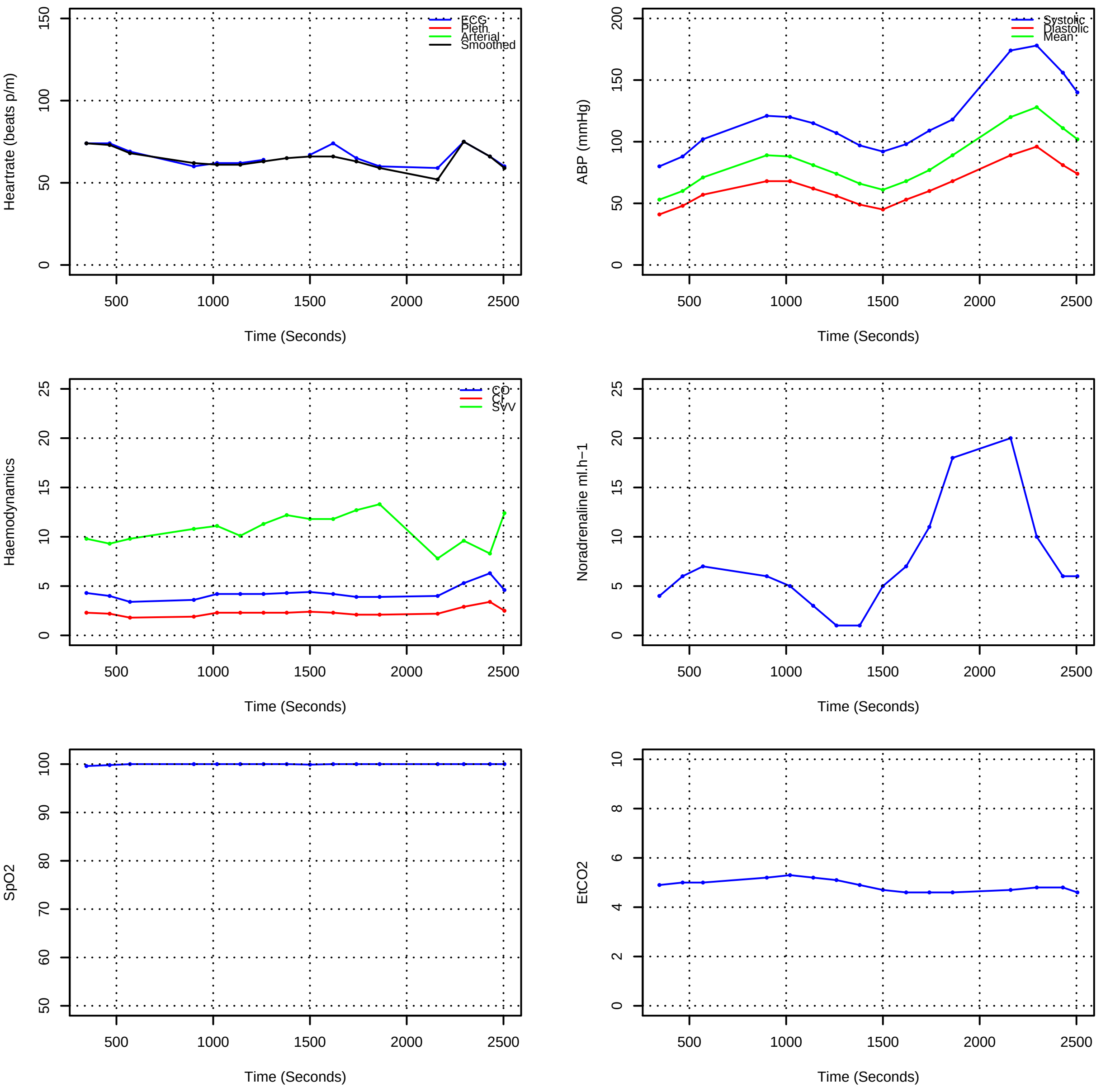
Heartrate (beats p/m)
ECG
Pleth
Arterial
Smoothed
ABP (mmHg)
Systolic
Diastolic
Mean
Haemodynamics
CO
CI
SVV
Noradrenaline ml.h−1
SpO2
EtCO2
Time (Seconds)

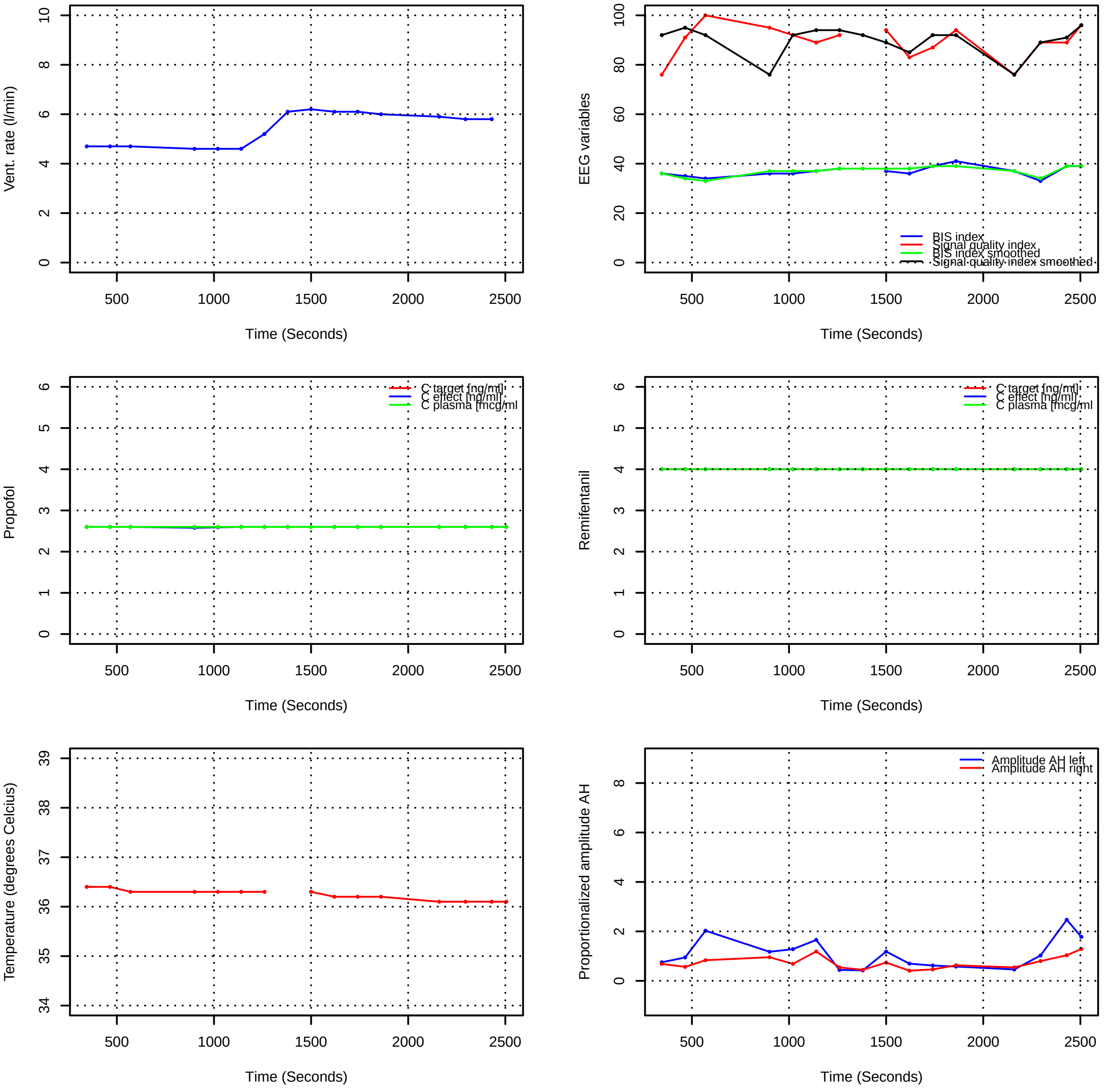

Vent. rate (l/min)
Time (Seconds)
EEG variables
BIS index
Signal quality index
BIS index smoothed
Signal quality index smoothed
Time (Seconds)
Propofol
C target [ng/ml]
C effect [ng/ml]
C plasma [mcg/ml
Time (Seconds)
Remifentanil
C target [ng/ml]
C effect [ng/ml]
C plasma [mcg/ml
Time (Seconds)
Temperature (degrees Celcius)
Time (Seconds)
Proportionalized amplitude AH
Amplitude AH left
Amplitude AH right
Time (Seconds)

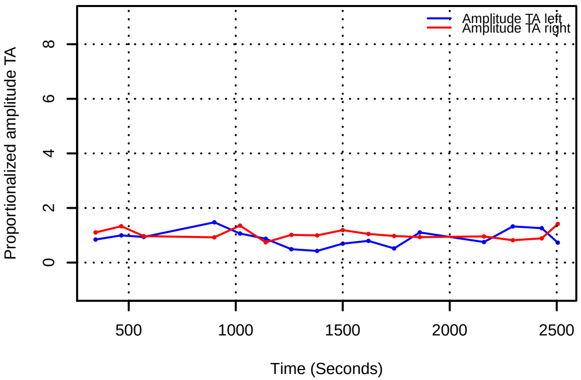
Amplitude TA left
Amplitude TA right
Proportionalized amplitude TA
0
2
4
6
8
500
1000
1500
2000
2500
Time (Seconds)

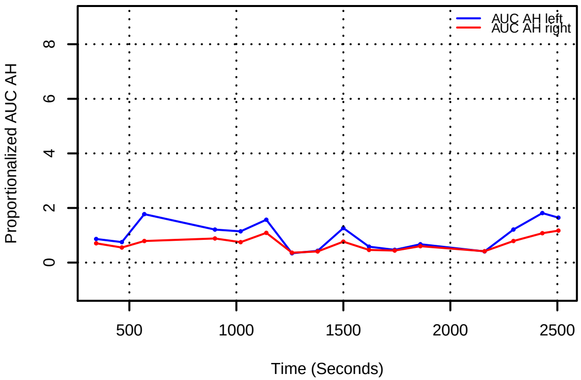
AUC AH left
AUC AH right
Proportionalized AUC AH
0
2
4
6
8
500
1000
1500
2000
2500
Time (Seconds)

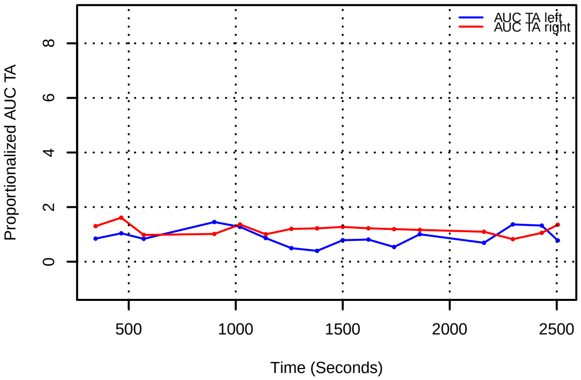
AUC TA left
AUC TA right
Proportionalized AUC TA
0
2
4
6
8
500
1000
1500
2000
2500
Time (Seconds)

Patient 14

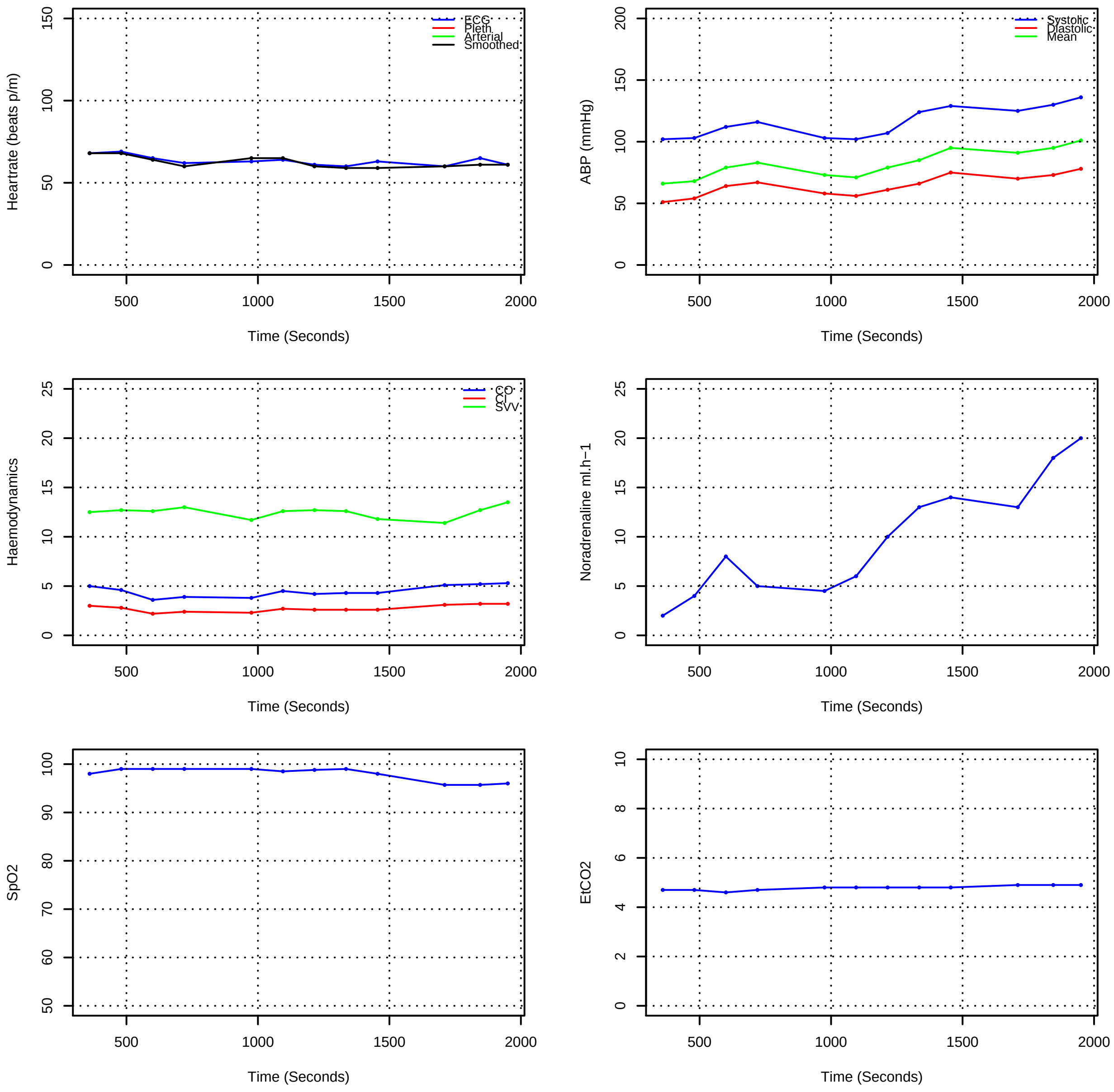
Heartrate (beats p/m)
ECG
Pleth
Arterial
Smoothed
ABP (mmHg)
Systolic
Diastolic
Mean
Haemodynamics
CO
CI
SVV
Noradrenaline ml.h−1
SpO2
EtCO2
Time (Seconds)

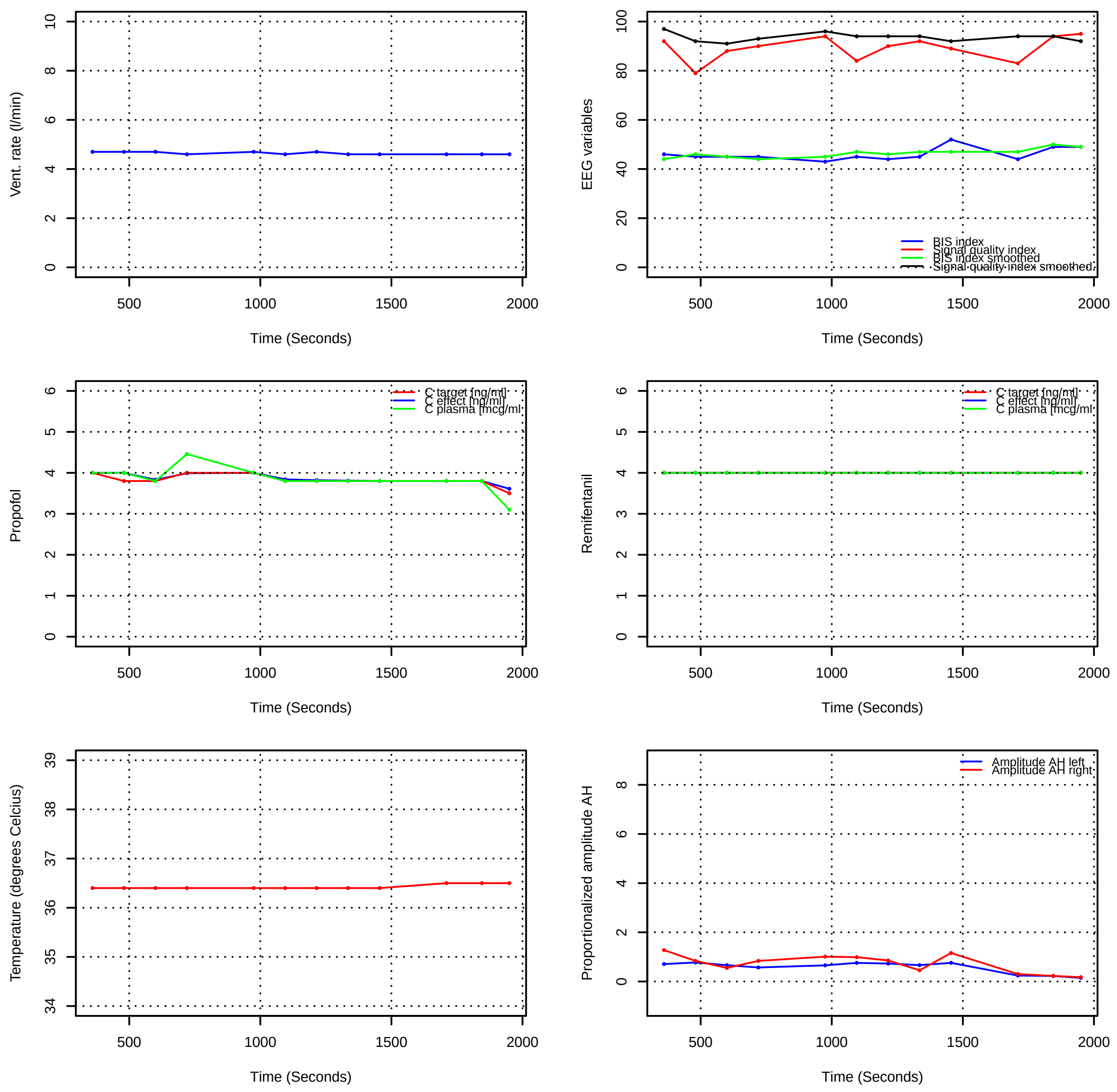

Vent. rate (l/min)
EEG variables
BIS index
Signal quality index
BIS index smoothed
Signal quality index smoothed
Propofol
Remifentanil
C target [ng/ml]
C effect [ng/ml]
C plasma [mcg/ml
Temperature (degrees Celcius)
Proportionalized amplitude AH
Amplitude AH left
Amplitude AH right
Time (Seconds)

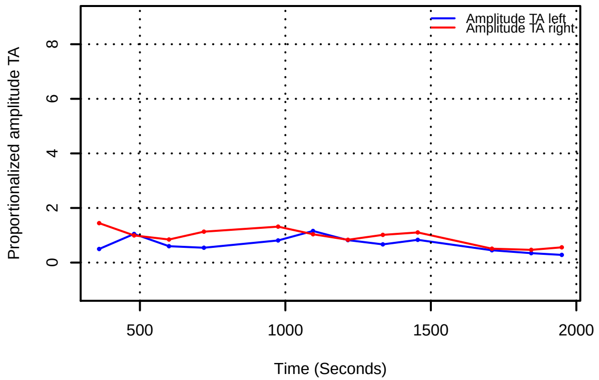
Amplitude TA left
Amplitude TA right
Proportionalized amplitude TA
Time (Seconds)

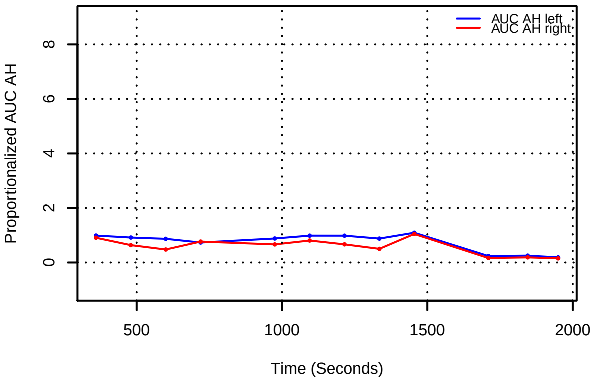
AUC AH left
AUC AH right
Proportionalized AUC AH
Time (Seconds)

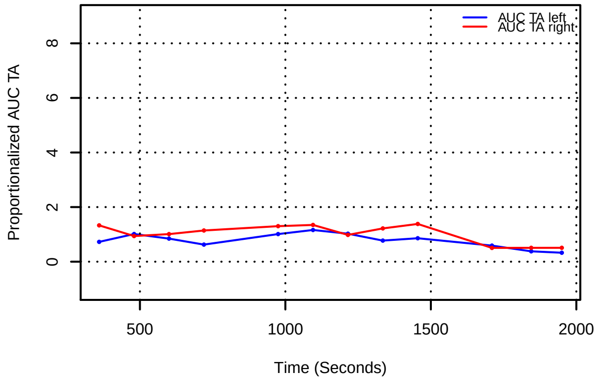
AUC TA left
AUC TA right
Proportionalized AUC TA
Time (Seconds)

Patient 15

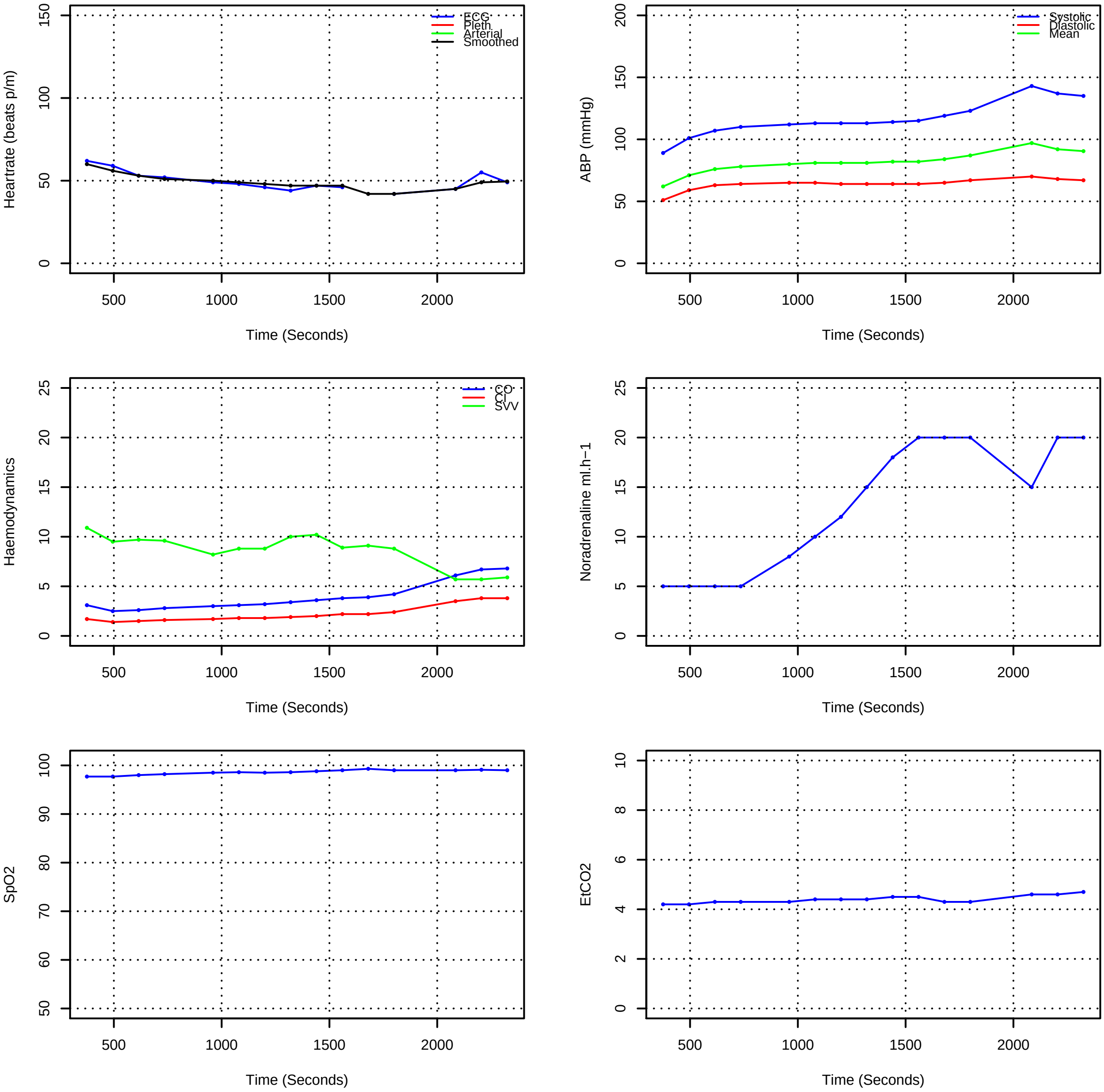

Heartrate (beats p/m)
ECG
Pleth
Arterial
Smoothed
Time (Seconds)
ABP (mmHg)
Systolic
Diastolic
Mean
Time (Seconds)
Haemodynamics
CO
CI
SVV
Time (Seconds)
Noradrenaline ml.h−1
Time (Seconds)
SpO2
Time (Seconds)
EtCO2
Time (Seconds)

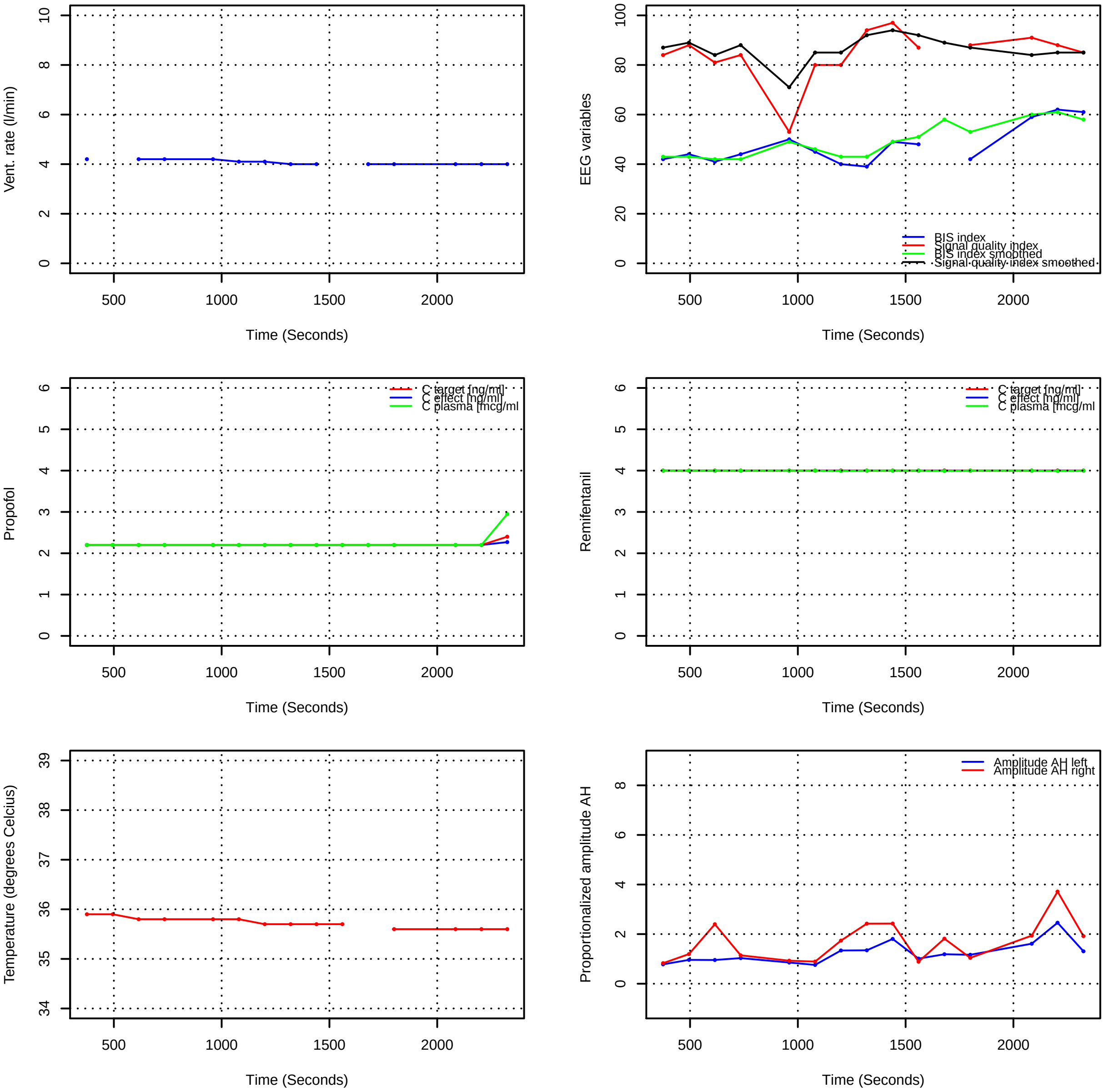

Vent. rate (l/min)
Time (Seconds)
EEG variables
BIS index
Signal quality index
BIS index smoothed
Signal quality index smoothed
Propofol
C target [ng/ml]
C effect [ng/ml]
C plasma [mcg/ml
Remifentanil
Temperature (degrees Celcius)
Proportionalized amplitude AH
Amplitude AH left
Amplitude AH right

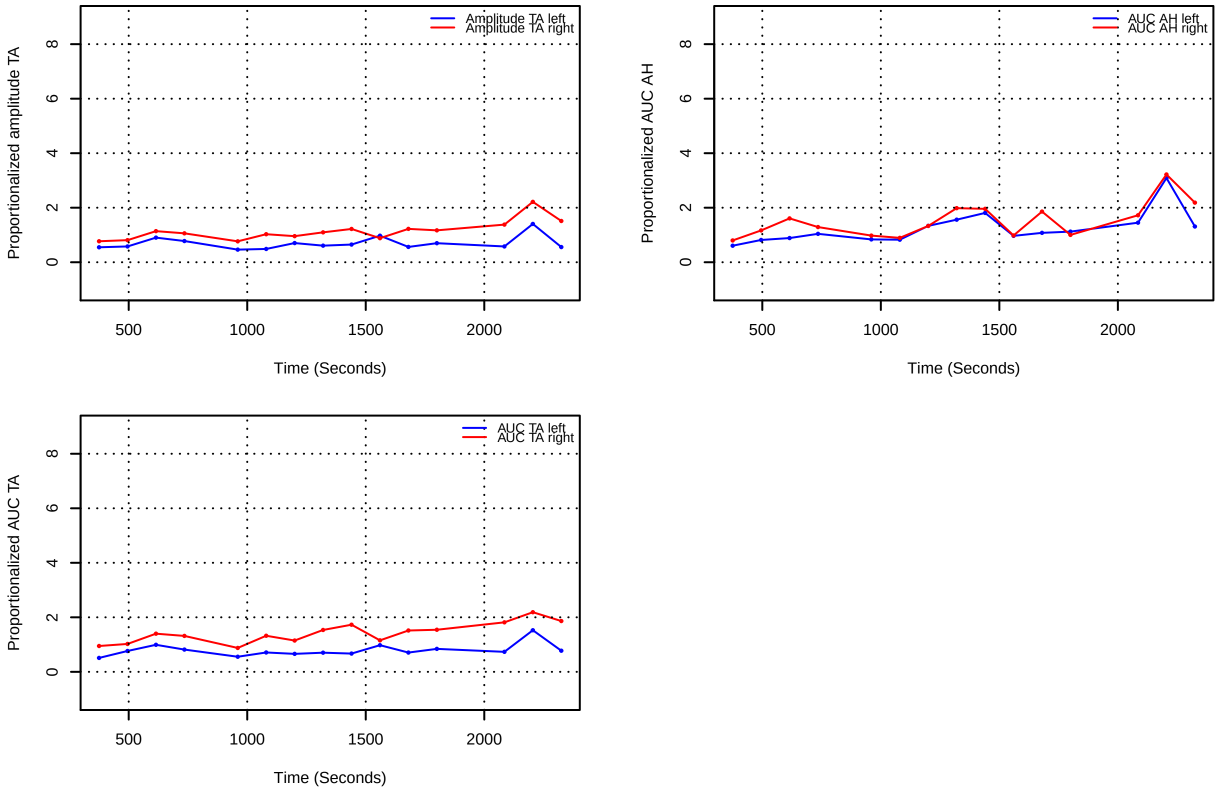

Amplitude TA left
Amplitude TA right
Proportionalized amplitude TA
Time (Seconds)
AUC AH left
AUC AH right
Proportionalized AUC AH
Time (Seconds)
AUC TA left
AUC TA right
Proportionalized AUC TA
Time (Seconds)

Patient 16

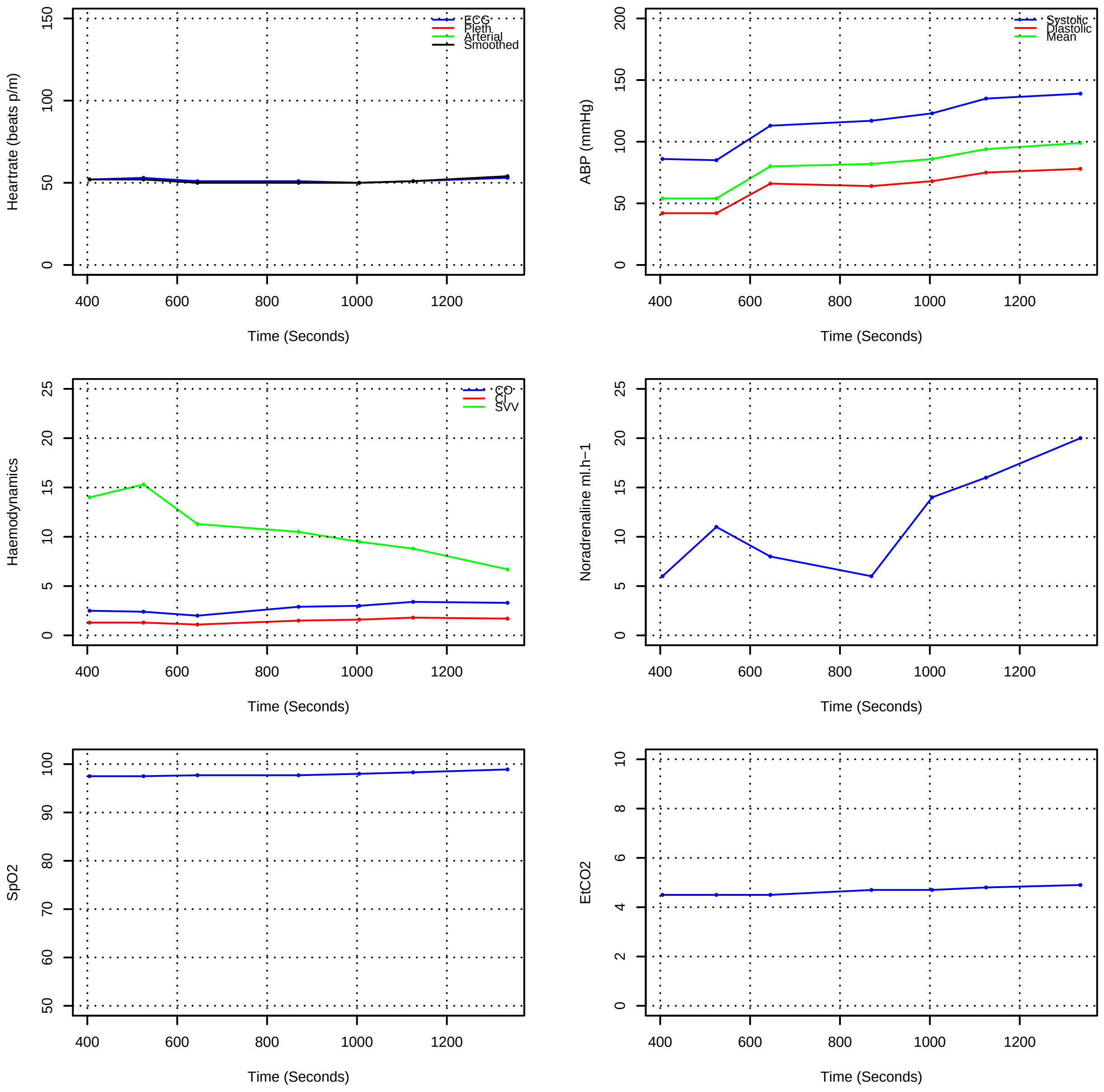

Heartrate (beats p/m)
ECG
Pleth
Arterial
Smoothed
Time (Seconds)
ABP (mmHg)
Systolic
Diastolic
Mean
Time (Seconds)
Haemodynamics
CO
CI
SVV
Time (Seconds)
Noradrenaline ml.h−1
Time (Seconds)
SpO2
Time (Seconds)
EtCO2
Time (Seconds)

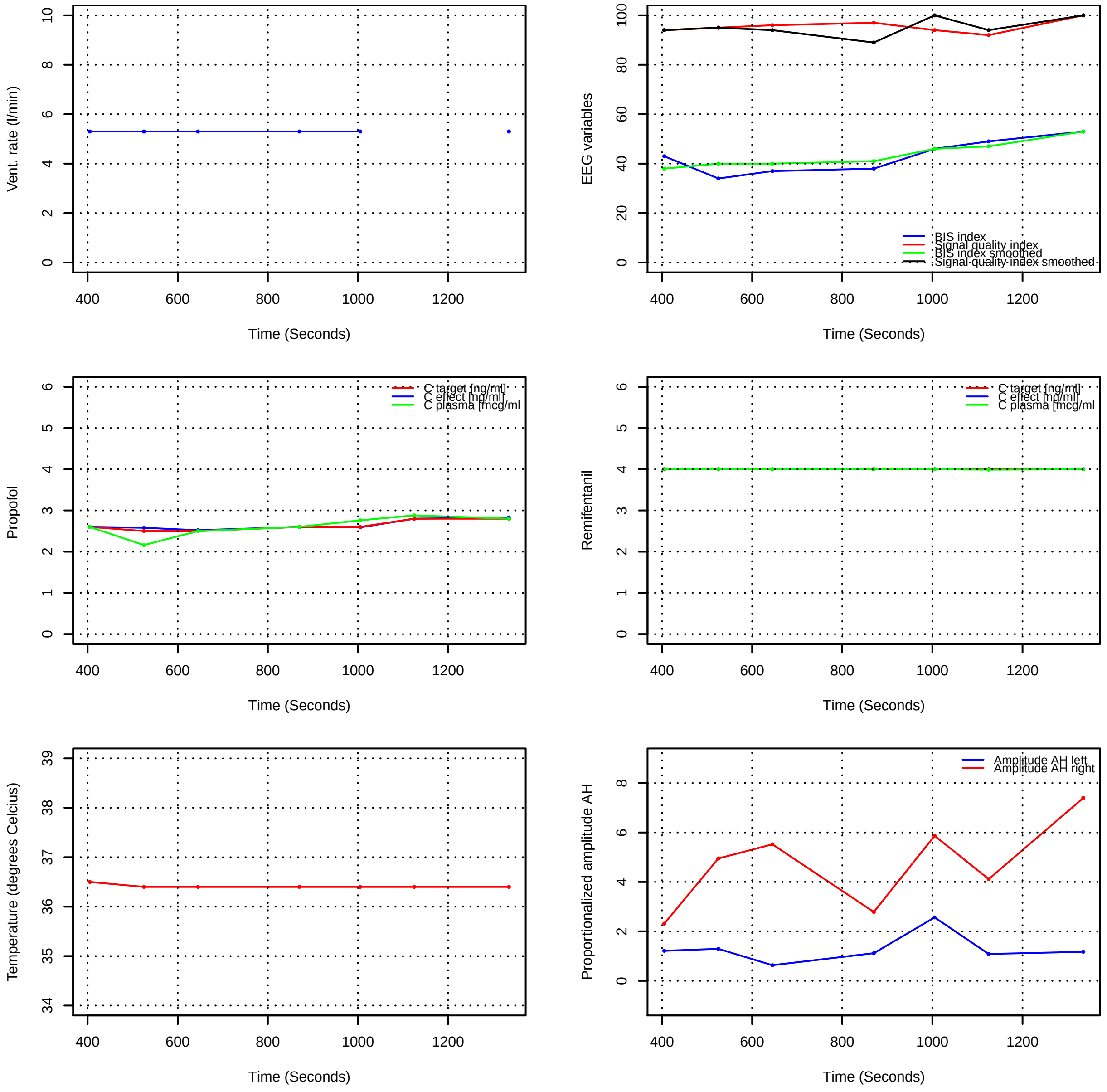
Vent. rate (l/min)
EEG variables
BIS index
Signal quality index
BIS index smoothed
Signal quality index smoothed
Propofol
C target [ng/ml]
C effect [ng/ml]
C plasma [mcg/ml
Remifentanil
Temperature (degrees Celcius)
Proportionalized amplitude AH
Amplitude AH left
Amplitude AH right
Time (Seconds)

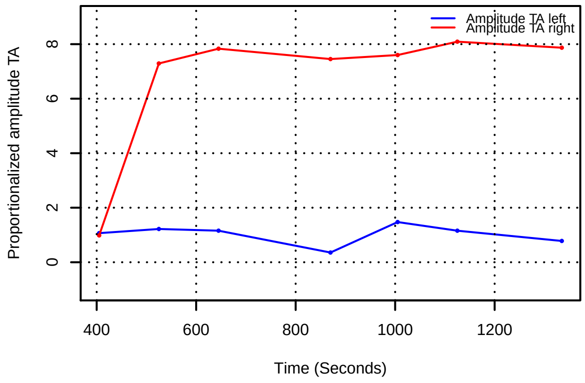
Amplitude TA left
Amplitude TA right
Proportionalized amplitude TA
Time (Seconds)

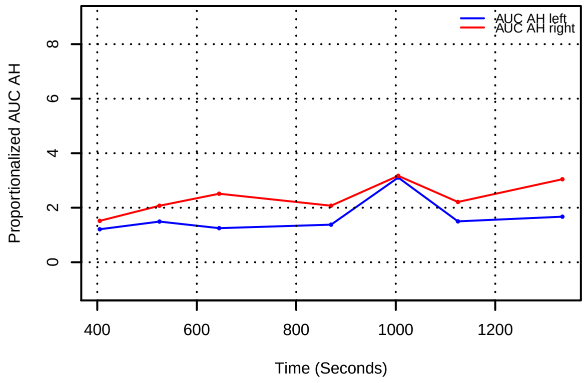
AUC AH left
AUC AH right
Proportionalized AUC AH
Time (Seconds)

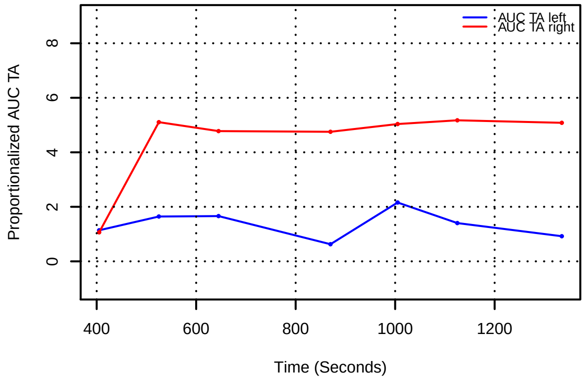
AUC TA left
AUC TA right
Proportionalized AUC TA
Time (Seconds)

Patient 17

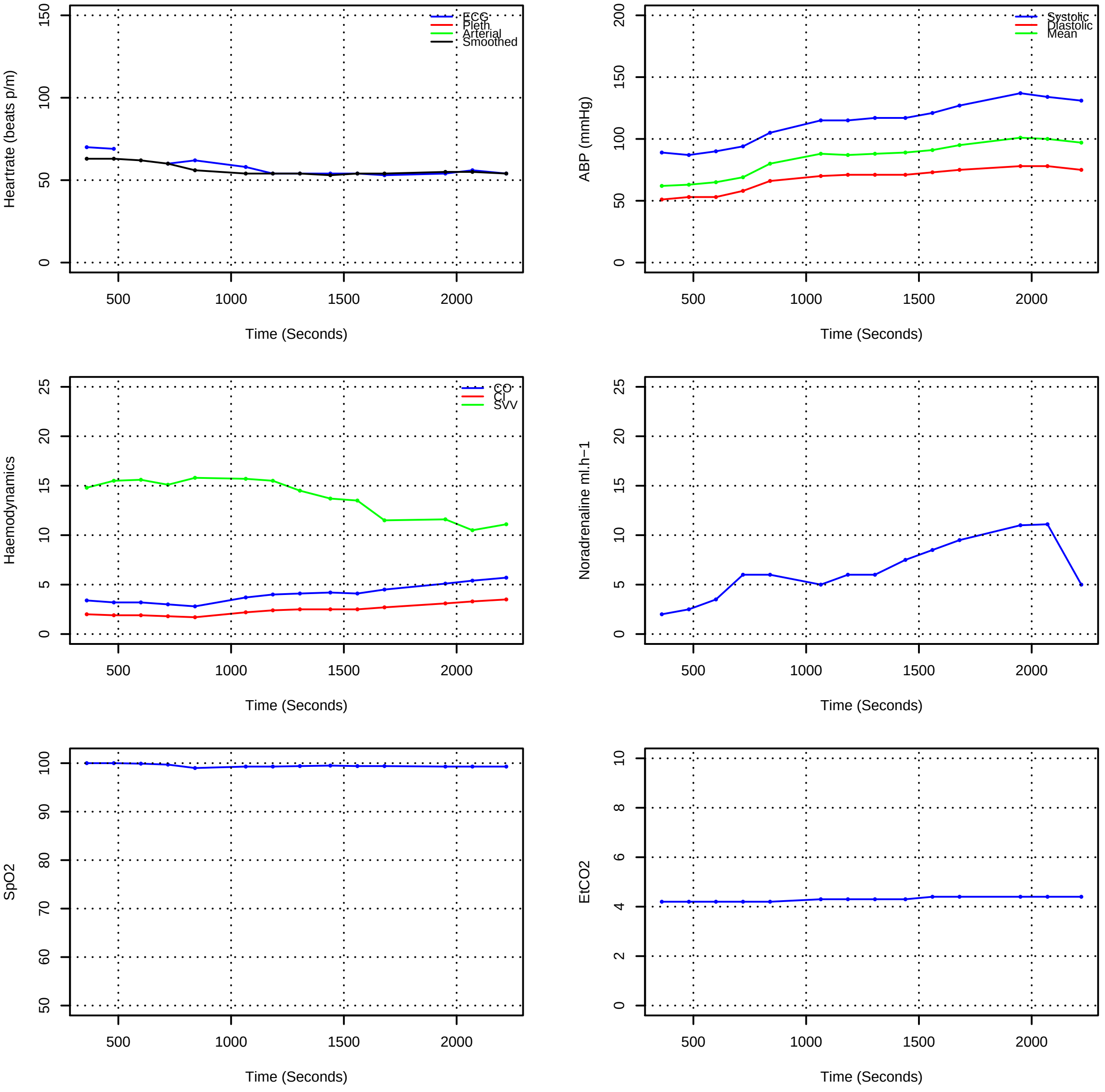
Heartrate (beats p/m)
Time (Seconds)
ECG
Pleth
Arterial
Smoothed
ABP (mmHg)
Systolic
Diastolic
Mean
Haemodynamics
CO
CI
SVV
Noradrenaline ml.h−1
SpO2
EtCO2

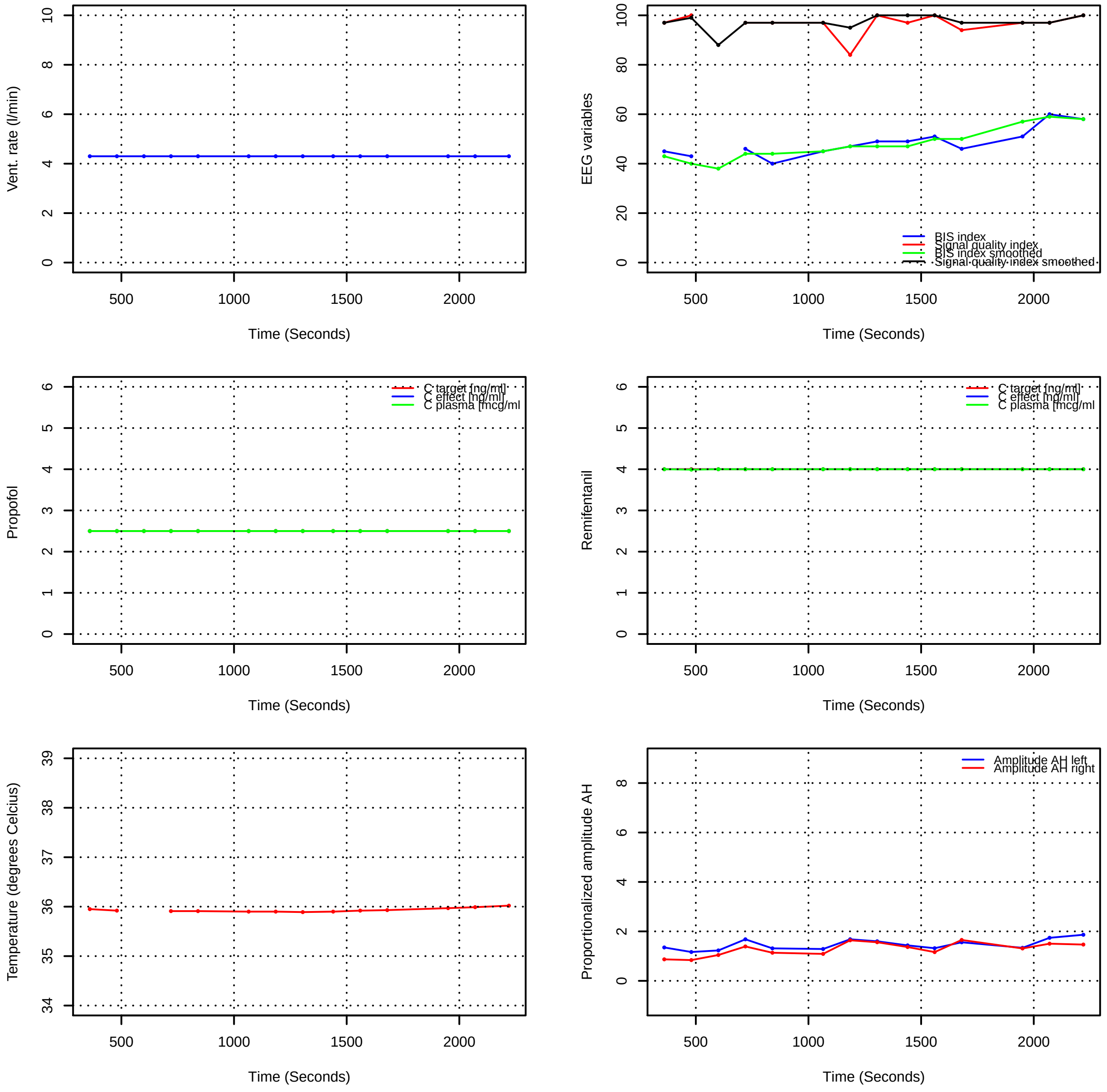

Vent. rate (l/min)
Time (Seconds)
EEG variables
BIS index
Signal quality index
BIS index smoothed
Signal quality index smoothed
Time (Seconds)
Propofol
C target [ng/ml]
C effect [ng/ml]
C plasma [mcg/ml
Time (Seconds)
Remifentanil
C target [ng/ml]
C effect [ng/ml]
C plasma [mcg/ml
Time (Seconds)
Temperature (degrees Celcius)
Time (Seconds)
Proportionalized amplitude AH
Amplitude AH left
Amplitude AH right
Time (Seconds)

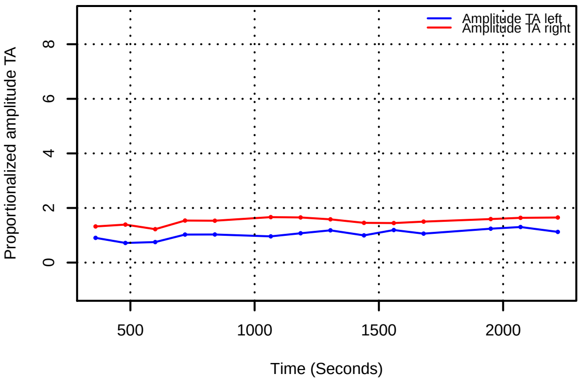
Amplitude TA left
Amplitude TA right
Proportionalized amplitude TA
Time (Seconds)
0
2
4
6
8
500
1000
1500
2000

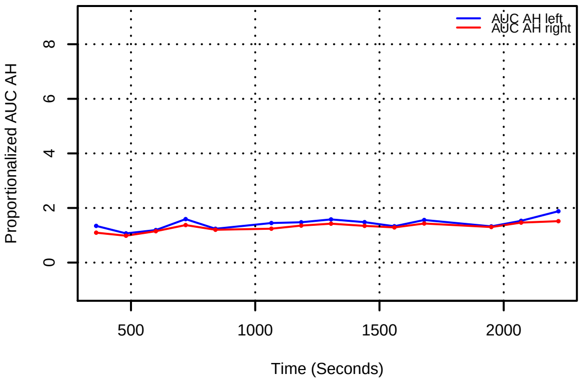
AUC AH left
AUC AH right
Proportionalized AUC AH
Time (Seconds)
0
2
4
6
8
500
1000
1500
2000

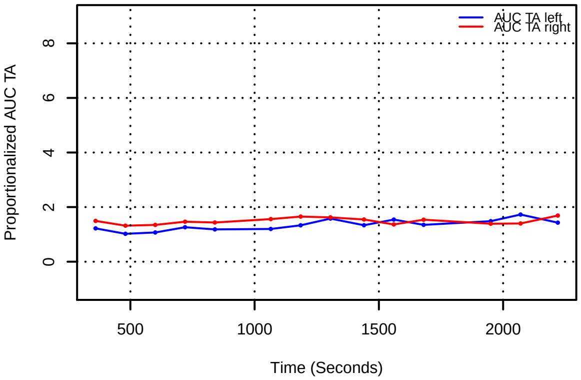
AUC TA left
AUC TA right
Proportionalized AUC TA
Time (Seconds)
0
2
4
6
8
500
1000
1500
2000

Patient 18

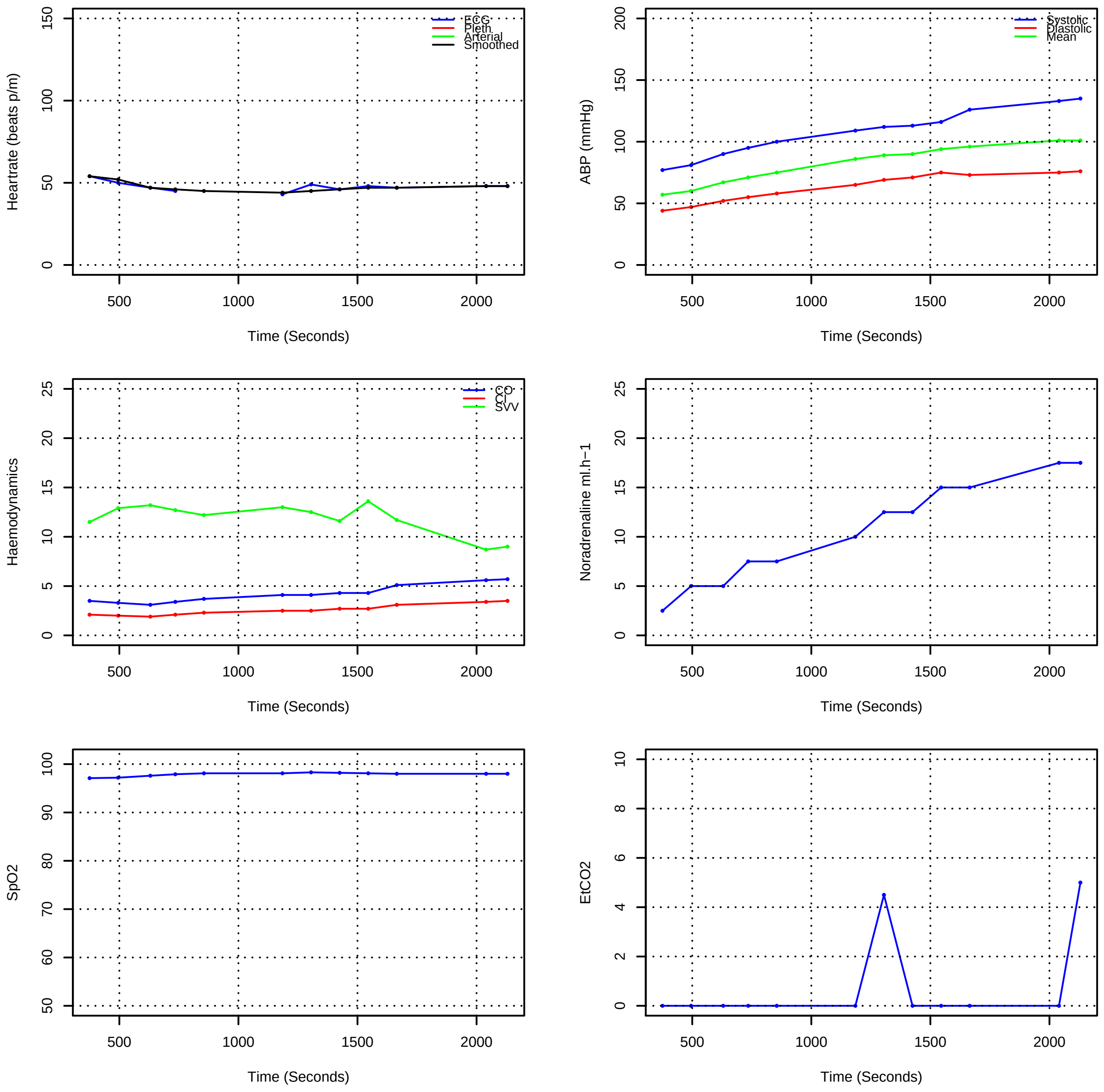

Heartrate (beats p/m)
ECG
Pleth
Arterial
Smoothed
ABP (mmHg)
Systolic
Diastolic
Mean
Haemodynamics
CO
CI
SVV
Noradrenaline ml.h−1
SpO2
EtCO2
Time (Seconds)

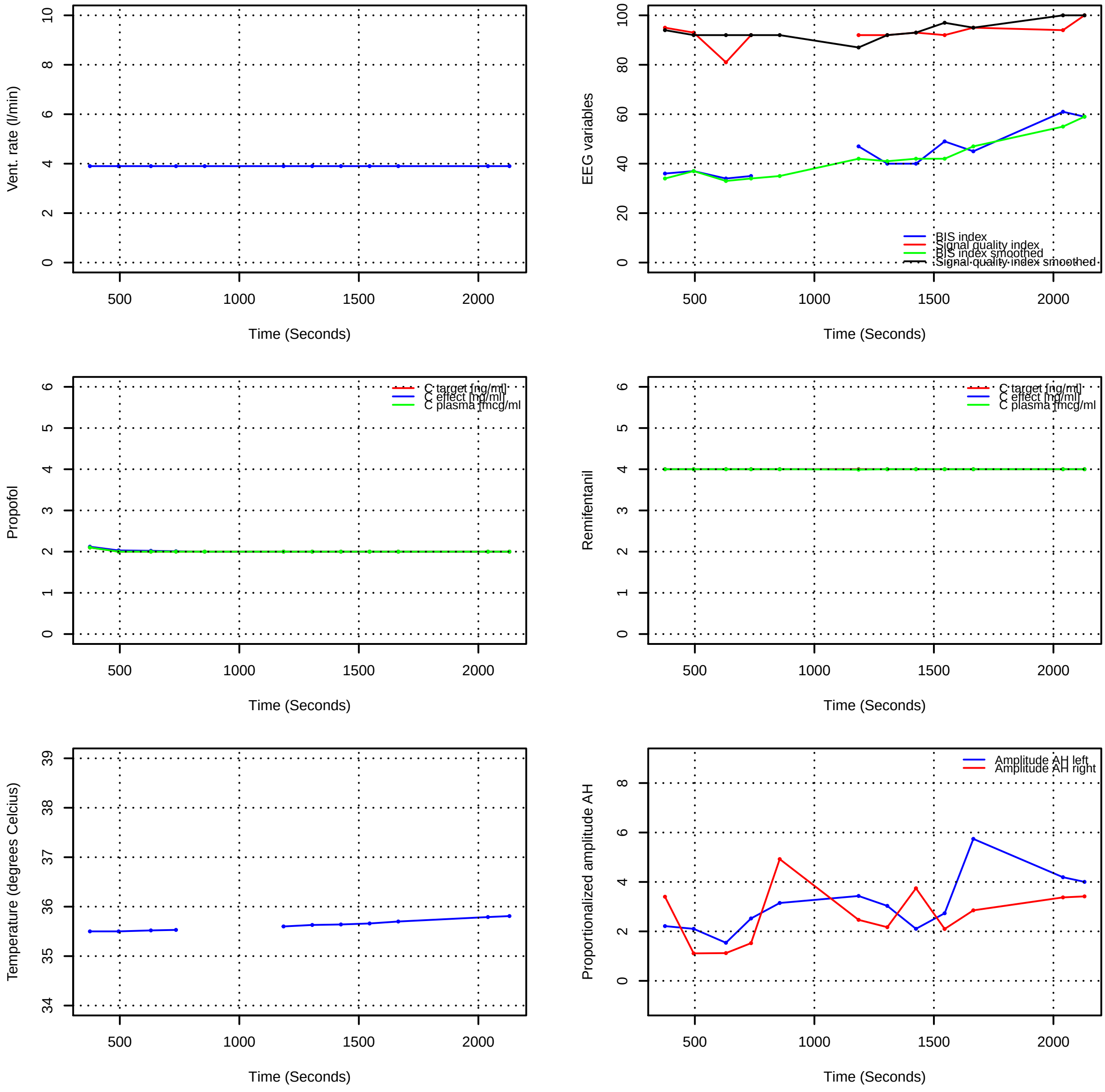

Vent. rate (l/min)
Time (Seconds)
EEG variables
BIS index
Signal quality index
BIS index smoothed
Signal quality index smoothed
Time (Seconds)
Propofol
C target [ng/ml]
C effect [ng/ml]
C plasma [mcg/ml
Time (Seconds)
Remifentanil
C target [ng/ml]
C effect [ng/ml]
C plasma [mcg/ml
Time (Seconds)
Temperature (degrees Celcius)
Time (Seconds)
Proportionalized amplitude AH
Amplitude AH left
Amplitude AH right
Time (Seconds)

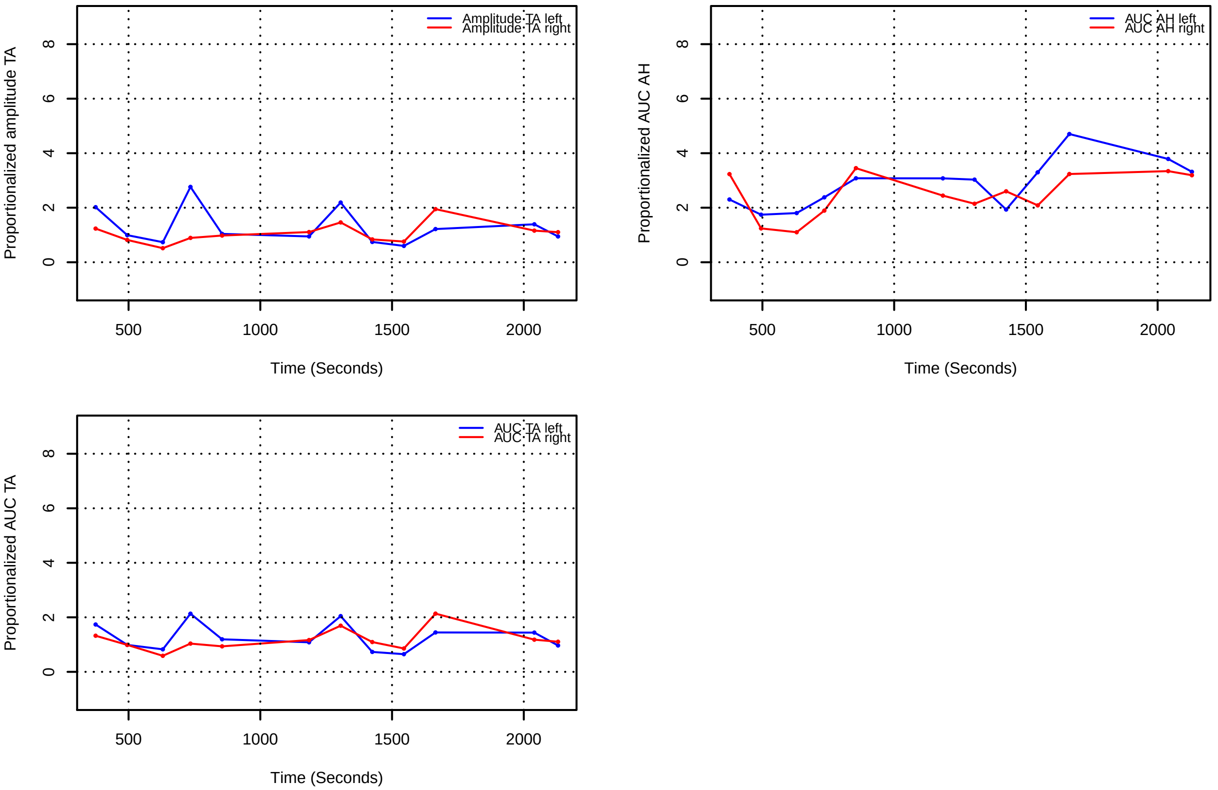
Amplitude TA left
Amplitude TA right
Proportionalized amplitude TA
Time (Seconds)
AUC AH left
AUC AH right
Proportionalized AUC AH
Time (Seconds)
AUC TA left
AUC TA right
Proportionalized AUC TA
Time (Seconds)
0
2
4
6
8
500
1000
1500
2000

Patient 19

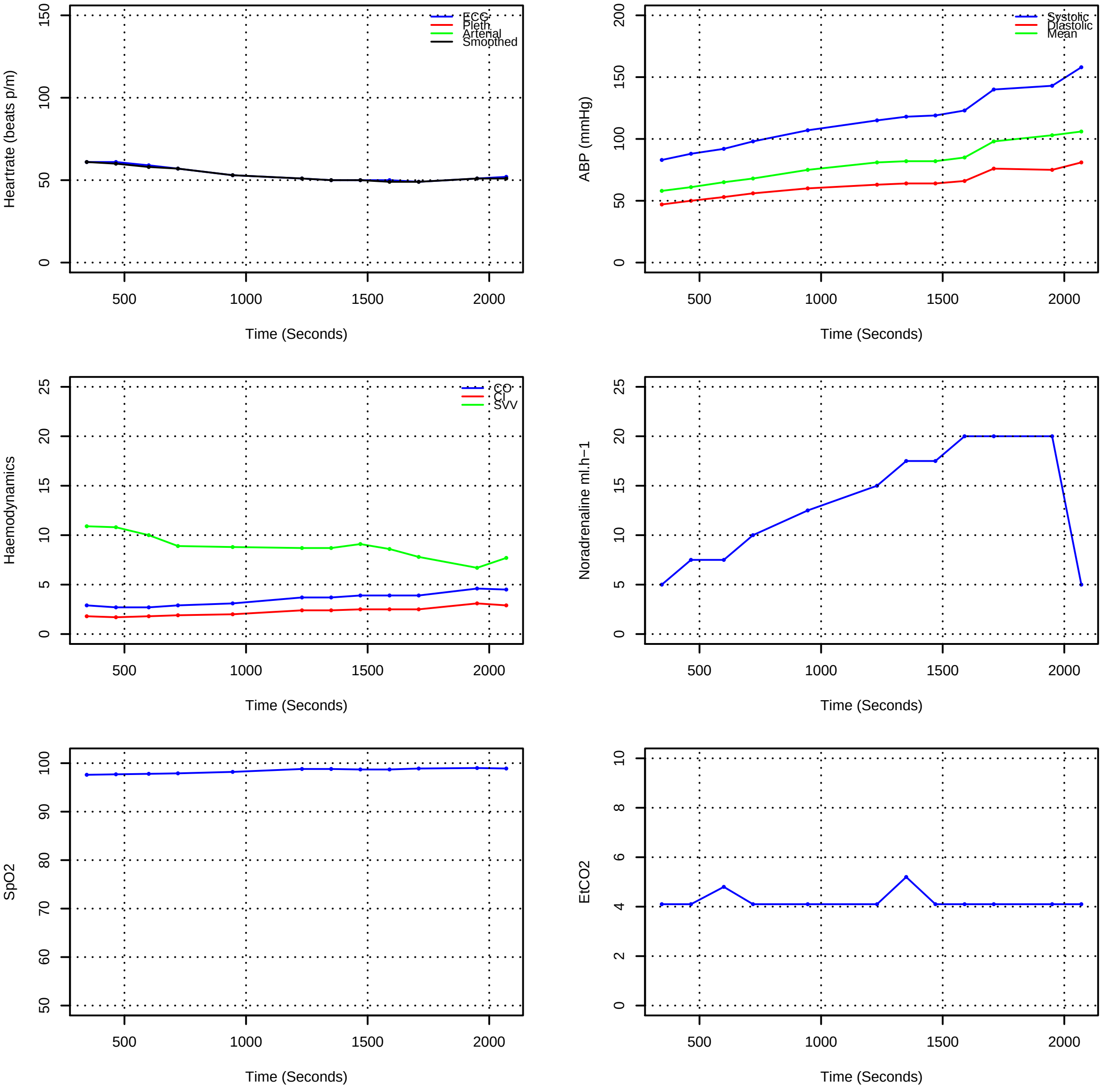

Heartrate (beats p/m)
ECG
Pleth
Arterial
Smoothed
Time (Seconds)
ABP (mmHg)
Systolic
Diastolic
Mean
Time (Seconds)
Haemodynamics
CO
CI
SVV
Time (Seconds)
Noradrenaline ml.h−1
Time (Seconds)
SpO2
Time (Seconds)
EtCO2
Time (Seconds)

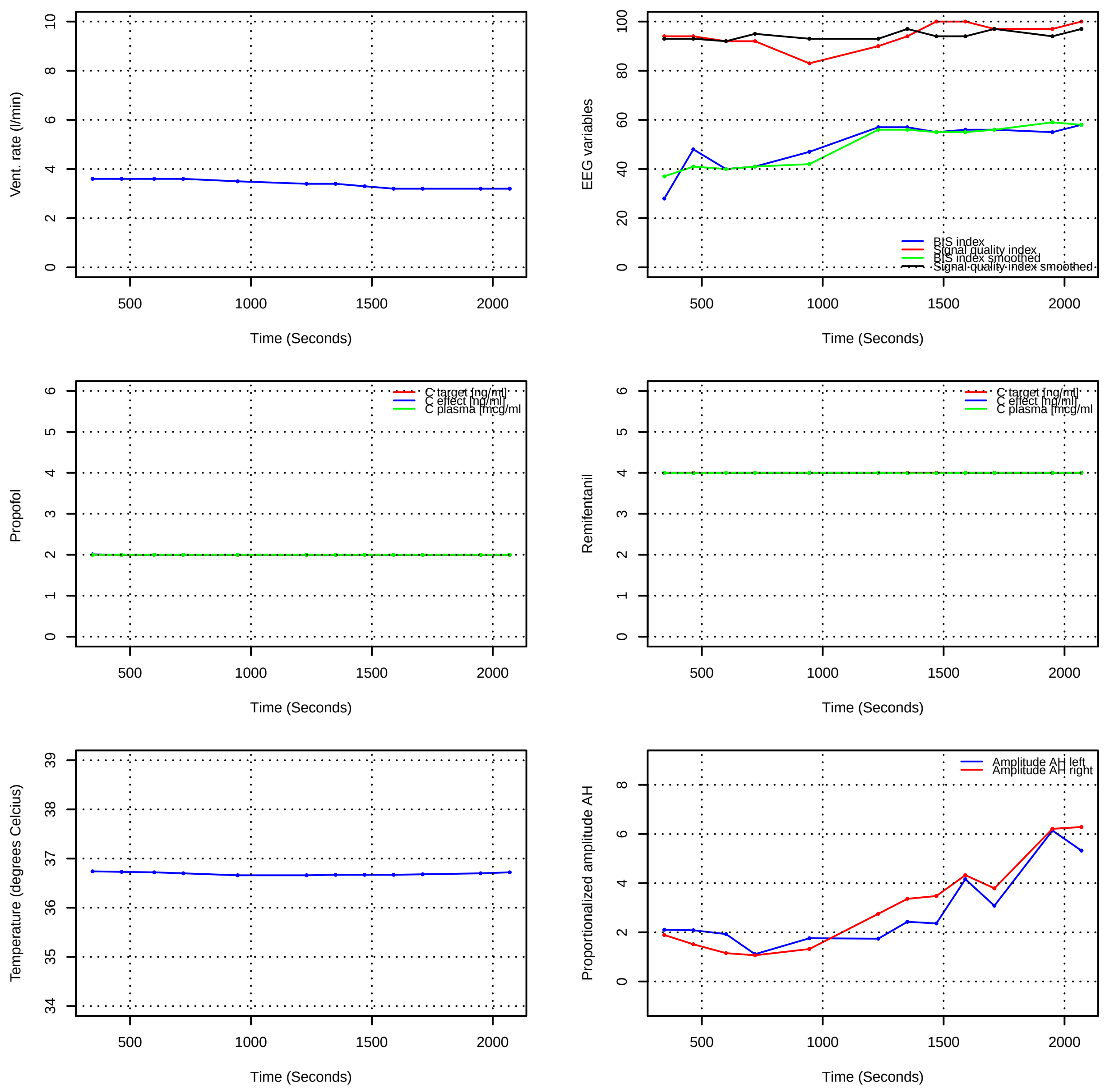

Vent. rate (l/min)
Time (Seconds)
EEG variables
BIS index
Signal quality index
BIS index smoothed
Signal quality index smoothed
Propofol
C target [ng/ml]
C effect [ng/ml]
C plasma [mcg/ml
Remifentanil
Temperature (degrees Celcius)
Proportionalized amplitude AH
Amplitude AH left
Amplitude AH right

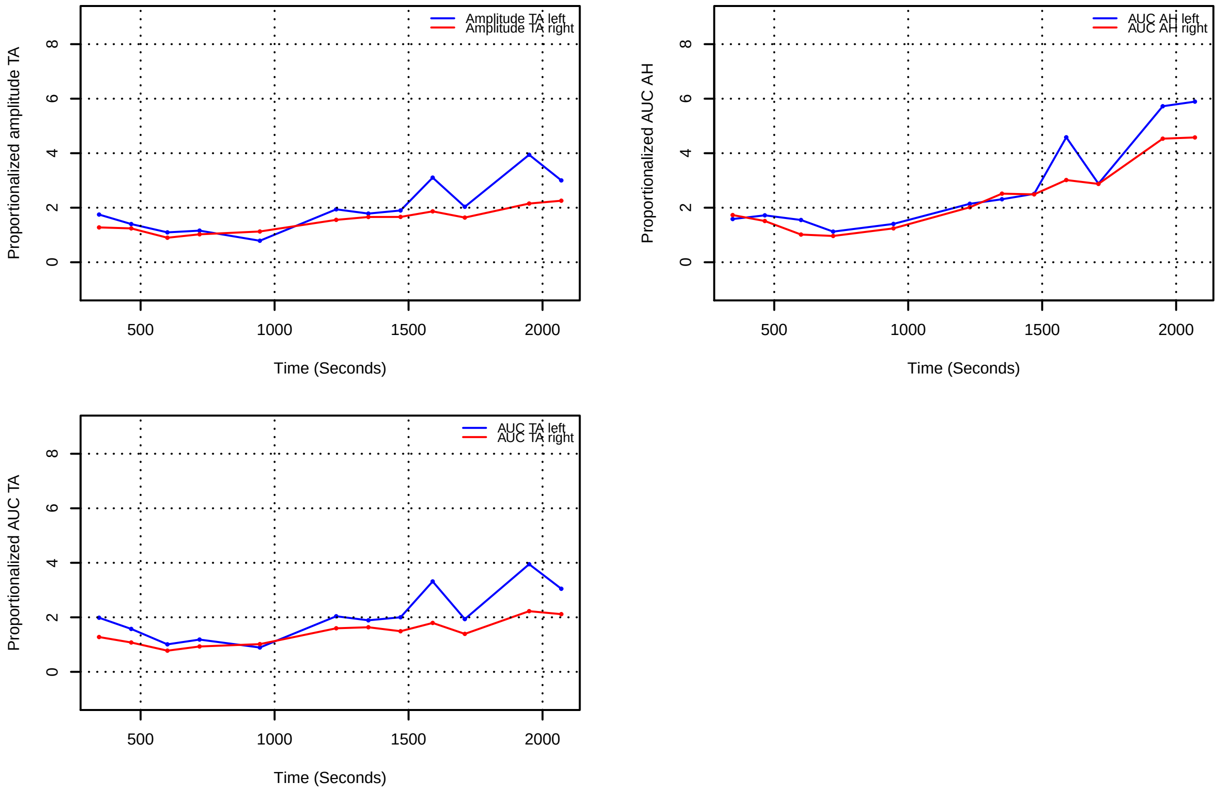
Amplitude TA left
Amplitude TA right
Proportionalized amplitude TA
Time (Seconds)
AUC AH left
AUC AH right
Proportionalized AUC AH
Time (Seconds)
AUC TA left
AUC TA right
Proportionalized AUC TA
Time (Seconds)

Patient 20

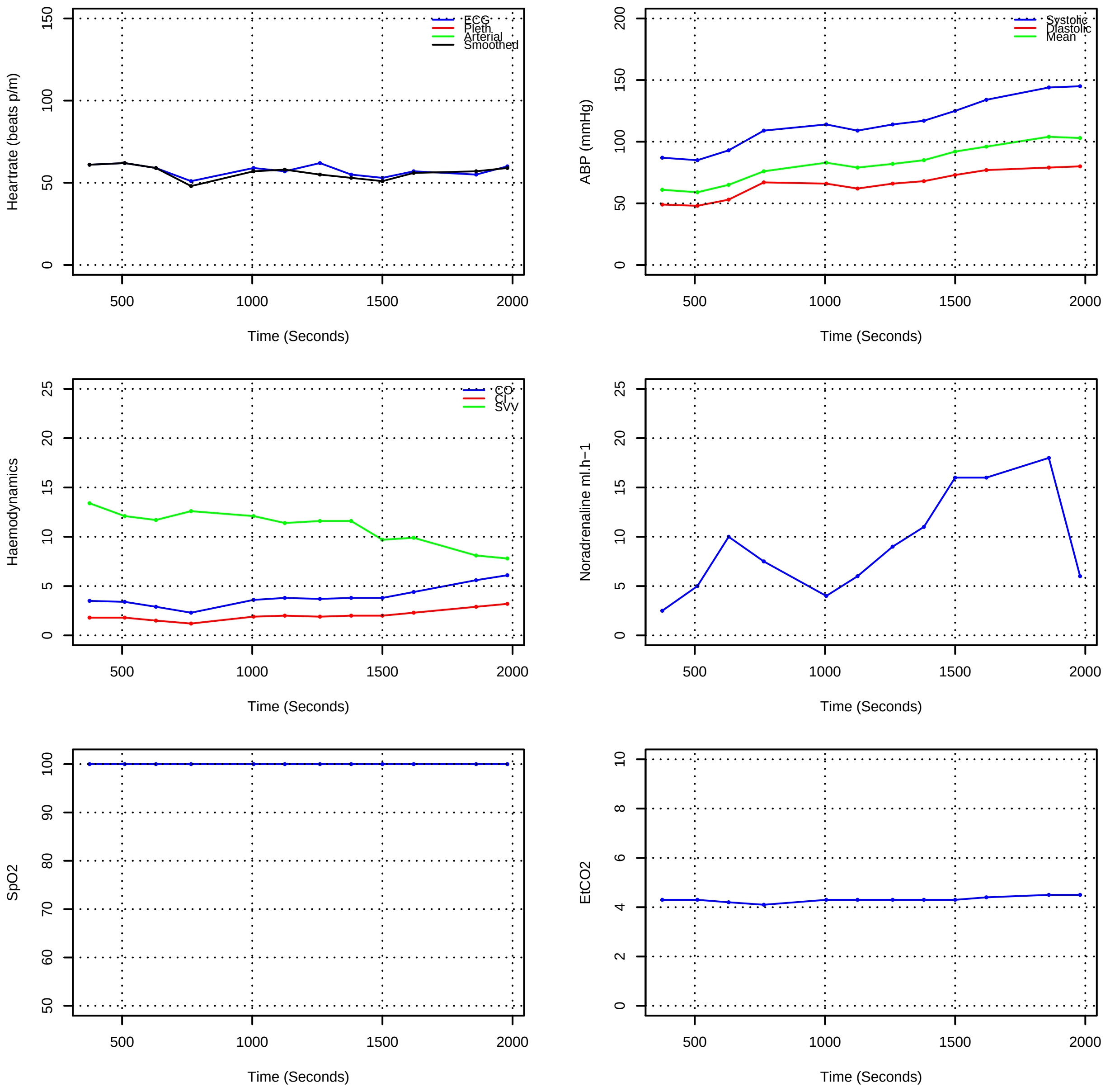
Heartrate (beats p/m)
ECG
Pleth
Arterial
Smoothed
ABP (mmHg)
Systolic
Diastolic
Mean
Haemodynamics
CO
CI
SVV
Noradrenaline ml.h−1
SpO2
EtCO2
Time (Seconds)

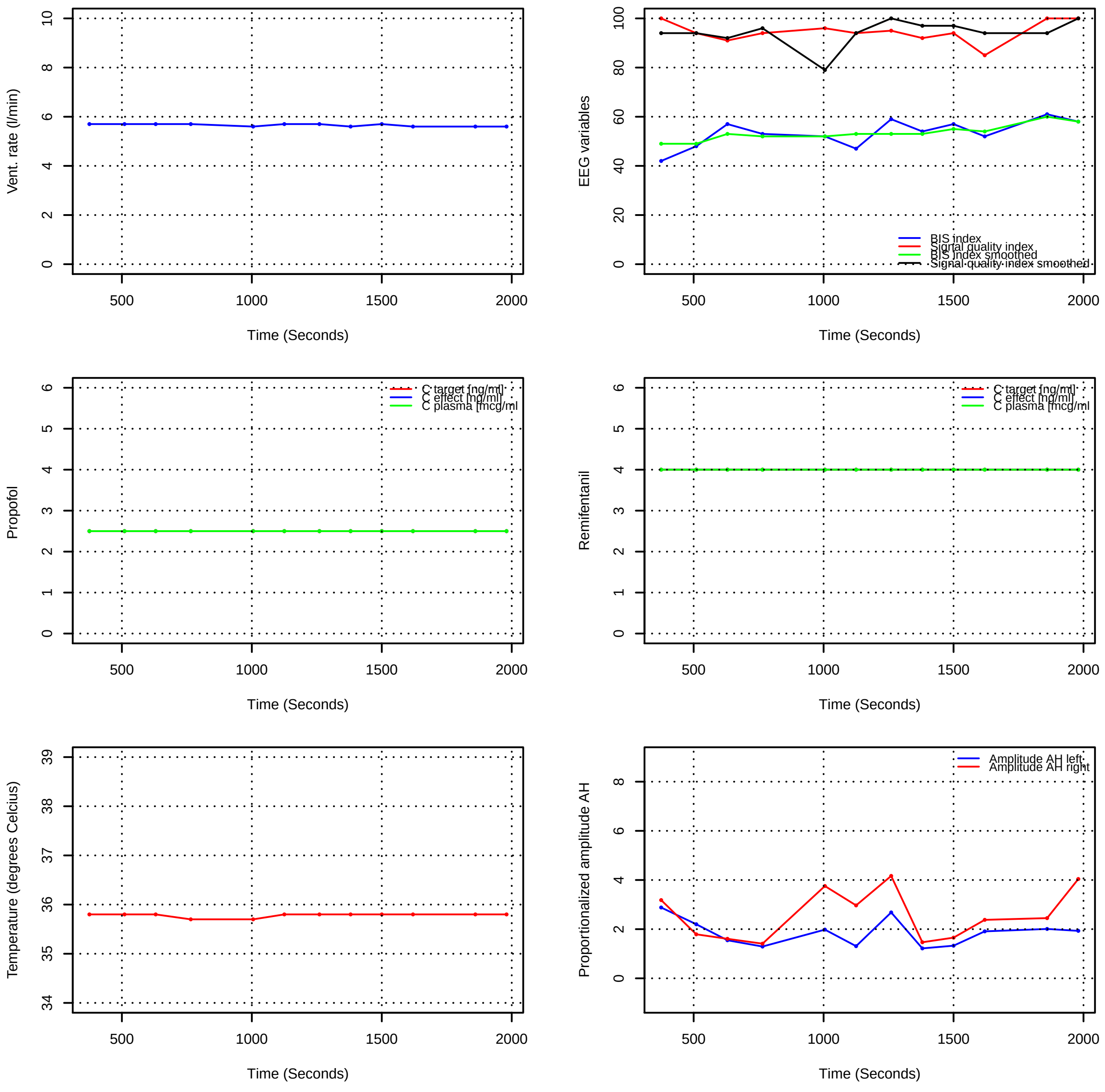
Vent. rate (l/min)
Time (Seconds)
EEG variables
BIS index
Signal quality index
BIS index smoothed
Signal quality index smoothed
Time (Seconds)
Propofol
C target [ng/ml]
C effect [ng/ml]
C plasma [mcg/ml
Time (Seconds)
Remifentanil
C target [ng/ml]
C effect [ng/ml]
C plasma [mcg/ml
Time (Seconds)
Temperature (degrees Celcius)
Time (Seconds)
Proportionalized amplitude AH
Amplitude AH left
Amplitude AH right
Time (Seconds)

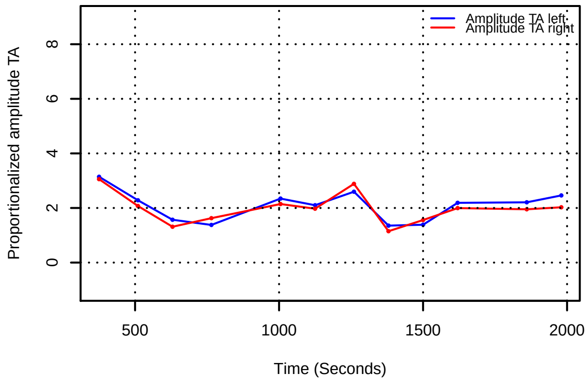

Amplitude TA left
Amplitude TA right
Proportionalized amplitude TA
0
2
4
6
8
500
1000
1500
2000
Time (Seconds)

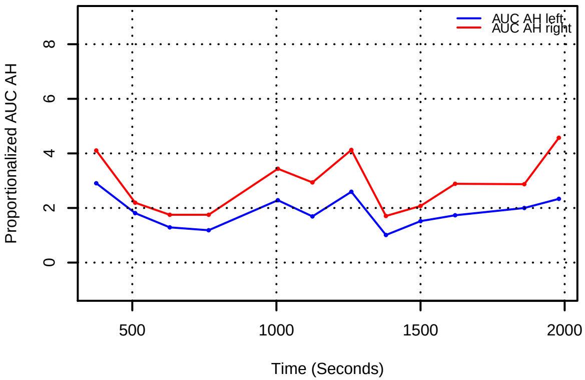

AUC AH left
AUC AH right
Proportionalized AUC AH
0
2
4
6
8
500
1000
1500
2000
Time (Seconds)

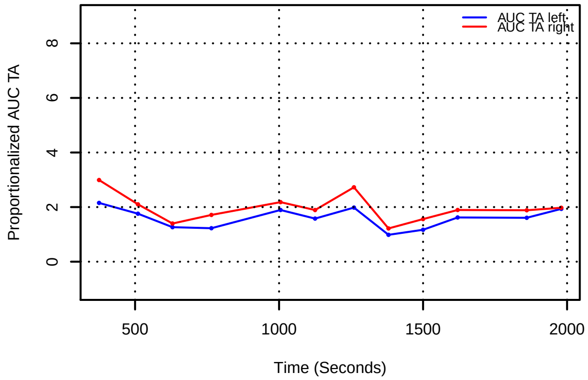

AUC TA left
AUC TA right
Proportionalized AUC TA
0
2
4
6
8
500
1000
1500
2000
Time (Seconds)

Patient 21

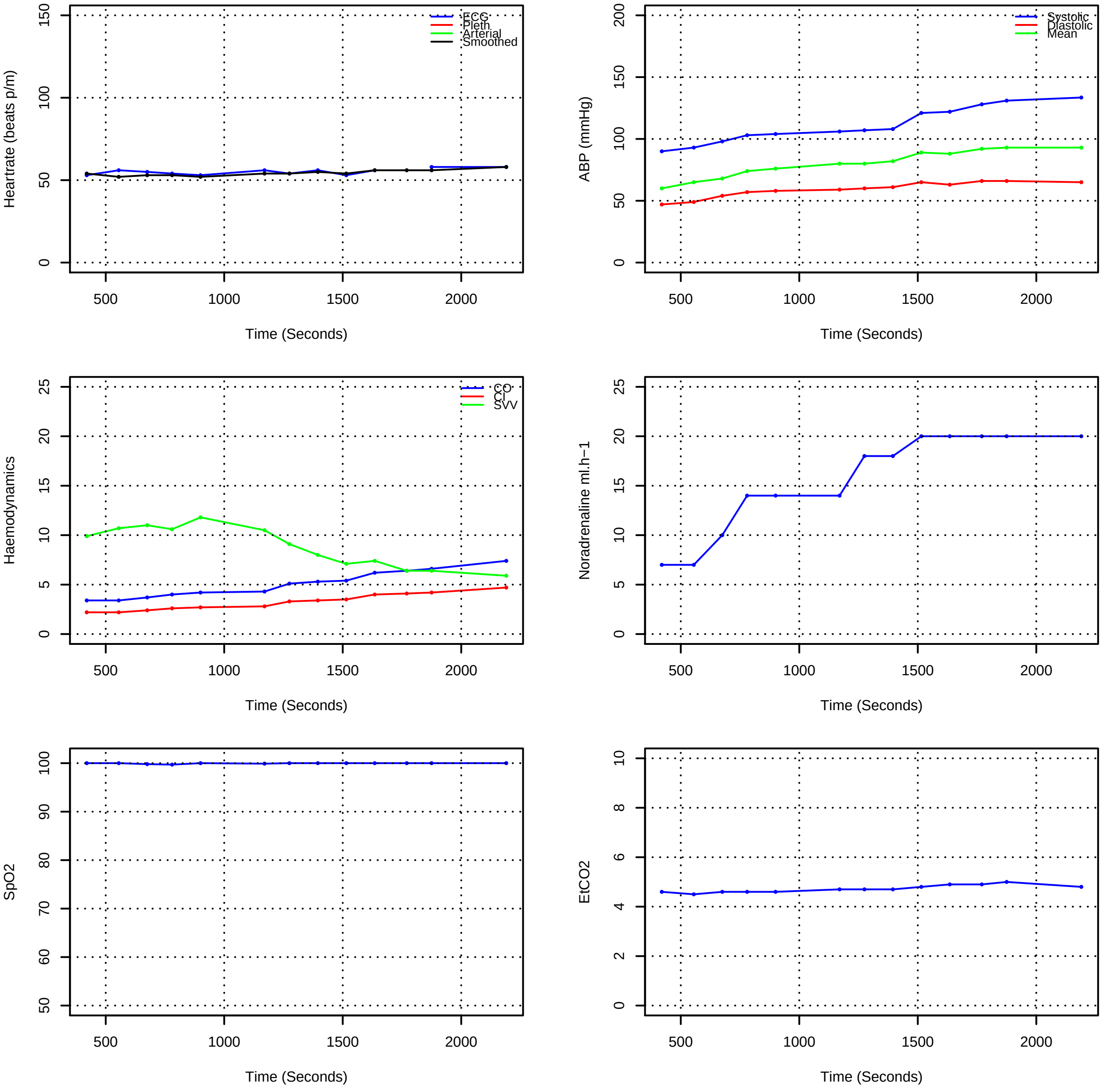
Heartrate (beats p/m)
Time (Seconds)
ECG
Pleth
Arterial
Smoothed
ABP (mmHg)
Time (Seconds)
Systolic
Diastolic
Mean
Haemodynamics
Time (Seconds)
CO
CI
SVV
Noradrenaline ml.h−1
Time (Seconds)
SpO2
Time (Seconds)
EtCO2
Time (Seconds)

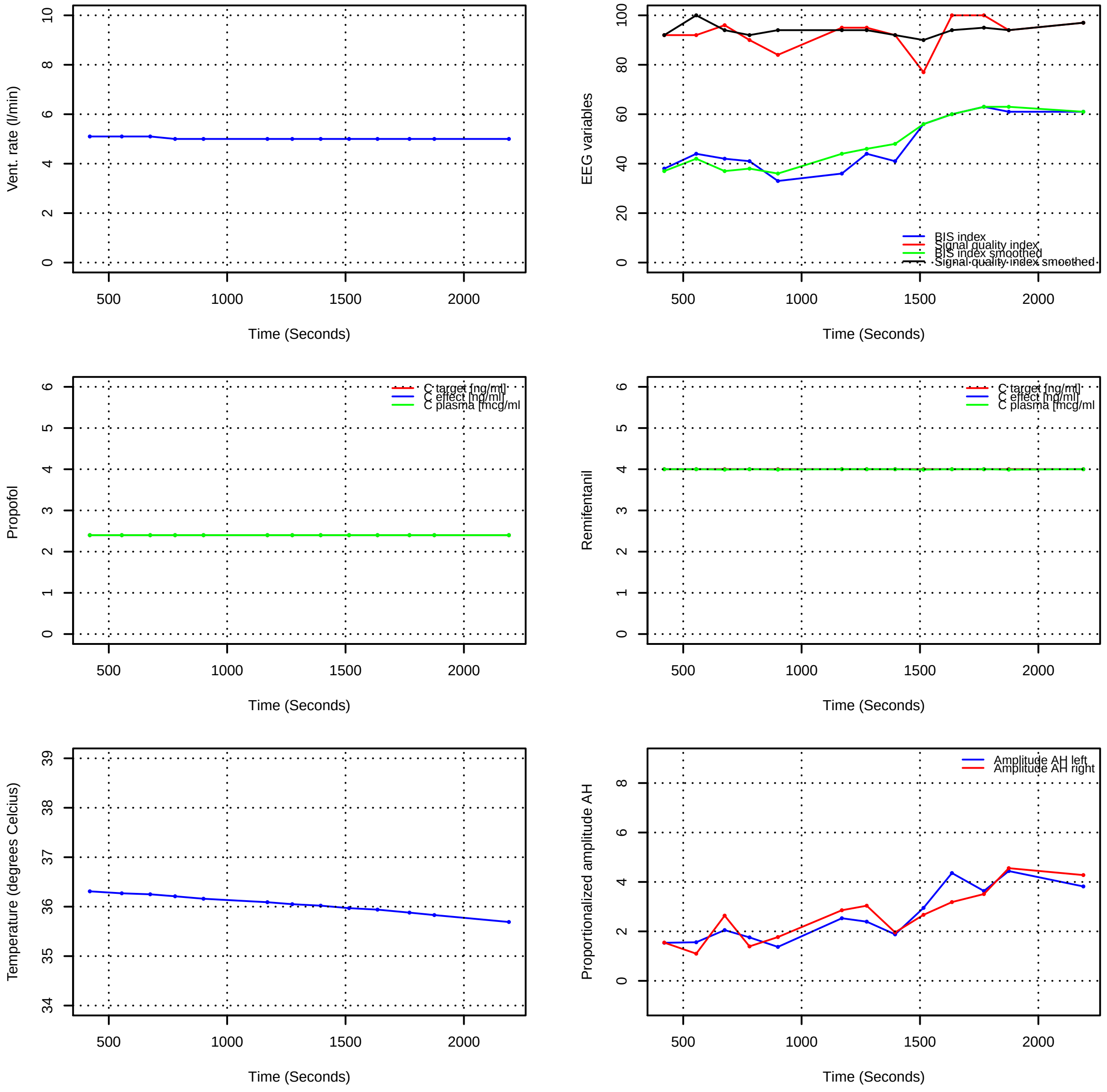

Vent. rate (l/min)
Time (Seconds)
EEG variables
BIS index
Signal quality index
BIS index smoothed
Signal quality index smoothed
Time (Seconds)
Propofol
C target [ng/ml]
C effect [ng/ml]
C plasma [mcg/ml
Time (Seconds)
Remifentanil
C target [ng/ml]
C effect [ng/ml]
C plasma [mcg/ml
Time (Seconds)
Temperature (degrees Celcius)
Time (Seconds)
Proportionalized amplitude AH
Amplitude AH left
Amplitude AH right
Time (Seconds)

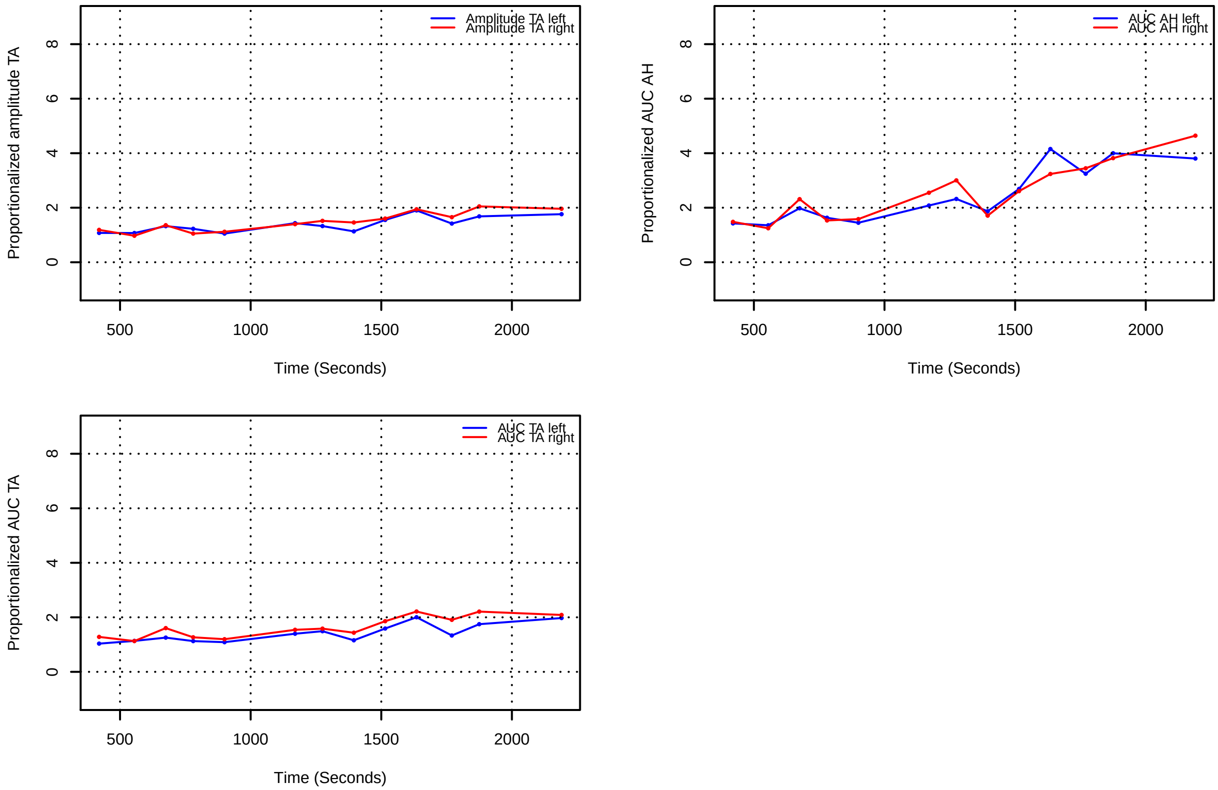
Amplitude TA left
Amplitude TA right
Proportionalized amplitude TA
Time (Seconds)
AUC AH left
AUC AH right
Proportionalized AUC AH
Time (Seconds)
AUC TA left
AUC TA right
Proportionalized AUC TA
Time (Seconds)
0
2
4
6
8
500
1000
1500
2000

Patient 22

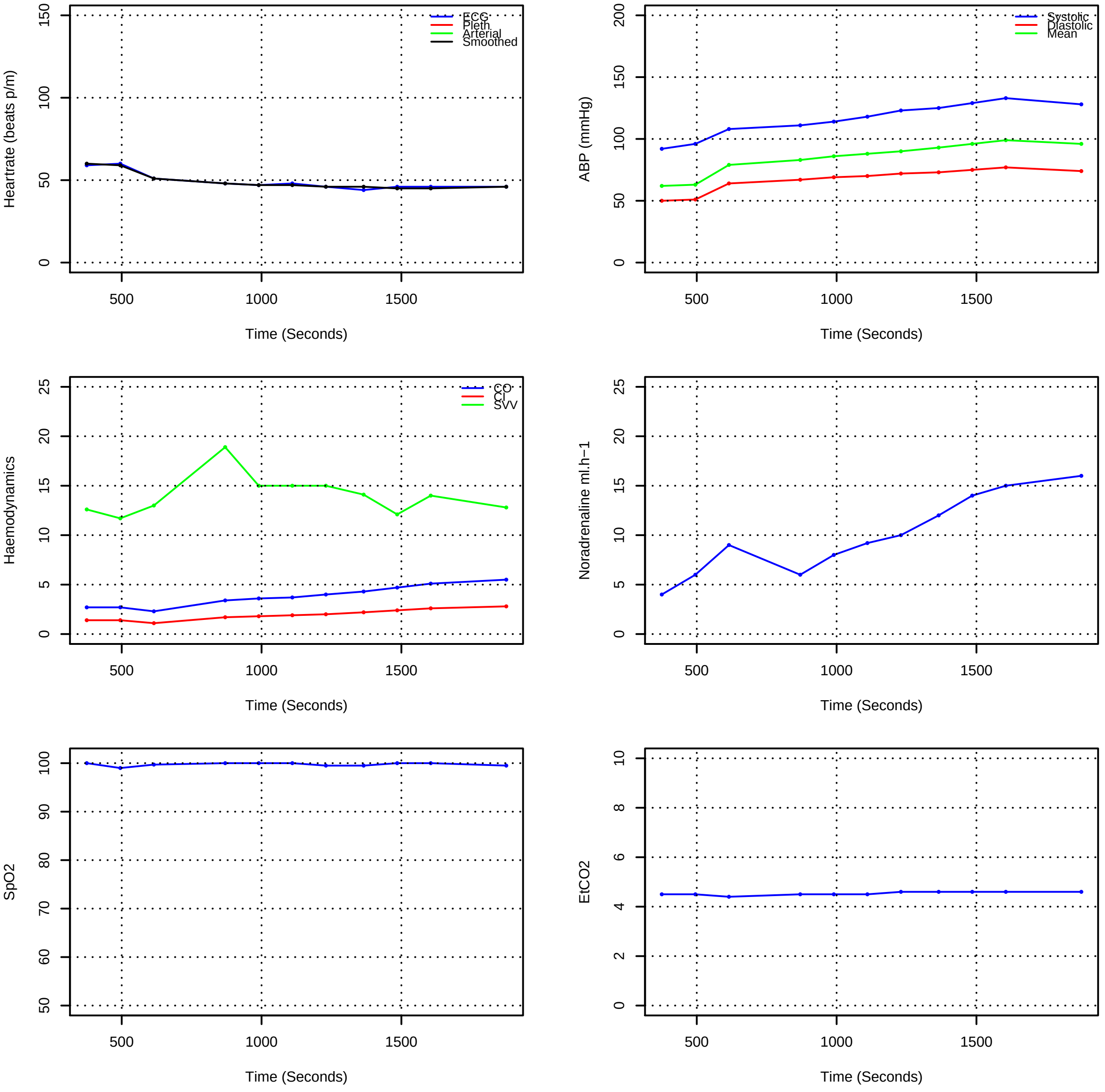

Heartrate (beats p/m)
ECG
Pleth
Arterial
Smoothed
ABP (mmHg)
Systolic
Diastolic
Mean
Haemodynamics
CO
CI
SVV
Noradrenaline ml.h−1
SpO2
EtCO2
Time (Seconds)

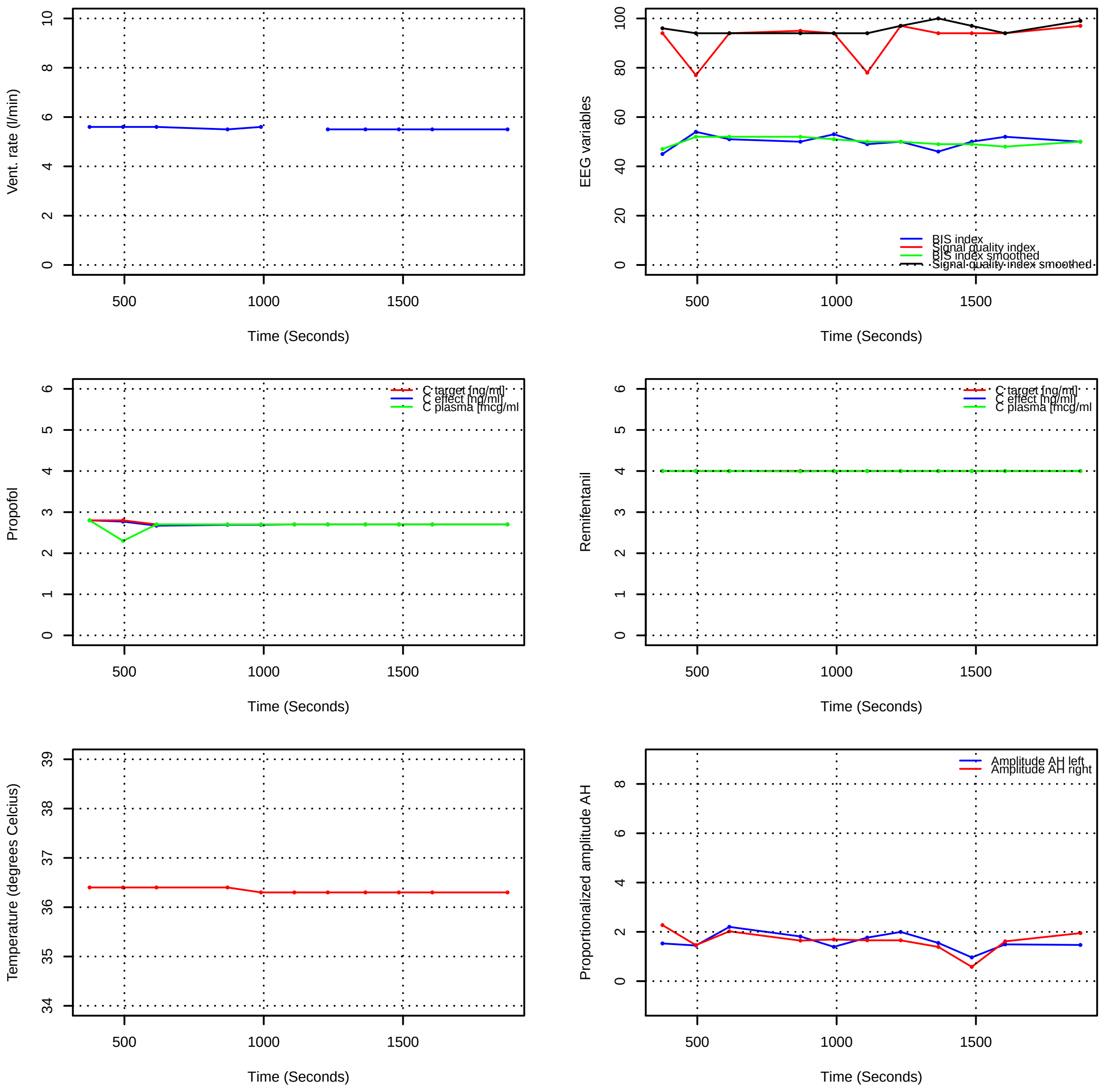
Vent. rate (l/min)
0
2
4
6
8
10
500
1000
1500
Time (Seconds)
EEG variables
0
20
40
60
80
100
BIS index
Signal quality index
BIS index smoothed
Signal quality index smoothed
500
1000
1500
Time (Seconds)
Propofol
0
1
2
3
4
5
6
C target [ng/ml]
C effect [ng/ml]
C plasma [mcg/ml
500
1000
1500
Time (Seconds)
Remifentanil
0
1
2
3
4
5
6
C target [ng/ml]
C effect [ng/ml]
C plasma [mcg/ml
500
1000
1500
Time (Seconds)
Temperature (degrees Celcius)
34
35
36
37
38
39
500
1000
1500
Time (Seconds)
Proportionalized amplitude AH
0
2
4
6
8
Amplitude AH left
Amplitude AH right
500
1000
1500
Time (Seconds)

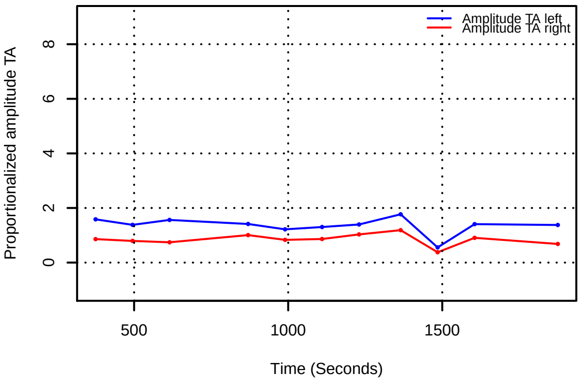

Amplitude TA left
Amplitude TA right
Proportionalized amplitude TA
0
2
4
6
8
500
1000
1500
Time (Seconds)

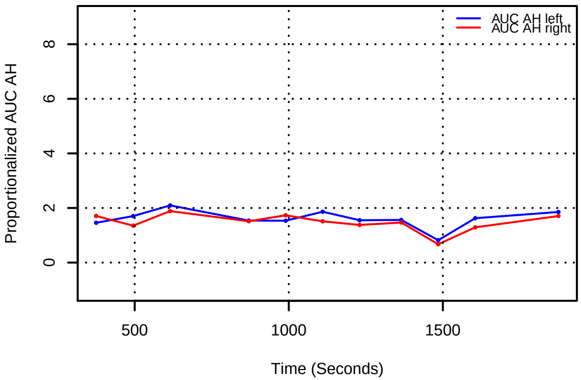

AUC AH left
AUC AH right
Proportionalized AUC AH
0
2
4
6
8
500
1000
1500
Time (Seconds)

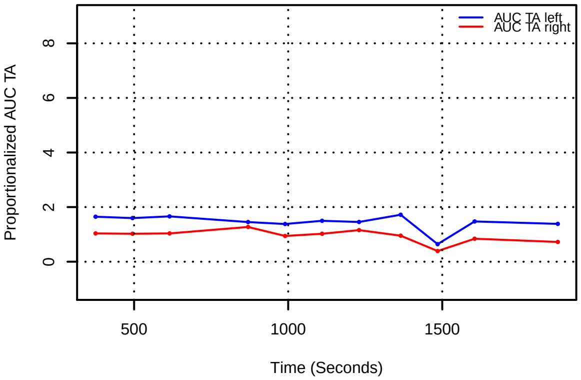

AUC TA left
AUC TA right
Proportionalized AUC TA
0
2
4
6
8
500
1000
1500
Time (Seconds)

Patient 23

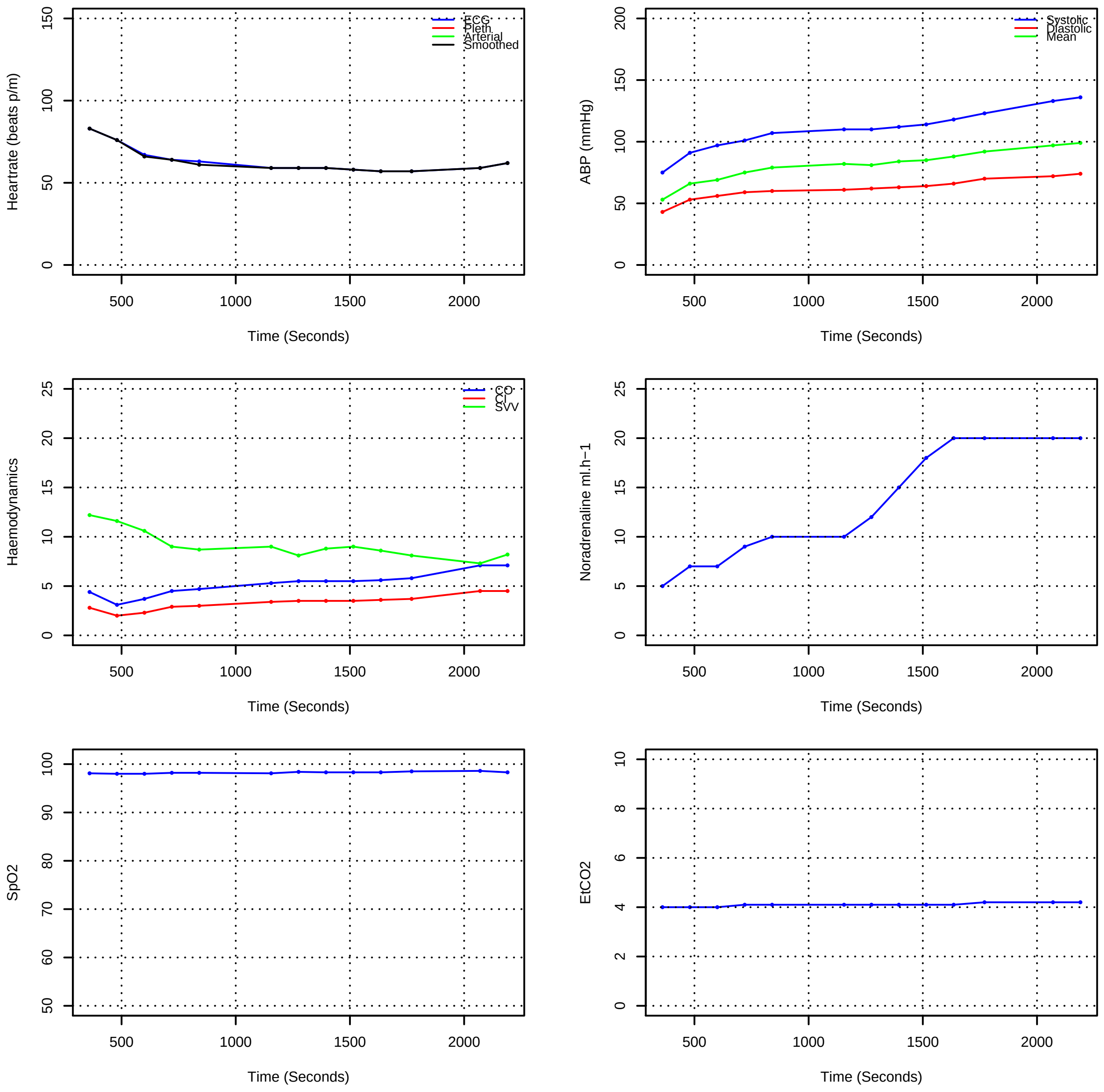

ECG
Pleth
Arterial
Smoothed
Heartrate (beats p/m)
Time (Seconds)
Systolic
Diastolic
Mean
ABP (mmHg)
Time (Seconds)
CO
CI
SVV
Haemodynamics
Time (Seconds)
Noradrenaline ml.h−1
Time (Seconds)
SpO2
Time (Seconds)
EtCO2
Time (Seconds)

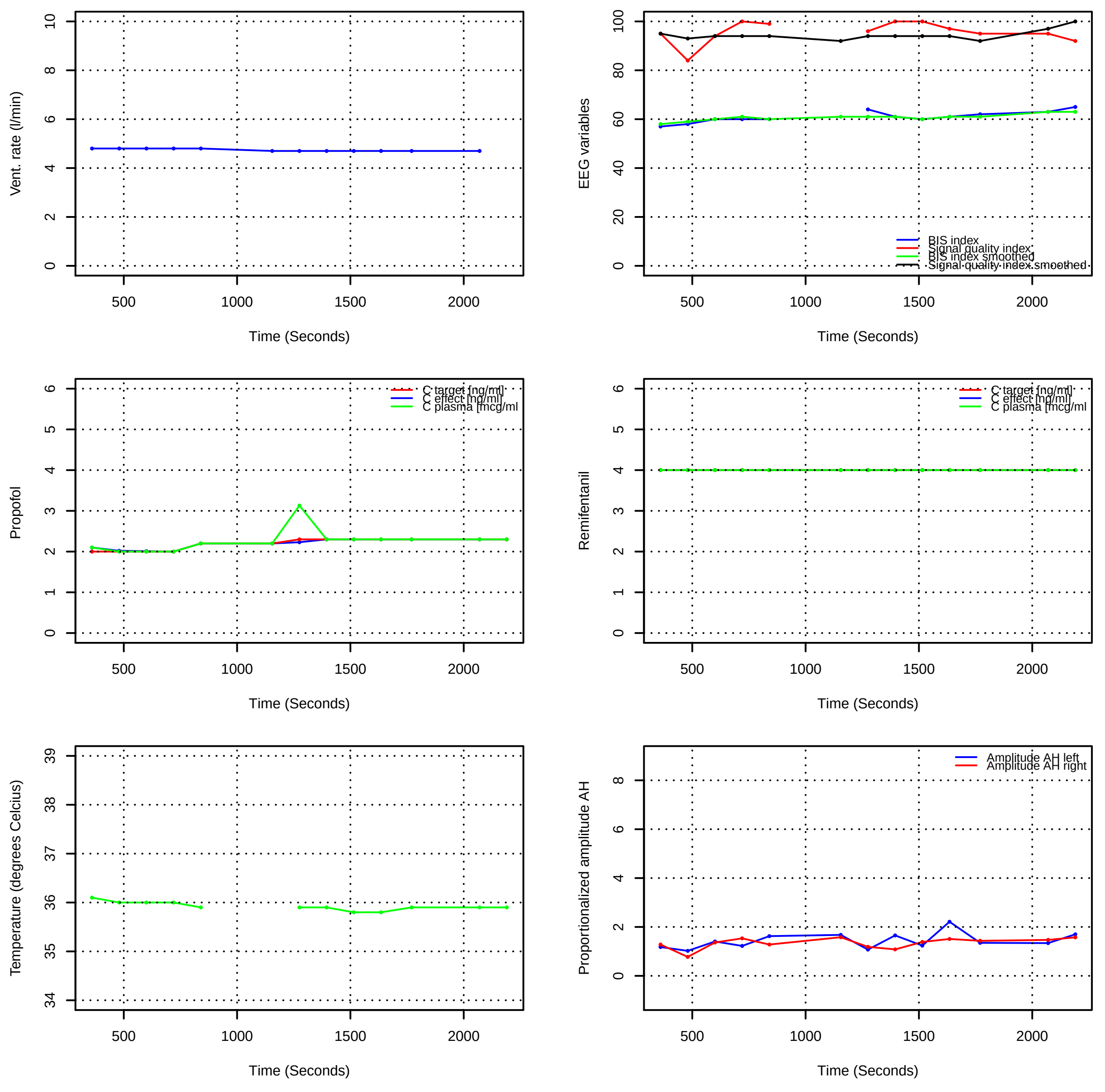

Vent. rate (l/min)
Time (Seconds)
EEG variables
BIS index
Signal quality index
BIS index smoothed
Signal quality index smoothed
Propofol
C target [ng/ml]
C effect [ng/ml]
C plasma [mcg/ml
Remifentanil
Temperature (degrees Celcius)
Proportionalized amplitude AH
Amplitude AH left
Amplitude AH right

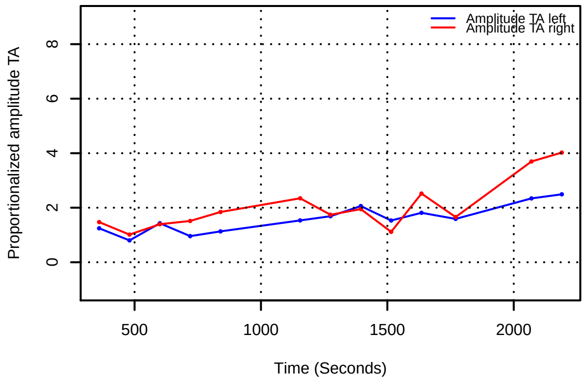
Amplitude TA left
Amplitude TA right
Proportionalized amplitude TA
Time (Seconds)
0
2
4
6
8
500
1000
1500
2000

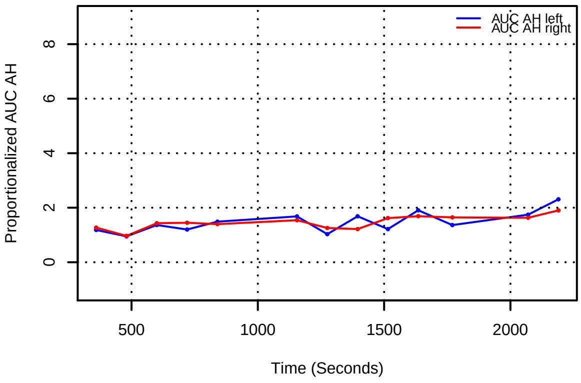
AUC AH left
AUC AH right
Proportionalized AUC AH
Time (Seconds)
0
2
4
6
8
500
1000
1500
2000

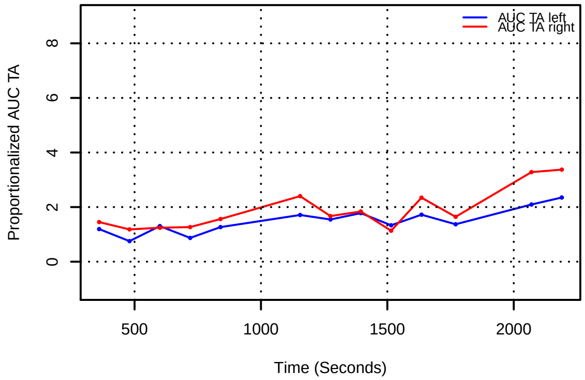
AUC TA left
AUC TA right
Proportionalized AUC TA
Time (Seconds)
0
2
4
6
8
500
1000
1500
2000

Patient 24

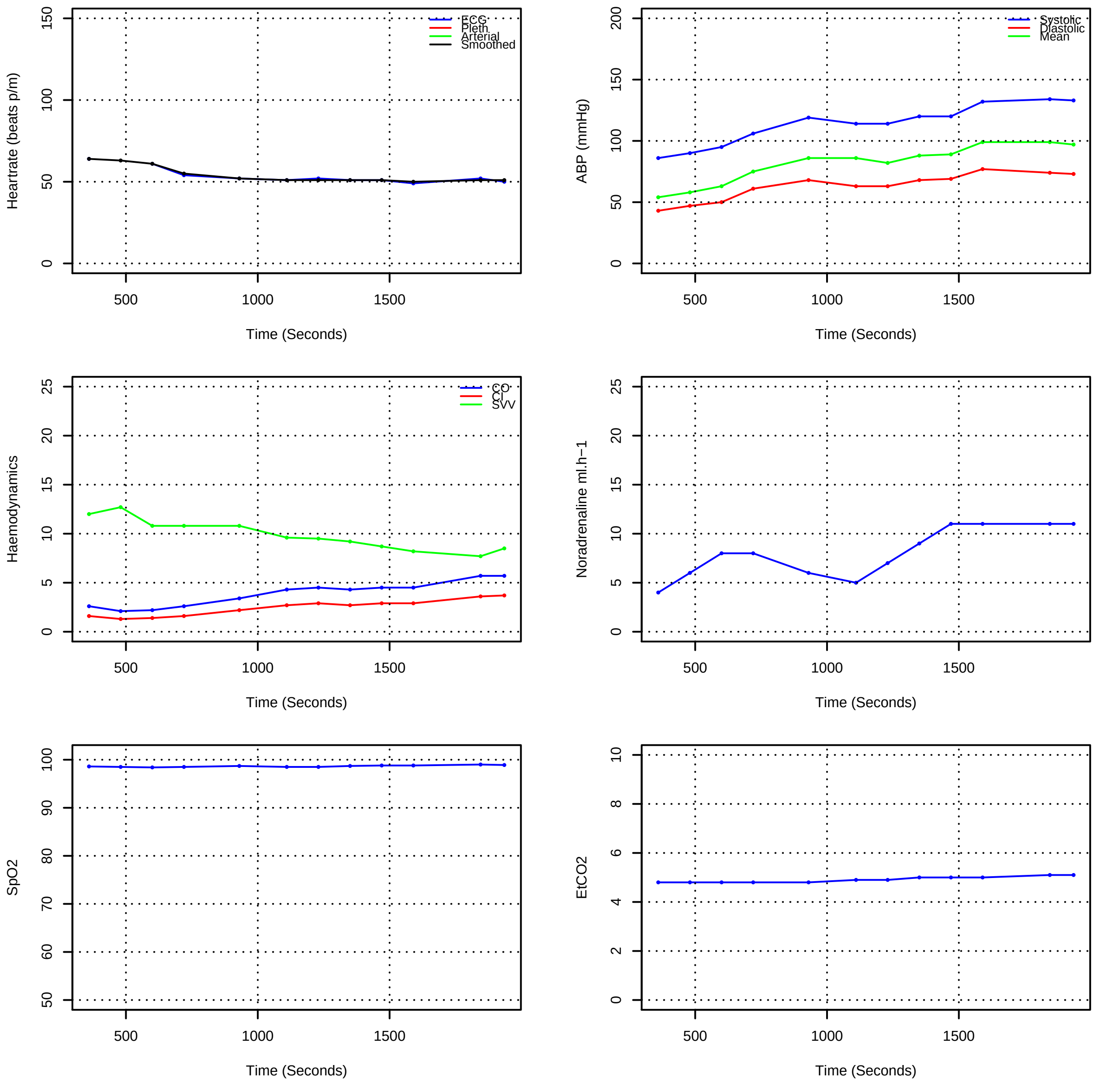
Heartrate (beats p/m)
Time (Seconds)
ECG
Pleth
Arterial
Smoothed
ABP (mmHg)
Time (Seconds)
Systolic
Diastolic
Mean
Haemodynamics
Time (Seconds)
CO
CI
SVV
Noradrenaline ml.h−1
Time (Seconds)
SpO2
Time (Seconds)
EtCO2
Time (Seconds)

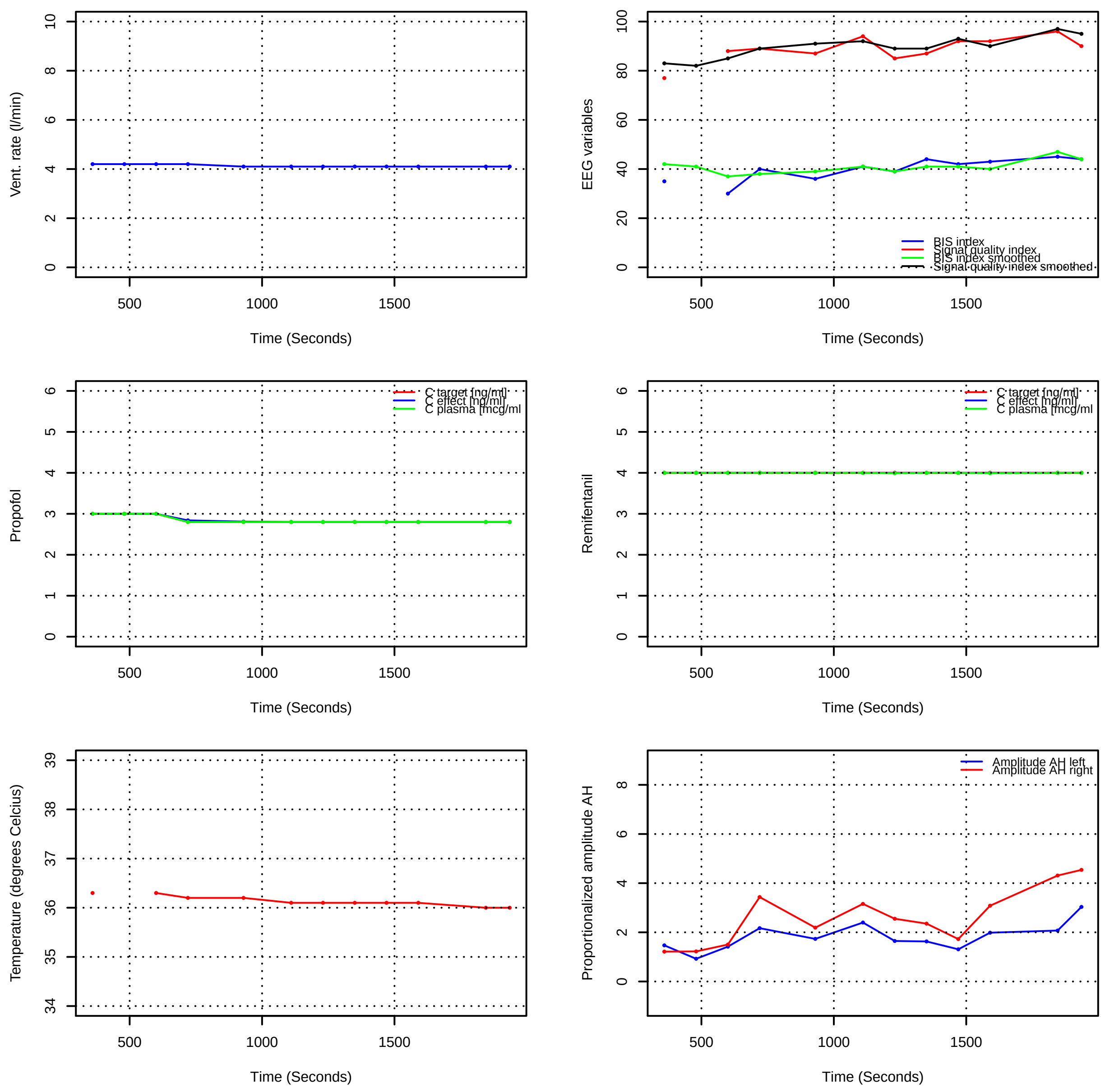
Vent. rate (l/min)
Time (Seconds)
EEG variables
BIS index
Signal quality index
BIS index smoothed
Signal quality index smoothed
Time (Seconds)
Propofol
C target [ng/ml]
C effect [ng/ml]
C plasma [mcg/ml
Time (Seconds)
Remifentanil
C target [ng/ml]
C effect [ng/ml]
C plasma [mcg/ml
Time (Seconds)
Temperature (degrees Celcius)
Time (Seconds)
Proportionalized amplitude AH
Amplitude AH left
Amplitude AH right
Time (Seconds)

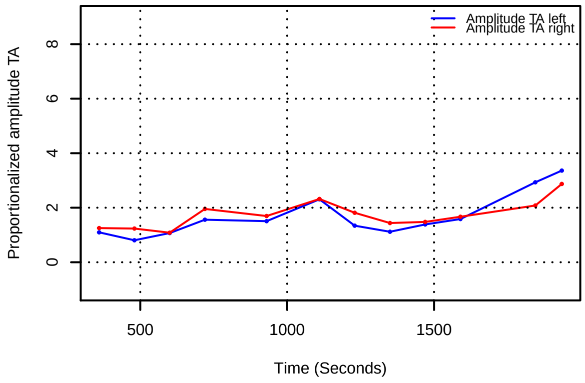

Amplitude TA left
Amplitude TA right
Proportionalized amplitude TA
Time (Seconds)

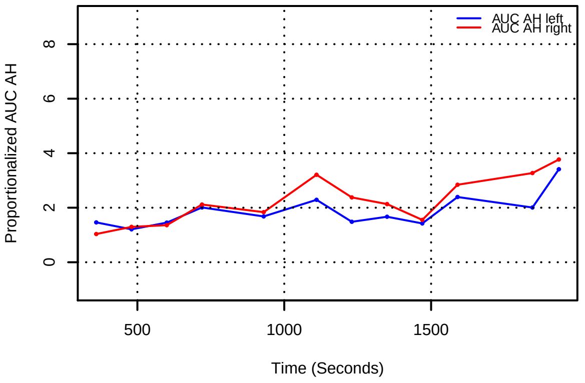

AUC AH left
AUC AH right
Proportionalized AUC AH
Time (Seconds)

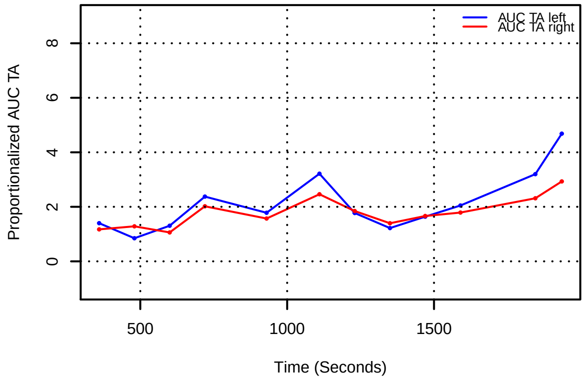

AUC TA left
AUC TA right
Proportionalized AUC TA
Time (Seconds)

Patient 25

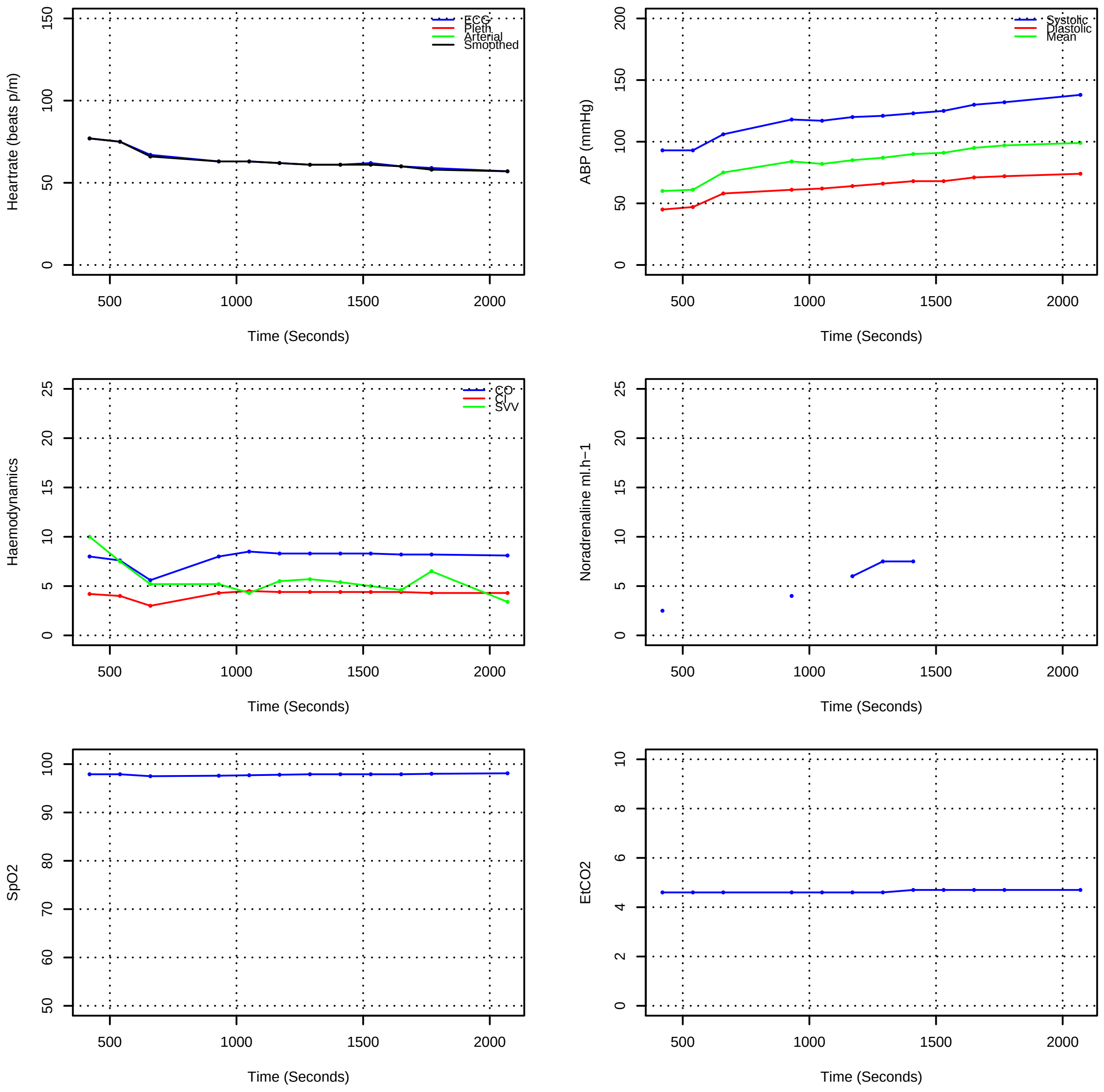

Heartrate (beats p/m)
0
50
100
150
500
1000
1500
2000
Time (Seconds)
ECG
Pleth
Arterial
Smoothed
ABP (mmHg)
0
50
100
150
200
Systolic
Diastolic
Mean
Haemodynamics
0
5
10
15
20
25
CO
CI
SVV
Noradrenaline ml.h−1
SpO2
50
60
70
80
90
100
EtCO2
0
2
4
6
8
10

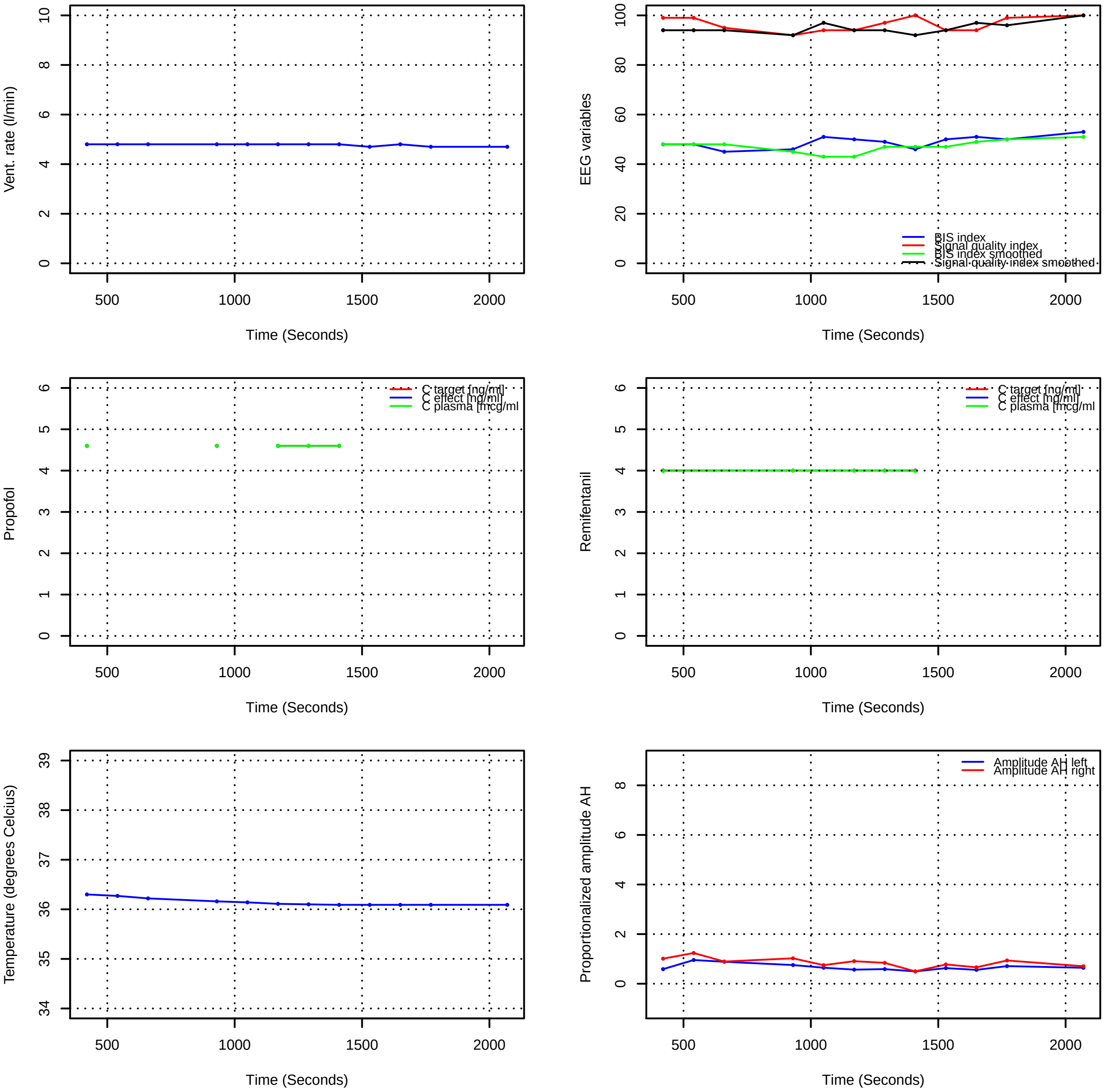

Vent. rate (l/min)
Time (Seconds)
EEG variables
BIS index
Signal quality index
BIS index smoothed
Signal quality index smoothed
Time (Seconds)
Propofol
C target [ng/ml]
C effect [ng/ml]
C plasma [mcg/ml
Time (Seconds)
Remifentanil
C target [ng/ml]
C effect [ng/ml]
C plasma [mcg/ml
Time (Seconds)
Temperature (degrees Celcius)
Time (Seconds)
Proportionalized amplitude AH
Amplitude AH left
Amplitude AH right
Time (Seconds)

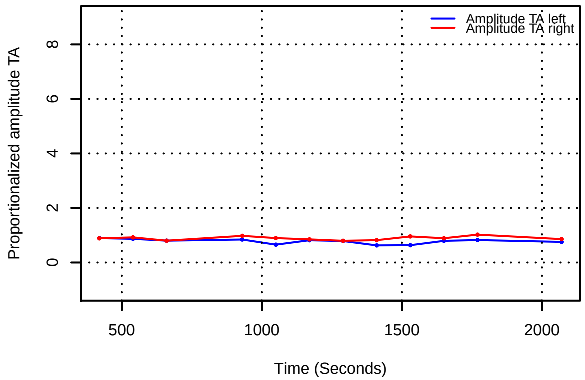
Amplitude TA left
Amplitude TA right
Proportionalized amplitude TA
Time (Seconds)

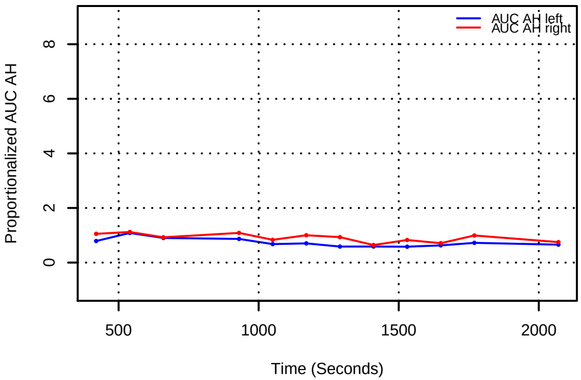
AUC AH left
AUC AH right
Proportionalized AUC AH
Time (Seconds)

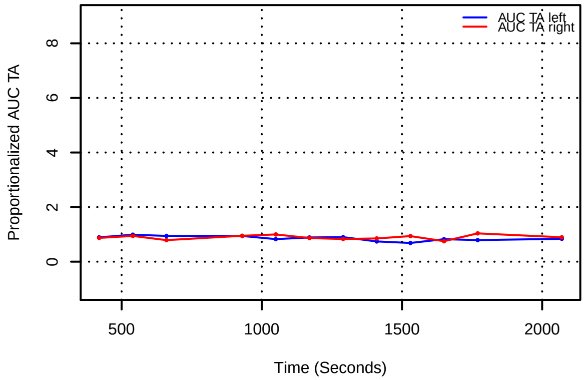
AUC TA left
AUC TA right
Proportionalized AUC TA
Time (Seconds)
