# Supplementary material for: The Influence of Vasopressor-Induced Arterial Blood Pressure Elevation on Muscle-Recorded Motor Evoked Potentials
Source: Anesth Analg. 2025 Sep 5;142(4):730–40. doi: 10.1213/ANE.0000000000007701 (PMC12959597; doi:10.1213/ANE.0000000000007701)
Supplement: Supplementary file 2 [file ane-142-730-s002.pdf]

Supplementary table 1. Results of the mixed effects model analyses for the effects of cardiac index on mTc-MEP amplitude and AUC adjusted for BIS, estimated plasma propofol concentration and ephedrine

Outcome		Coefficient	95% CI	p-value
**Amplitude*** **(proportion of baseline)**	CI (L.min.m^2)	17.07	-1.11 - 35.24	0.066
**AUC*** **(proportion of baseline)**	CI (L.min.m^2)	16.39	-1.45 - 34.23	0.072

CI, cardiac index; AUC, area under the curve;
* Corrected for side, muscle, BIS, estimated plasma propofol concentration and ephedrine. Patient was added as random intercept and CI was added as random slope.
Interaction term between CI and muscle was not significant
The estimates for amplitude and AUC are percentages (1 L.min.m^2 gives 17.07% in mTc-MEP amplitude).
Significant coefficients are shown in bold text.

Supplementary table 2. Results of the mixed effects model analyses. H-reflex and CMAP post-hoc analysis

		**Primary objective**			**Secondary objective**		
**Outcome**		**Coefficient**	**95% CI**	**p-value**	**Coefficient**	**95% CI**	**p-value**
**H-reflex amplitude** **(Proportion of baseline)**	MAP (mmHg)	0.30*	-0.01 - 0.62	0.062	0.28†	-0.05 - 0.61	0.097
**CMAP amplitude** **(Proportion of baseline)**	MAP (mmHg)	0.04#	-0.00 - 0.08	0.052	0.02°	-0.03 - 0.07	0.661

* Corrected for side and muscle. Patient was added as random intercept and MAP was added as random slope
† Corrected for side, muscle, BIS and estimated plasma propofol concentration. Patient was added as random intercept and MAP was added as random slope.
# Corrected for side and muscle. Patient was added as random intercept and MAP could not be added as random slope.
° Corrected for side, muscle, BIS and estimated plasma propofol concentration. Patient was added as random intercept and MAP could not be added as random slope.
The estimates for H-reflex and CMAP amplitude are percentages.
