# Supplementary figures and images for: The Influence of Vasopressor-Induced Arterial Blood Pressure Elevation on Muscle-Recorded Motor Evoked Potentials
Source: Anesth Analg. 2025 Sep 5;142(4):730–40. doi: 10.1213/ANE.0000000000007701 (PMC12959597; doi:10.1213/ANE.0000000000007701)

## Supplementary material S2. CMAP 5-1 ratio's per patient per muscle per moment

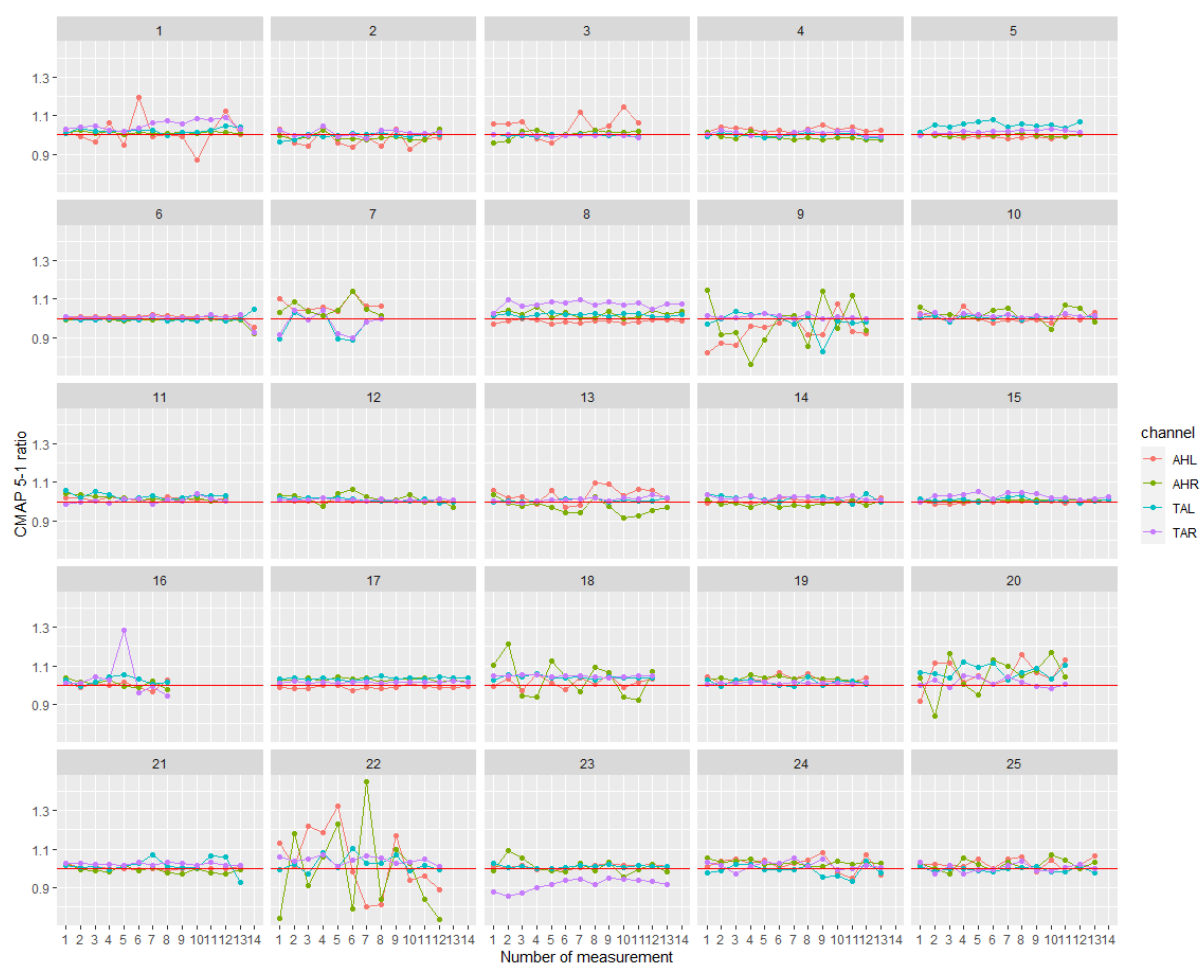

Supplement: Supplementary file 3 [file ane-142-730-s003.pdf]
